# Comparative efficacy of 5 sodium-glucose cotransporter protein-2 (SGLT-2) inhibitor and 4 glucagon-like peptide-1 (GLP-1) receptor agonist drugs in non-alcoholic fatty liver disease: A GRADE-assessed systematic review and network meta-analysis of randomized controlled trials

**DOI:** 10.3389/fphar.2023.1102792

**Published:** 2023-03-13

**Authors:** Yunpeng Gu, Lei Sun, Wei Zhang, Tingting Kong, Run Zhou, Yining He, Chaohua Deng, Luping Yang, Jianing Kong, Yutong Chen, Junping Shi, Yanli Hu

**Affiliations:** ^1^ School of Public Health, Hangzhou Normal University, Hangzhou, Zhejiang, China; ^2^ Medical School, Hangzhou Normal University, Hangzhou, Zhejiang, China; ^3^ School of Nursing, Hangzhou Normal University, Hangzhou, Zhejiang, China; ^4^ Medical School, Zhejiang Chinese Medical University, Hangzhou, Zhejiang, China; ^5^ The Department of Hepatology, The Affiliated Hospital of Hangzhou Normal University, Hangzhou, Zhejiang, China; ^6^ School of Nursing, Guangzhou Medical University, Guangzhou, Guangdong, China

**Keywords:** non-alcoholic fatty liver disease, SGLT-2 inhibitors, GLP-1 receptor agonists, semaglutide, liraglutide

## Abstract

**Background:** The relative efficacy of 5 sodium-glucose cotransporter protein-2 (SGLT-2) inhibitors and 4 glucagon-like peptide-1 (GLP-1) receptor agonists for non-alcoholic fatty liver disease (NAFLD) therapy has not been sufficiently investigated.

**Methods:** Randomized controlled trials (RCTs) in which patients with NAFLD were treated with SGLT-2 inhibitors or GLP-1 receptor agonists were included. Primary outcomes were improvements in liver enzymes and liver fat parameters, while secondary outcomes included anthropometric measures, blood lipids and glycemic parameters. The frequentist method was used to perform a network meta-analysis. Evidence certainty was assessed using the grading of recommendations assessment, development, and evaluation (GRADE).

**Results:** The criteria were satisfied by 37 RCTs with 9 interventions (5 SGLT-2 inhibitors and 4 GLP-1 receptor agonists). Based on high certainty evidence, in patients with NAFLD (or comorbid with type 2 diabetes), semaglutide could lower alanine aminotransferase as well as aspartate aminotransferase, γ-glutamyl transferase, controlled attenuation parameter, liver stiffness measurement, body weight, systolic blood pressure, triglycerides, high-density lipoprotein-cholesterol, glycosylated hemoglobin. Liraglutide could lower alanine aminotransferase as well as subcutaneous adipose tissue, body mass index, fasting blood glucose, glycosylated hemoglobin, glucose and homeostasis model assessment, while dapagliflozin could lower alanine aminotransferase as well as body weight, fasting blood glucose, postprandial blood glucose, glycosylated hemoglobin, glucose and homeostasis model assessment.

**Conclusion:** Semaglutide, liraglutide, and dapagliflozin all have a certain effect on NAFLD (or comorbid with type 2 diabetes) based on high confidence evidence from indirect comparisons, and semaglutide appears to have a therapeutic advantage over the other included medicines. Head-to-head studies are needed to provide more confidence in clinical decision-making.

## Introduction

Non-alcoholic fatty liver disease (NAFLD) is a disease spectrum that includes conditions from hepatic steatosis to hepatic necroinflammation (NASH, non-alcoholic steatohepatitis) (Schuppan and Schattenberg, 2013). The pathological mechanism of NAFLD has not yet been clarified, the two-hit hypothesis which includes insulin resistance, oxidative stress and lipid peroxidation, is considered to be the classic hypothesis of the pathogenesis of NAFLD. In recent years, the second-strike theory has been gradually replaced by the multiple-hit theory, but insulin resistance is still considered to be the key link in the strike (Loomba et al., 2021). With a global overall prevalence of up to 32.4%, NAFLD has become the leading cause of current chronic liver disease (Riazi et al., 2022). The rising incidence of NAFLD throughout the world is especially concerning because no medication to treat NAFLD or NASH has yet been approved. Currently, the first-line treatment for NAFLD is lifestyle management, especially dietary interventions (El-Agroudy et al., 2019). Emerging data from various stages of clinical trials show that some novel drugs targeting different molecular targets are promising candidates for the treatment of NAFLD (Negi et al., 2022). Based on the most recent American Association for the Study of Liver Diseases (AASLD) and European Association for the Study of Liver Diseases (EASL) guidelines, only pioglitazone and vitamin E are approved as NAFLD treatment options (EASL-EASD-EASO, 2016; Chalasani et al., 2018). Nevertheless, recent research has indicated that weight gain is the most prevalent adverse effect of pioglitazone medication, most likely due to enhanced adipose tissue insulin action and increased adipocyte TG synthesis (Sanyal et al., 2010; Cusi et al., 2016). Additionally, certain meta-analyses have found a link between pioglitazone and an increased risk of bladder cancer (Ferwana et al., 2013; Li et al., 2017; Davidson and Pan, 2018). There are also ongoing concerns regarding vitamin E’s long-term safety. Klein et al. (2011) discovered in a large RCT published in 2011 that dietary supplementation with Vitamin E substantially increased the risk of prostate cancer in healthy men.

Type 2 diabetes is known to be one of the most significant clinical risk factors for NAFLD development to NASH, cirrhosis, and hepatocellular carcinoma (Targher et al., 2018; Younossi et al., 2018; Mantovani et al., 2020b; Targher et al., 2021). A recent meta-analysis showed that the prevalence of elevated liver stiffness in Type 2 diabetes patients is 19.8%, which is much higher than the overall prevalence in the general adult population (Ciardullo and Perseghin, 2022). Many recent studies have demonstrated that some innovative glucose-lowering medicines (such as sodium-glucose cotransporter protein-2 inhibitors or glucagon-like peptide-1 receptor agonists) may improve liver injury and reduce liver fat in NAFLD patients (Fu et al., 2021; Kumar et al., 2021). Sodium-glucose cotransporter protein-2 (SGLT-2) inhibitors perform by assisting in renal glucose excretion, resulting in a reduction in body weight and obesity prevalence, which may improve the liver histology of patients with NAFLD/NASH (Brunton, 2015). Glucagon-like peptide-1 (GLP-1) receptor agonists are a new family of diabetes medications that enhance glycemic control through a variety of molecular pathways (Targher et al., 2018; Mantovani et al., 2020b). However, the relative efficacy of SGLT-2 inhibitors and GLP-1 receptor agonists for NAFLD treatment is difficult to discern from the literature, in part because there are few head-to-head comparison studies available and traditional pairwise meta-analysis cannot integrate all of the evidence from multiple comparators. Therefore, we did a systematic review and network meta-analysis (NMAs) to comprehensively review the literature and determine the relative efficacy of each of the SGLT-2 inhibitors and GLP-1 receptor agonists for NAFLD, which included the alterations in liver enzymes, liver fat parameters, anthropometric measures, as well as in blood lipids and glycemic parameters, while also to evaluate the evidence as to whether any medication is better than others.

## Methods

We conducted a systematic review and network meta-analysis to evaluate the efficacy of SGLT2 inhibitors and GLP-1 receptor agonists in NAFLD treatment. The results were provided in accordance with the PRISMA extension statement (Hutton et al., 2015).

### Systematic review registration

This review was not registered, and the protocol is supplied as a supplement (Supplementary Appendix S1).

### Search strategy

We used predefined keywords to systematically search four large electronic databases (PubMed, Embase, Web of Science, and Cochrane Library) for relevant papers published through 31 December 2021. There were no search restrictions. For potentially eligible studies, references from relevant literature were manually examined. The search strategy and the search free text terms utilized for the systematic review are included in the Supplementary Appendix S2, and were defined by all authors.

### Eligibility criteria

The following criteria were used to include studies in the systematic review and network meta-analysis: 1) Randomized controlled trials that compared, SGLT-2 inhibitors or GLP-1 receptor agonists against placebo or other active control drugs in NAFLD patients were included; and 2) Eligible studies must have reported at least one of the following outcomes: liver enzymes, liver fat parameters, anthropometric measures, blood lipids, and glycemic parameters. The following are the exclusion criteria: 1) observational or non-randomized intervention studies; 2) trials involving children or adolescents (under the age of 18); and 3) non-English literature.

To confirm that all data were obtained consistently across studies, two independent investigators (any two of YPG, TTK, RZ, YNH, CHD, LPY, YTC, LS, or YLH) reviewed studies using a specific data case report form and data dictionary. Articles were first assessed by title and abstract, followed by full-text articles to identify eligible studies. Any disagreements were addressed by consensus with a third reviewer (JPS).

### Outcomes

Primary outcomes were improvements in liver enzymes [alanine aminotransferase (ALT), aspartate aminotransferase (AST), γ-glutamyl transferase (GGT) ] and liver fat parameters [subcutaneous adipose tissue (SAT), visceral adipose tissue (VAT), liver fat fraction (LFF), controlled attenuation parameter (CAP), liver stiffness measurement (LSM)]; secondary outcomes included anthropometric measures [body weight (BW), body mass index (BMI), waist circumference (WC), systolic blood pressure (SBP), diastolic blood pressure (DBP) ], blood lipids [total cholesterol (TC), triglycerides (TG), high density lipoprotein-cholesterol (HDL-C), low density lipoprotein-cholesterol (LDL-C) and serum adiponectin], glycemic parameters [fasting blood glucose (FBG), postprandial blood glucose (PBG), glycosylated hemoglobin (HbA1c), glucose and homeostasis model assessment (HOMA-IR)].

### Data extraction and risk of bias assessment

Data extraction was done independently by two groups of investigators (YPG, LS, TTK, YNH and RZ, LPY, CHD, YTC). We extracted data on the first author, publication year, study country, number of participants, primary participant characteristics, types of therapies (including daily doses of medications used), duration of therapy, methods used to diagnose NAFLD, and results from all studies. Each relevant article was evaluated for quality by two independent reviewers (LS, TTK, and RZ), with discrepancies addressed by consensus. The risk of bias for each research was examined using the Cochrane risk of bias tool, which determined whether studies were at low, high, or unclear risk (Higgins and Cochrane Collaboration, 2020).

### Data synthesis and statistical analysis

We used the frequentist method to conduct a network meta-analysis to compare the effects of SGLT-2 inhibitors and GLP-1 receptor agonists on NAFLD (Rücker, 2012). For continuous variables, we calculated the normalized mean difference (MD), and for dichotomous variables, we calculated the odds ratios (ORs). All results were expressed by 95% confidence interval (CI). The mean differences between the intervention and control arms, as well as their standard deviations, were calculated and utilized as the foundation for each trial comparison. A generalized Q test was used to measure heterogeneity across individual studies and global inconsistency across different treatment comparison designs in the network, with *p* values less than 0.05 representing substantial heterogeneity (Krahn et al., 2013). Node splitting analysis was used to investigate any local inconsistencies between direct and indirect evidence within each treatment comparison (Dias et al., 2010). The pooled mean differences and 95% CI for each pair of treatment comparisons were calculated using a fixed-effects consistency model. In an analysis including 10 or more trials, we used funnel plots for evidence of small-study effects.

We evaluated the relative ranking probability of treatment effects of all therapies for the target outcomes to provide additional information for clinical applications. The surface under the cumulative ranking curve (SUCRA) measures the proportion of each treatment’s mean rank relative to an illustrative intervention that is the best without ambiguity (Salanti et al., 2011).

To include multi-arm studies in our meta-analysis, we aggregated all relevant control groups into a single control group (active control group), performing a single pair-wise comparison, as recommended by the Cochrane Handbook for Systematic Reviews of Interventions (Higgins and Cochrane Collaboration, 2020). All pairwise and network meta-analyses were conducted in Stata 15 (StataCorp, College Station, TX) with network package and published routines (Chaimani et al., 2013).

### Assessment of evidence certainty

The grading of recommendations assessment, development, and evaluation (GRADE) technique was used to assess the certainty of evidence (Atkins et al., 2004; Brignardello-Petersen et al., 2018; Brignardello-Petersen et al., 2020). Including specific guidance for network meta-analyses (Puhan et al., 2014). Two investigators with GRADE expertise assessed each domain individually for each comparison and outcome, and any conflicts were addressed through discussion. Criteria for classifying the certainty of each comparison and outcome as high, moderate, low, or very low, included considerations of risk of bias (failure to conceal random allocation or blind participants in randomized controlled trials or failure to adequately control for confounding in observational studies), inconsistency (heterogeneity of estimates of effects across trials), indirectness (surrogate outcomes, study populations or interventions that differ from those of interest, or intransitivity), imprecision and publication bias (Puhan et al., 2014). The network quality ranking was based on that estimate if only direct or indirect information is provided for specific comparison. When both direct and indirect evidence were available for a specific comparison, the estimate that supplied the most information (direct or indirect) served as the foundation for the certainty of the network estimates. We picked the highest of the two certainty assessments if both estimates offered equivalent quantities of information. If there was evidence of incoherence between direct and indirect estimates, the certainty of the network estimate was rated down (Puhan et al., 2014).

## Result

### Systematic review and characteristic

**FIGURE 1:**
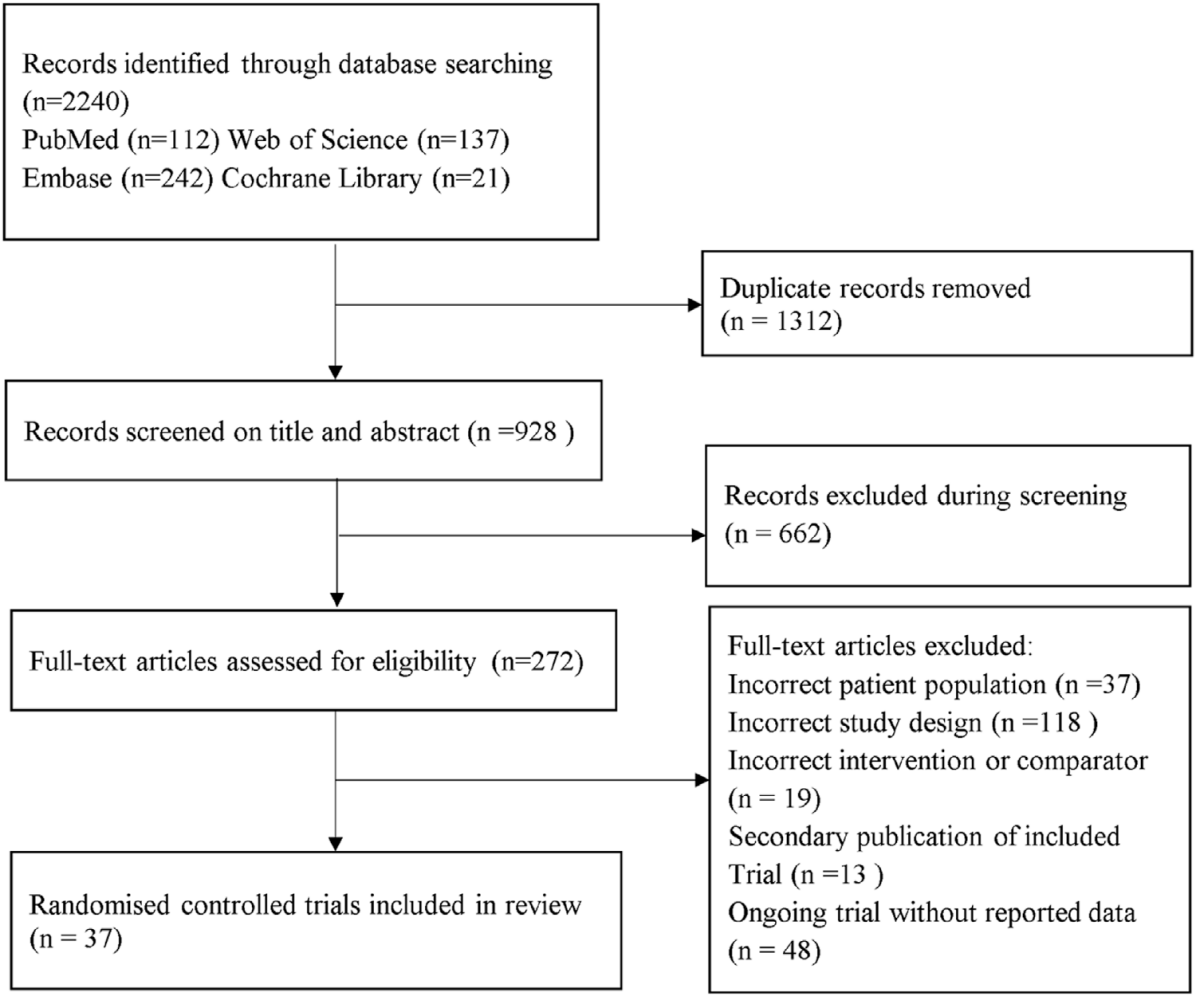
PRISMA flow diagram.

**FIGURE 2:**
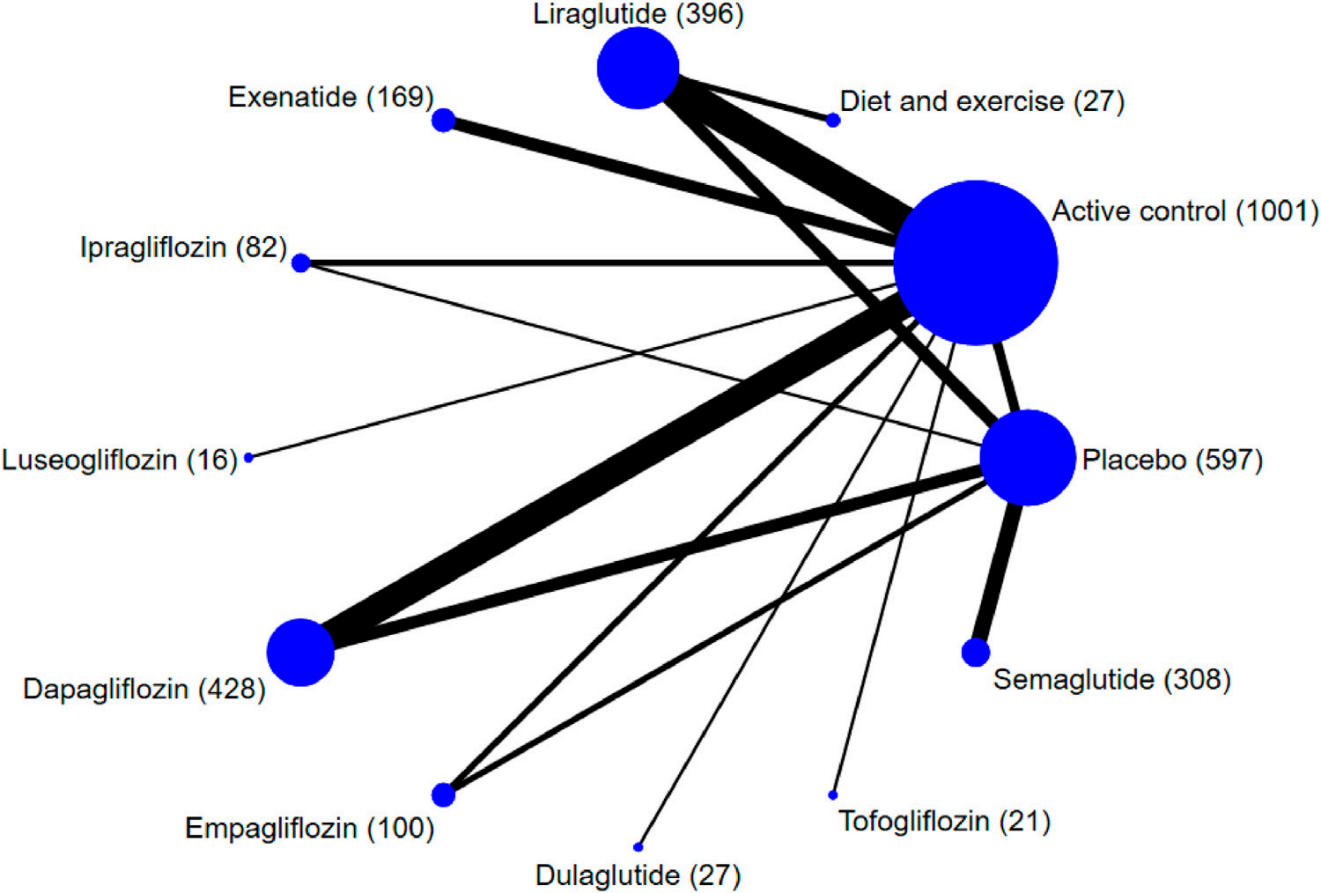
Network plot of trials evaluating the effects of sodium-glucose cotransporter protein-2 inhibitors and glucagon-like peptide-1 receptor agonists for patients with NAFLD. Network shows the number of participants assigned to each treatment class with the size of each circle proportional to the number of randomly assigned participants in the treatment comparisons (sample size for the specific treatment shown in brackets). Line thickness is proportional to the number of patients that contributed to the comparison.

A total of 2,240 records were identified from the initial title and abstract screening and 272 were then obtained (Figure 1). Finally, 37 randomized controlled trials with a total of 3,172 patients enrolled were deemed eligible for inclusion (Ohki et al., 2012; Fan et al., 2013; Shao et al., 2014; Armstrong et al., 2016a; Armstrong et al., 2016b; Savvidou et al., 2016; Feng et al., 2017; Ito et al., 2017; Khoo et al., 2017; Eriksson et al., 2018; Kuchay et al., 2018; Shibuya et al., 2018; Tian et al., 2018; Aso et al., 2019; Khoo et al., 2019; Shimizu et al., 2019; Yan et al., 2019; Guo et al., 2020; Han et al., 2020; Jiang and Chen, 2020; Kinoshita et al., 2020; Kuchay et al., 2020; Liu et al., 2020; Pang et al., 2020; Taheri et al., 2020; Vedtofte et al., 2020; Zhang et al., 2020; Aso et al., 2021; Chehrehgosha et al., 2021; Cho et al., 2021; Flint et al., 2021; Hussain et al., 2021; Newsome et al., 2021; Phrueksotsai et al., 2021; Takahashi et al., 2021; Tobita et al., 2021; Yoneda et al., 2021), with 11 different treatments including SGLT-2 inhibitors (dapagliflozin, ipragliflozin, luseogliflozin, tofogliflozin, empagliflozin), GLP-1 receptor agonists (exenatide, liraglutide, dulaglutide, semaglutide), placebo, and active controls (pioglitazone, metformin, intensive insulin, etc.). Figure 2 shows the networks, and the main characteristic of all research was summarized in Supplementary Appendix S3.

### Risk of bias

The risk of bias in each study is presented in Supplementary Appendix S5. The main drawback was the deviations from the intended interventions. Thirty-three trials (89.2%) had a low risk of bias in random sequence generation, while twenty trials (54.1%) had a low risk of bias in deviations from the intended interventions. Twenty-eight trials (75.7%) were found to be at low risk of bias in measurement of the outcome and thirty-one trials (83.8%) for missing outcome data, while twenty-seven trials (73.0%) were found to be at low risk of selective outcome reporting bias.

### Outcomes

The network plot for each result is shown in Supplementary Appendix S6. Except for HOMA-IR (Supplementary Appendix S7), no evidence of global network inconsistency was observed; we attempted to identify the cause of inconsistency using node splitting analysis (Supplementary Appendix S8, Supplementary Table S22), but we were unable to do so. We utilized the direct estimate as the best estimate of the treatment effect to assure the accuracy of the HOMA-IR outcome. The funnel plot revealed no significant risk of publication bias (Supplementary Appendix S10). Supplementary Appendix S9 provides network estimates for all outcomes for each drug comparison (all drugs ranked according to SUCRA ranking). Table 1 and Supplementary Appendix S11 illustrate the expected absolute differences in therapy with each of the SGLT-2 inhibitors or GLP-1 receptor agonists compared to placebo and each other.

**TABLE 1:** Summary of anticipated absolute differences in outcomes comparing sodium-glucose cotransporter-2 inhibitor and glucagon-like peptide-1 receptor agonist treatment with placebo treatment.

Liver enzymes parameters	Alanine aminotransferase (ALT)			Aspartate aminotransferase (AST)				γ-glutamyl transferase (GGT)
Dapagliflozin	−9.94 U/L (−18.42,−1.46)			−6.70 U/L (−12.03,−1.37)				−13.82 U/L (−31.20,3.57)
	㊉㊉㊉㊉			㊉㊉㊉㊀				㊉㊉㊀㊀
Empagliflozin	−5.37 U/L (−17.17,6.43)			−5.08 U/L (−12.07,1.92)				−16.60 U/L (−49.74,16.53)
	㊉㊉㊉㊉			㊉㊉㊉㊉				㊉㊉㊉㊀
Ipragliflozin	−8.09 U/L (−20.72,4.54)			−7.08 U/L (−14.76,0.60)				−10.37 U/L (−26.04,5.31)
	㊉㊉㊀㊀			㊉㊉㊀㊀				㊉㊉㊉㊀
Luseogliflozin	0.69 U/L (−20.37,21.75)			NR				NR
	㊉㊉㊀㊀							
Tofogliflozin	6.69 U/L (−19.27,32.65)			8.06 U/L (−7.03,23.14)				23.80 U/L (−7.21,54.80)
	㊉㊉㊉㊀			㊉㊉㊉㊀				㊉㊉㊉㊀
Dulaglutide	−16.31 U/L (−40.22,7.60)			−13.34 U/L (−28.22,1.53)				−18.70 U/L (−45.13,7.73)
	㊉㊉㊉㊀			㊉㊉㊉㊀				㊉㊉㊉㊀
Exenatide	−9.65 U/L (−21.65,2.35)			−8.50 U/L (−15.70,-1.29)				−7.29 U/L (−26.43,11.85)
	㊉㊉㊀㊀			㊉㊉㊀㊀				㊉㊉㊀㊀
Liraglutide	−8.30 U/L (−16.16, −0.43)			−4.60 U/L (−9.48,0.29)				−7.29 U/L (−19.88,5.30)
	㊉㊉㊉㊉			㊉㊉㊉㊉				㊉㊉㊉㊉
Semaglutide	−14.70 U/L (−24.79,−4.61)			−9.32 U/L (−15.12,−3.52)				−16.56 U/L (−27.30,−5.82)
	㊉㊉㊉㊉			㊉㊉㊉㊉				㊉㊉㊉㊉

Liver fat parameters	Subcutaneous adipose tissue (SAT)	Visceral adipose tissue (VAT)		Liver fat fraction (LFF)		Controlled attenuation parameter (CAP)		Liver stiffness measurement (LSM)
Dapagliflozin	−0.26 cm^2^ (−0.36,−0.17)	−6.96 cm^2^ (−18.37,4.46)		−0.95% (−2.52,0.62)		−38.86 db/m (−73.39,-4.33)		−1.67 kPa (−4.49,1.16)
	㊉㊉㊉㊀	㊉㊉㊀㊀		㊉㊉㊉㊀		㊉㊉㊀㊀		㊉㊉㊉㊀
Empagliflozin	NR	−11.45 cm^2^ (−30.12,7.22)		NR		−9.04 db/m (−26.88,8.81)		−0.49 kPa (−1.11,0.12)
		㊉㊉㊉㊉				㊉㊉㊉㊉		㊉㊉㊉㊀
Ipragliflozin	−7.96 cm^2^ (−11.60,−4.33)	−25.13 cm^2^ (−44.49,−5.76)		NR		−19.70 db/m (−41.86,2.46)		NR
	㊉㊉㊉㊀	㊉㊉㊀㊀				㊉㊉㊀㊀		
Luseogliflozin	NR	NR		NR		NR		NR
Tofogliflozin	NR	NR		1.08% (−3.45,5.61)		NR		0.46 kPa (−0.89,1.82)
				㊉㊉㊉㊀				㊉㊉㊉㊀
Dulaglutide	NR	NR		NR		NR		−1.05 kPa (−3.07,0.97)
								㊉㊉㊉㊀
Exenatide	−30.93 cm^2^ (−55.67,-6.20)	−40.26 cm^2^ (−74.32,−6.21)		NR		NR		NR
	㊉㊀㊀㊀	㊉㊀㊀㊀						
Liraglutide	−30.27 cm^2^ (-41.54,-19.01)	−30.12 cm^2^ (−45.36,-14.89)		−5.16% (−7.51,−2.81)		−16.70 db/m (−80.22,46.82)		NR
	㊉㊉㊉㊉	㊉㊉㊉㊀		㊉㊉㊀㊀		㊉㊉㊉㊉		
Semaglutide	NR	NR		NR		−15.57 db/m (−29.29,-1.85)		−3.08 kPa (−3.39,−2.77)
						㊉㊉㊉㊉		㊉㊉㊉㊉

Anthropometric measures	Body weight (BW)	Body mass index (BMI)		Waist circumference (WC)		Systolic blood pressure (SBP)		Diastolic blood pressure (DBP)
Dapagliflozin	−3.48 kg (−5.88,-1.08)	−1.13 kg/m^2^ (−2.14,-0.11)		−2.78 cm (−5.61,0.06)		−3.61 mmHg (−9.84,2.61)		−1.59 mmHg (−5.41,2.23)
	㊉㊉㊉㊉	㊉㊉㊉㊀		㊉㊉㊉㊉		㊉㊉㊉㊀		㊉㊉㊉㊀
Empagliflozin	−2.60 kg (−7.31,2.12)	−1.07 kg/m^2^ (−2.45,0.31)		−1.30 cm (−7.54,4.94)		5.09 mmHg (−4.90,15.09)		1.93 mmHg (−4.47,8.33)
	㊉㊉㊉㊉	㊉㊉㊉㊉		㊉㊉㊉㊉		㊉㊉㊉㊀		㊉㊉㊉㊀
Ipragliflozin	−3.04 kg (−7.54,1.47)	−1.07 kg/m^2^ (−2.64,0.50)		−3.70 cm (−8.80,1.40)		−3.90 mmHg (−6.79,-1.01)		0.33 mmHg (−1.50,2.17)
	㊉㊉㊉㊀	㊉㊉㊉㊀		㊉㊉㊀㊀		㊉㊉㊀㊀		㊉㊉㊀㊀
Luseogliflozin	NR	−0.83 kg/m^2^ (−2.83,1.16)		NR		NR		NR
		㊉㊀㊀㊀						
Tofogliflozin	1.60 kg (−4.71,7.90)	NR		NR		NR		NR
	㊉㊉㊉㊀							
Dulaglutide	−2.14 kg (−8.45,4.16)	−1.00 kg/m^2^ (−3.10,1.09)		NR		NR		NR
	㊉㊉㊉㊀	㊉㊉㊉㊀						
Exenatide	−4.40 kg (−8.12,−0.69)	−1.86 kg/m^2^ (−3.12,-0.59)		−6.12 cm (−10.26,−1.97)		−3.11 mmHg (−8.46,2.25)		−0.77 mmHg (−4.14,2.60)
	㊉㊉㊀㊀	㊉㊉㊀㊀		㊉㊉㊀㊀		㊉㊀㊀㊀		㊉㊀㊀㊀
Liraglutide	−3.75 kg (−6.34,-1.15)	−1.49 kg/m^2^ (−2.39,-0.59)		−4.53 cm (−7.37,−1.69)		−3.27 mmHg (−7.45,0.91)		0.24 mmHg (−2.76,3.24)
	㊉㊉㊉㊀	㊉㊉㊉㊉		㊉㊉㊉㊀		㊉㊉㊉㊉		㊉㊉㊉㊉
Semaglutide	−8.14 kg (−11.45,−4.84)	NR		NR		−2.24 mmHg (−4.20,-0.27)		−0.38 mmHg (−1.41,0.64)
	㊉㊉㊉㊉					㊉㊉㊉㊉		㊉㊉㊉㊉

Blood lipids	Total cholesterol(TC)	Triglycerides (TG)		High density lipoprotein-cholesterol (HDL-C)		Low density lipoprotein-cholesterol (LDL-C)		Serum adiponectin
Dapagliflozin	0.30 mmol/L (−0.19,0.78)	−0.23 mmol/L (−0.46,-0.01)		0.14 mmol/L (0.06,0.21)		−0.03 mmol/L (−0.37,0.31)		−1.61 μg/mL (−5.02,1.81)
	㊉㊀㊀㊀	㊉㊉㊀㊀		㊉㊉㊀㊀		㊉㊉㊉㊉		㊉㊉㊉㊀
Empagliflozin	−0.23 mmol/L (−0.76,0.30)	−0.09 mmol/L (−0.60,0.42)		0.03 mmol/L (−0.07,0.14)		−0.09 mmol/L (−0.55,0.37)		NR
	㊉㊉㊉㊉	㊉㊉㊉㊉		㊉㊉㊉㊉		㊉㊉㊉㊉		
Ipragliflozin	0.05 mmol/L (−0.28,0.38)	−0.26 mmol/L (−0.48,-0.04)		0.08 mmol/L (0.01,0.14)		0.06 mmol/L (−0.33,0.46)		−7.18 μg/mL (−13.13,-1.23)
	㊉㊉㊀㊀	㊉㊉㊉㊀		㊉㊉㊉㊀		㊉㊉㊉㊀		㊉㊀㊀㊀
Luseogliflozin	NR	NR		NR		NR		NR
Tofogliflozin	−0.01 mmol/L (−0.81,0.79)	0.52 mmol/L (−0.12,1.16)		NR		NR		NR
	㊉㊉㊉㊀	㊉㊉㊉㊀						
Dulaglutide		−0.35 mmol/L (−2.76,2.07)		−0.02 mmol/L (−0.58,0.54)		0.10 mmol/L (−2.11,2.31)		NR
		㊉㊉㊉㊀		㊉㊉㊉㊀		㊉㊉㊉㊀		
Exenatide	−0.33 mmol/L (−0.74,0.07)	−0.21 mmol/L (−0.48,0.06)		0.07 mmol/L (−0.02,0.17)		−0.28 mmol/L (−0.73,0.18)		−1.59 μg/mL (−6.13,2.96)
	㊉㊉㊀㊀	㊉㊉㊀㊀		㊉㊉㊉㊀		㊉㊉㊉㊀		㊉㊉㊉㊀
Liraglutide	−0.24 mmol/L (−0.55,0.06)	−0.20 mmol/L (−0.43,0.03)		0.08 mmol/L (0.02,0.15)		−0.05 mmol/L (−0.35,0.25)		3.03 μg/mL (−0.43,6.49)
	㊉㊉㊉㊀	㊉㊉㊉㊀		㊉㊉㊉㊀		㊉㊉㊉㊀		㊉㊉㊉㊉
Semaglutide	0.13 mmol/L (−0.13,0.39)	−0.25 mmol/L (−0.40,−0.10)		0.05 mmol/L (0.01,0.09)		0.16 mmol/L (−0.16,0.48)		NR
	㊉㊉㊉㊉	㊉㊉㊉㊉		㊉㊉㊉㊉		㊉㊉㊉㊉		

Glycemic parameters	Fasting blood glucose (FBG)		Postprandial blood glucose (PBG)		Glycosylated hemoglobin (HbA1c)		Glucose and homeostasis model assessment (HOMA-IR)	
Dapagliflozin	−0.75 mmol/L (−1.12,−0.39)		−2.14 mmol/L (−3.67,-0.61)		−0.72% (−1.01,−0.42)		−0.84 (−1.53,−0.15)	
	㊉㊉㊉㊉		㊉㊉㊉㊉		㊉㊉㊉㊉		㊉㊉㊉㊉	
Empagliflozin	0.06 mmol/L (−0.44,0.55)		NR		−0.12% (−0.64,0.39)		−0.11 (−0.49,0.27)	
	㊉㊉㊉㊀				㊉㊉㊉㊀		㊉㊉㊉㊀	
Ipragliflozin	−0.33 mmol/L (−0.84,0.18)		NR		−0.36% (−0.77,0.04)		−0.60 (−1.01,−0.19)	
	㊉㊉㊉㊀				㊉㊉㊉㊀		㊉㊉㊉㊀	
Luseogliflozin	−2.32 mmol/L (−16.74,12.10)		NR		−1.05% (−1.80,−0.30)		NR	
	㊉㊀㊀㊀				㊉㊀㊀㊀			
Tofogliflozin	0.67 mmol/L (−0.95,2.29)		NR		−0.06% (−0.67,0.55)		NR	
	㊉㊉㊉㊀				㊉㊉㊉㊀			
Dulaglutide	−1.32 mmol/L (−10.13,7.49)		NR		−0.65% (−1.47,0.17)		NR	
	㊉㊉㊉㊀				㊉㊉㊉㊀			
Exenatide	−0.36 mmol/L (−0.91,0.20)		−0.15 mmol/L (−2.32,2.02)		−0.41% (−0.82,0.00)		−0.34 (−1.53,0.86)	
	㊉㊉㊀㊀		㊉㊉㊀㊀		㊉㊉㊀㊀		㊉㊉㊉㊀	
Liraglutide	−0.77 mmol/L (−1.19,−0.35)		−1.70 mmol/L (−3.74,0.34)		−0.50% (−0.81,−0.19)		−1.57 (−2.18,−0.96)	
	㊉㊉㊉㊉		㊉㊉㊉㊀		㊉㊉㊉㊉		㊉㊉㊉㊉	
Semaglutide	−1.35 mmol/L (−3.01,0.32)		NR		−0.93% (−1.23,−0.63)		−0.29 (−1.19,0.61)	
	㊉㊉㊉㊉				㊉㊉㊉㊉		㊉㊉㊉㊉	

Certainty of the evidence for each estimate is shown: high certainty㊉㊉㊉㊉; moderate certainty㊉㊉㊉㊀; low certainty㊉㊉㊀㊀; very low certainty㊉㊀㊀㊀.

NR: not report.

#### Liver enzymes parameters

Alanine aminotransferase (ALT) was reported in 35 studies involving 2,950 subjects. Semaglutide (MD = −14.70 U/L; 95%CI: −24.79, −4.61; high certainty), dapagliflozin (MD = −9.94 U/L; 95%CI: −18.42, −1.46; high certainty) and liraglutide (MD = −8.30 U/L; 95%CI: −16.61, −0.43; high certainty) could reduce ALT levels compared to placebo.

Thirty-four trials including 2,714 patients reported aspartate aminotransferase (AST). Semaglutide (MD = −9.32 U/L; 95%CI: −15.12, −3.52; high certainty), exenatide (MD = −8.50 U/L; 95%CI: −15.70, −1.29; low certainty) and dapagliflozin (MD = −6.70 U/L; 95%CI: −12.03, −1.37; moderate certainty) could reduce AST levels compared to placebo.

Twenty-seven trials including 1956 patients reported γ-glutamyl transferase (GGT). Only semaglutide (MD = −16.56 U/L; 95%CI: −27.30, −5.82; high certainty) could reduce GGT levels compared to placebo.

#### Liver fat parameters

Ten trials including 550 patients reported subcutaneous adipose tissue (SAT). Exenatide (MD = −30.93 cm^2^; 95%CI: −55.67, −6.20; very low certainty), liraglutide (MD = −30.27 cm^2^; 95%CI: −41.54, −19.01; high certainty), ipragliflozin (MD = −7.96 cm^2^; 95%CI: −11.60, −4.33; moderate certainty) and dapagliflozin (MD = −0.26 cm^2^; 95%CI: −0.36, −0.17; moderate certainty) could reduce SAT levels compared to placebo.

Fourteen trials including 1,007 patients reported subcutaneous adipose tissue (VAT). Exenatide [MD = −40.26 cm^2^; 95%CI: −74.32, −6.21; very low certainty), liraglutide (MD = −30.12 cm^2^; 95%CI: −45.36, −14.89; moderate certainty)] and ipragliflozin (MD = −25.13 cm^2^; 95%CI: −44.49, −5.76; low certainty) could reduce VAT levels compared to placebo.

Six trials including 298 patients reported liver fat fraction (LFF). Only liraglutide (MD = −5.16%; 95%CI: −7.51, −2.81; low certainty) could reduce LFF levels compared to placebo.

Nine trials including 852 patients reported controlled attenuation parameter (CAP). Dapagliflozin (MD = −38.86 db/m; 95%CI: −73.39, −4.33; low certainty) and semaglutide (MD = −15.57 db/m; 95%CI: −29.29, −1.85; high certainty) could reduce CAP levels compared to placebo.

Eight trials including 825 patients reported liver stiffness measurement (LSM). Semaglutide (MD = −3.08 kPa; 95%CI: −3.39, −2.77; high certainty) could reduce LSM levels compared to placebo.

#### Anthropometric measures

Thirty-two trials including 2,668 patients reported body weight (BW). Semaglutide (MD = −8.14 kg; 95%CI: −11.45, −4.84; high certainty), exenatide (MD = −4.40 kg; 95%CI: −8.12, −0.69; low certainty), liraglutide (MD = −3.75 kg; 95%CI: −6.34, −1.15; moderate certainty) and dapagliflozin (MD = −3.48 kg; 95%CI: −5.88, −1.08; high certainty) could reduce BW levels compared to placebo.

Thirty-two trials including 2,163 patients reported body mass index (BMI). Exenatide (MD = −1.86 kg/m^2^; 95%CI: −3.12, −0.59; low certainty), liraglutide (MD = −1.49 kg/m^2^; 95%CI: −2.39, −0.59; high certainty) and dapagliflozin (MD = −1.13 kg/m^2^; 95%CI: −2.14, −0.11; moderate certainty) could reduce BMI levels compared to placebo.

Twenty trials including 1,048 patients reported waist circumference (WC). Exenatide (MD = −6.12 cm; 95%CI: −10.26, −1.97; low certainty) and liraglutide (MD = −4.53 cm; 95%CI: −7.37, −1.69; moderate certainty) could reduce WC levels compared to placebo.

Twenty trials including 1,383 patients reported systolic blood pressure (SBP). Ipragliflozin (MD = −3.90 mmHg; 95%CI: −6.79, −1.01; low certainty) and semaglutide (MD = −2.24 mmHg; 95%CI: −4.20, −0.27; high certainty) could reduce SBP levels compared to placebo.

Nineteen trials including 1,369 patients reported diastolic blood pressure (DBP). No statistically significant results were observed compared to placebo.

#### Blood lipids

Twenty-three trials including 1939 patients reported total cholesterol (TC). No statistically significant results were observed compared to placebo.

Thirty-four trials including 2,502 patients reported triglycerides (TG). Ipragliflozin (MD = −0.26 mmol/L; 95%CI: −0.48, −0.04; moderate certainty) and semaglutide (MD = −0.25 mmol/L; 95%CI: −0.40, −0.10; high certainty) could reduce TG levels compared to placebo.

Thirty trials including 2,294 patients reported high density lipoprotein-cholesterol (HDL-C). Dapagliflozin (MD = 0.14 mmol/L; 95%CI: 0.06, 0.21; low certainty), liraglutide (MD = 0.08 mmol/L; 95%CI: 0.02, 0.15; moderate certainty), ipragliflozin (MD = 0.08 mmol/L; 95%CI: 0.01, 0.14; moderate certainty) and semaglutide (MD = 0.05 mmol/L; 95%CI: 0.01, 0.09; high certainty) could increase HDL-C levels compared to placebo.

Thirty-two trials including 2,402 patients reported low-density lipoprotein-cholesterol (LDL-C). No statistically significant results were observed compared to placebo.

Thirteen trials including 907 patients reported serum adiponectin. Ipragliflozin (MD = −7.18 μg/mL; 95%CI: −13.13, −1.23; very low certainty) could reduce adiponectin levels compared to placebo.

#### Glycemic parameters

Thirty-one trials including 2,184 patients reported fasting blood glucose (FBG). Liraglutide (MD = −0.77 mmol/L; 95%CI: −1.19, −0.35; high certainty) and dapagliflozin (MD = −0.75 mmol/L; 95%CI: −1.12, −0.39; high certainty) could reduce FBG levels compared with placebo.

Eleven trials including 738 patients reported postprandial blood glucose (PBG). Dapagliflozin (MD = −2.14 mmol/L; 95%CI: −3.67, −0.61; high certainty) could reduce PBG levels compared with placebo.

Thirty-one trials including 3,009 patients reported glycosylated hemoglobin (HbA1c). Luseogliflozin (MD = −1.05%; 95%CI: −1.80, −0.30; very low certainty), semaglutide (MD = −0.93%; 95%CI: −1.23, −0.63; high certainty), dapagliflozin (MD = −0.72%; 95%CI: −1.01, −0.42; high certainty) and liraglutide (MD = −0.50%; 95%CI: −0.81, −0.19; high certainty) could reduce HbA1c levels compared with placebo.

Twenty-six trials including 1728 patients reported glucose and homeostasis model assessment (HOMA-IR). Due to the global network inconsistency, we utilized the direct estimate as the best estimate of the treatment effect. Liraglutide (MD = −1.57; 95%CI: −2.18, −0.96; high certainty), dapagliflozin (MD = −0.84; 95%CI: −1.53, −0.15; high certainty) and ipragliflozin (MD = −0.60; 95%CI: −1.01, −0.19; moderate certainty) reduced HOMA-IR levels more than placebo.

#### Safety and adverse events

Adverse events (AEs) were reported in 18 of the 37 included trials (3 for dapagliflozin, 3 for empagliflozin, 1 for ipragliflozin, 0 for luseogliflozin, 1 for tofogliflozin, 1 for dulaglutide, 1 for exenatide, 6 for liraglutide and 2 for semaglutide). Given the insufficient number of AEs reported for each intervention, meta-analyses could not be performed. Table 2 provides summary information regarding the safety of the nine interventions. Most adverse events were mild to moderate in severity, and no deaths were reported during the trial of the nine interventions. Gastrointestinal disorders were the most commonly reported AEs of GLP-1 receptor agonists. Urogenital infections, including urinary tract infections, balanoposthitis and vaginitis, were the common AEs of SGLT-2 inhibitors.

**TABLE 2:** Summary of adverse events.

Type of intervention	Study	Number of patients	Type of adverse events
Dapagliflozin	Eriksson et al.	21	Adverse event (unclassified) 7 (33.3%)
	Pang et al.	103	Mild hypoglycemia 1 (1%); Urinary tract infection 1 (1%)
	Hussain et al.	75	Hypoglycemia 3 (4%); Frequency of urine 5 (6.7%)
Empagliflozin	Kuchay et al.	22	Balanoposthitis 1 (4.5%); Non-specific fatigue 1 (4.5%); Arthralgia of the big joints 1 (4.5%)
	Taheri et al.	43	Mild fungal vaginal infections 2 (4.7%); Mild allergic reactions 1 (2.3%)
	Chehrehgosha et al.	35	Mild hypoglycemia 1 (2.9%); Urticaria 1 (2.9%); Nocturia and polyuria 1 (2.9%); Severe weakness and fatigue 1 (2.9%)
Ipragliflozin	Takahashi et al.	27	Constipation 1 (3.7%); Frequency urination 2 (7.4%); Knee osteoarthritis 1 (3.7%); Perineum pruritus 1 (3.7%)
Luseogliflozin	None		
Tofogliflozin	Yoneda et al.	21	Urinary tract infection 1 (4.8%)
Dulaglutide	Kuchay et al.	32	Upper gastrointestinal upset 5 (15.6%); Transient diarrhoea 1 (3.1%)
Exenatide	Liu et al.	38	Hypoglycemia 3 (7.9%)
Liraglutide	Armstrong et al.	26	Gastrointestinal disorders 21 (81%); Nausea 12 (46%); Diarrhoea 10 (38%); Abdominal pain 8 (31%); Vomiting 5 (19%); Constipation 7 (27%); Dyspepsia 4 (15%); Flatulence 4 (15%); Bloating 4 (15%); Eye disorders 1 (4%); Cardiac disorders 3 (12%); General disorders and administration site conditions 13 (50%); General disorders and administration site conditions 13 (50%); Fatigue 4 (15%); Influenza-like symptoms 3 (12%); Peripheral oedema 2 (8%); Chills 4 (15%); Non-specific pain 2 (8%); Infections and infestations 3 (12%); Investigations 5 (19%); Increased aspartate aminotransferase 1 (4%); Metabolism and nutrition disorders 11 (42%); Anorexia (loss of appetite) 8 (31%); Musculoskeletal and connective disorders 8 (31%); Back pain 3 (12%); Arthralgia 1 (4%); Nervous system disorders 14 (54%); Dizziness 6 (23%); Headaches or migraines 9 (35%); Psychiatric disorders 6 (23%); Depression 2 (8%); Renal and urinary disorders 2 (8%); Respiratory, thoracic, and mediastinal disorders 3 (12%); Cough 2 (8%); Skin and soft tissue disorders 7 (27%)
	Khoo et al.	15	Nausea 12 (80.0%); Abdominal discomfort and bloating 15 (100%); Diarrhoea 5 (33.3%); Flatulence 6 (40.0%); Constipation 1 (6.7%); Dizziness 2 (13.3%); Muscle aches 1 (6.7%); Injection site reaction 1 (6.7%)
	Yan et al.	24	Nausea and vomiting 4 (16.7%); Headache 1 (4.2%)
	Zhang et al.	30	Gastrointestinal reactions 9 (30%); Hypoglycemia 1 (3.3%); Heart failure 0
	Guo et al.	31	Nausea and vomiting 8 (25.8%); Diarrhea 1 (3.2%); Non-severe hypoglycemia 1 (3.2%)
	Jiang et al.	58	Nausea or vomiting 4 (6.9%); Diarrhoea 3 (5.2%)
Semaglutide	Flint et al.	33	Decreased appetite 14 (42.4%); Diarrhoea 10 (30.3%); Nausea 10 (30.3%); Vomiting 9 (27.3%); Nasopharyngitis 8 (24.2%); Constipation 8 (24.2%); Abdominal pain upper 6 (18.2%); Dizziness 6 (18.2%); Flatulence 6 (18.2%); Eructation 5 (15.2%); Headache 4 (12.1%); Fatigue 4 (12.1%); Early satiety 4 (12.1%)
	Newsome et al.	239	Nausea 87 (36.4%); Constipation 48 (20.1%); Decreased appetite 52 (21.8%); Diarrhea 61 (25.5%); Vomiting 43 (18.0%); Back pain 22 (9.2%); Headache 27 (11.3%); Nasopharyngitis 36 (15.1%); Arthralgia 13 (5.4%); Fatigue 22 (9.2%); Abdominal pain 23 (9.6%); Abdominal distension 13 (5.4%); Dyspepsia 17 (7.1%)

## Discussion

### Principal findings

In adults with NAFLD, we discovered that most SGLT-2 inhibitors and GLP-1 receptor agonists included in our meta-analysis were more effective than placebo. According to the high certainty evidence of our network meta-analysis, GLP-1 receptor agonists seemed to be more effective than SGLT-2 inhibitors in reducing liver enzymes, liver fat, anthropometric measurements, and improving blood lipids and glycemic parameters.

In the absence of head-to-head trials, our network meta-analysis identified high confidence evidence for significant differences between these nine different drugs (5 SGLT-2 inhibitors and 4 GLP-1 receptor agonists). In terms of liver enzymes, semaglutide had relatively lower ALT, AST, and GGT levels than other included drugs. In terms of liver fat parameters, semaglutide had a lower LSM than the other included drugs (For SAT and VAT, exenatide appeared to function best, but the certainty is extremely low due to a high risk of bias and substantial indirectness, while for LFF and CAP, liraglutide and dapagliflozin seemed to work best respectively but the certainty is low owing to a high risk of bias and indirectness). In terms of anthropometric measures, semaglutide had a relatively lower BW than other included drugs (As for BMI and WC, exenatide seemed to work best but the certainty is low owing to a high risk of bias and indirectness, while for SBP, ipragliflozin seemed work best but the certainty is low owing to a high risk of bias and indirectness). In terms of blood lipids, high certainty evidence in which drug work best is lacking, semaglutide has a definite effect on decreasing TG and increasing HDL-C but not the best (As for TG, ipragliflozin seemed to work best but the certainty is moderate owing to a high risk of bias, while for HDL-C, dapagliflozin seemed to work best but the certainty is low owing to a high risk of bias and indirectness). In terms of glycemic parameters, liraglutide relatively reduced FBG and HOMA-IR than other included drugs, whereas dapagliflozin had a relatively lower PBG than other included drugs (As for HbA1c, luseogliflozin seemed work best but the certainty is extremely low due to a high risk of bias and substantial indirectness).

Of the five SGLT-2 inhibitors included, there seemed to be no significant difference in efficacy between dapagliflozin, ipragliflozin, luseogliflozin, tofogliflozin, and empagliflozin. Meanwhile, semaglutide appeared to have a greater effect than exenatide, liraglutide, and dulaglutide among the four GLP-1 receptor agonists included.

Despite finding positive effects, we were unable to identify drugs with substantial effects on DBP, TC, or LDL-C in both SGLT-2 inhibitors and GLP-1 receptor agonists. Additionally, we found some clinical uncertainty. Since the plasma concentration of adiponectin is negatively correlated with body weight, body mass index (BMI), central obesity, ectopic fat deposition and insulin resistance (IR) (Cnop et al., 2003; Ziemke and Mantzoros, 2010), it was considered a possible screening tools and therapeutic agents for NAFL-induced liver injury (Boutari and Mantzoros, 2020). In our NMAs, we found that ipragliflozin could significantly reduce adiponectin levels. Given the poor level of evidence and the limited number of studies included, the results need to be verified by more studies, and we should be cautious when using ipragliflozin for NAFLD.

### Comparisons with other studies

Mantovani et al. (2020a) previously demonstrated that SGLT-2 inhibitors dramatically enhance serum liver enzyme levels and liver fat content as measured by imaging techniques. Our findings are mostly consistent with these observations. Furthermore, Rezaei et al. (2021) conducted a recent meta-analysis on the impact of GLP-1 receptor agonists on liver enzymes and lipid profiles in NAFLD patients. Their findings demonstrated that GLP-1 receptor agonist therapy considerably decreases liver enzymes in patients with NAFLD, but not the lipid profile, which differs from our findings. A possible explanation for the disparity is that the included studies differed. Their meta-analysis included ten studies (seven studies used liraglutide and three studies used exenatide as an intervention), so their result only based on the effects of liraglutide and exenatide for NAFLD (Rezaei et al., 2021). As for our network meta-analysis, we included 4 different GLP-1 receptor agonists (semaglutide, liraglutide, exenatide and dulaglutide).

According to a recent network meta-analysis by Ding et al. (2022), SGLT-2 inhibitors and GLP-1 receptor agonists improved liver enzymes, BMI, blood lipid, blood glucose, and insulin resistance in NAFLD patients. But their study did not assess the effects of each drug of SGLT2 inhibitors and GLP-1 receptor agonists for NAFLD treatment. Our study expands on previous research in terms of the number of included studies (37 vs. 22), the number of included patients (about 3,172 vs. 1,351 patients), and the number of reported outcomes (22 vs. 10) (Ding et al., 2022). Our analysis is substantially more comprehensive because we assessed the effects of each drug of SGLT2 inhibitors and GLP-1 receptor agonists for NAFLD. Meanwhile, the significantly bigger evidence base gathered by the comprehensive search for published and unpublished data enabled us to analyze other significant outcomes, such as liver enzymes (ALT, AST, GGT), liver fat parameters (SAT, VAT, LFF, CAP, LSM), blood lipids (TC, TG, HDL-C, LDL-C, adiponectin), glycemic parameters (FBG, PBG, HbA1c, HOMA-IR) and anthropometric measures (BW, BMI, WC, SBP, DBP).

### Strengths and limitations

Current guidelines do not explicitly recommend specific drugs for the treatment of NAFLD, this NMAs saw the possible efficacy of SGLT-2 inhibitors and GLP-1 receptor agonists in NAFLD patients by summarizing all randomized controlled trial evidence. The added liver benefits of these two new classes of hypoglycemic agents provide options for the clinical practice of NAFLD patients, especially those with T2DM. Meanwhile, given the comparison of the effects of nine drugs on multiple indicators, this study provides detailed information for clinicians to evaluate the strengths and limitations of practice choice among numerous viable options.

To the best of our knowledge, this is the most comprehensive network meta-analysis on this topic. Unlike traditional pair-wise meta-analysis, our NMAs enable comparisons between SGLT-2 inhibitors and GLP-1 receptor agonists that have not been evaluated head-to-head in RCTs. Moreover, our NMA treats all comparators as independent treatments, improving statistical power by including all accessible data. We utilized the GRADE model to analyze the evidence certainty.

Our study has some limitations. Firstly, we did not have enough head-to-head investigations in our analysis, which indicates that the number of trials giving evidence for various comparisons in the network was insufficient, and the evidential certainty of certain outcomes may be diminished. Results should be interpreted with caution, especially if the confidence of the evidence is low or very low. Second, NAFLD assessment had two major components: inflammatory (liver enzymes) and structural (biopsy). Our NMAs only evaluated liver enzymes and several important Fibroscan indicators (CAP, LSM) as primary outcomes, and we were unable to quantify some liver structural outcomes due to a lack of biopsy information reported in the original studies. Therefore, this network meta-analysis could not evaluate the effects of SGLT-2 inhibitors and GLP-1 receptor agonists on the liver structure. Thirdly, most of the studies (about 70%) we included were on NAFLD with type 2 diabetes, and only a few were on NAFLD alone. We think this is acceptable given the close physiological relationship and frequent comorbidity between NAFLD and type 2 diabetes. In addition, adverse events of the included drugs were summarized and reviewed, but due to insufficient data, we did not perform a meta-analysis of adverse events or provide a classification of evidence for harm (or drug side-effects) of SGLT-2 inhibitors and GLP-1 receptor agonists in patients with NAFLD.

## Conclusion

In summary, this network meta-analysis provided evidence for the effects of SGLT-2 inhibitors and GLP-1 receptor agonists on NAFLD. Based on high confidence evidence of indirect comparisons, semaglutide, liraglutide and dapagliflozin all have a definite effect on NAFLD (or comorbid with type 2 diabetes) and semaglutide appears to have a therapeutic advantage over other included drugs. The effects of included medication therapy on various indicators differ, and clinical selection should be based on the specific condition of the patient. Although the majority of the SGLT-2 inhibitors and GLP-1 receptor agonists included in this analysis were effective for the treatment of NAFLD, there was no sufficient evidence to evaluate the impact of SGLT-2 inhibitors and GLP-1 receptor agonists on the liver structure. Head-to-head studies are required to provide more confidence in clinical decision-making.

## Data Availability

The original contributions presented in the study are included in the article/Supplementary Material, further inquiries can be directed to the corresponding authors.
