# Supplementary material for: Comparative efficacy of 5 sodium-glucose cotransporter protein-2 (SGLT-2) inhibitor and 4 glucagon-like peptide-1 (GLP-1) receptor agonist drugs in non-alcoholic fatty liver disease: A GRADE-assessed systematic review and network meta-analysis of randomized controlled trials
Source: Front Pharmacol. 2023 Mar 13;14:1102792. doi: 10.3389/fphar.2023.1102792 (PMC10040540; doi:10.3389/fphar.2023.1102792)
Supplement: Supplementary file 1 [file DataSheet1.PDF]

Comparative efficacy of 5 sodium-glucose cotransporter protein-2 (SGLT-2) inhibitor and 4 glucagon-like peptide-1 (GLP-1) receptor agonist drugs in nonalcoholic fatty liver disease: GRADE-assessed systematic review and network meta-analysis

Supplementary Material

Table of contents **Page**

Appendix 1 Protocol 2
Appendix 2 Search strategies 7
Appendix 3 Included studies 17
Appendix 4 Reference list for included studies 24
Appendix 5 Risk of bias in included studies 28
Appendix 6 Network plots for each outcome 29
Appendix 7 Evaluations of network inconsistency 41
Appendix 8 Direct, indirect and network treatment estimates 42
Appendix 9 Network meta-analysis treatment estimates 64
Appendix 10 Funnel plots for each outcome 86
Appendix 11 GRADE summary of findings for SGLT-2 inhibitors and GLP-1 receptor agonists compared to placebo or each other 97

Appendix 1 Protocol

Sodium-glucose cotransporter protein-2 (SGLT-2) inhibitors and glucagon-like peptide-1 (GLP-1) receptor agonists for non-alcoholic fatty liver disease: a protocol for a network meta-analysis of randomized controlled trials

Correspondence to

Junping Shi

20131004@hznu.edu.cn

Abstract

Objective: To compare the effects of Sodium-glucose cotransporter protein-2 (SGLT-2) inhibitors and glucagon-like peptide-1 (GLP-1) receptor agonists for non-alcoholic fatty liver disease

Design: Systematic review and network meta-analysis (NMA).

Data sources: Electronic databases (PubMed, Embase, Web of Science, and Cochrane Library).

Study selection: We will include randomized clinical trials (RCT) in which patients were diagnosed with non-alcoholic fatty liver disease (NAFLD) and treated with SGLT-2 inhibitors or GLP-1 receptor agonists. Pairs of independent reviewers will screen in duplicate title and abstract and full text of potentially eligible articles.

Methods: After duplicate data abstraction, we will conduct a frequentist pairwise meta-analysis for each of the outcomes of interest. We will assess the risk of bias of the included studies using a modification of the Cochrane Risk of Bias tool, and the certainty of the evidence using the GRADE approach for NMA.

Ethical issues: Ethical approval and patient consent are not required since this is a network meta-analysis based on published studies.

Publication: We will publish the results in a traditional format for systematic reviews and network meta-analysis.

Introduction

NAFLD has become the primary cause of current chronic liver disease, with a high incidence rate of up to 25% worldwide.1 The increased prevalence of NAFLD worldwide is particularly worrisome as no medication has been approved to treat NAFLD yet. Lifestyle modification, especially dietary interventions, remains the first-line approach for treating NAFLD currently.2 It is known that type 2 diabetes is one of the strongest clinical risk factors for a faster progression of NAFLD to NASH, cirrhosis, and hepatocellular carcinoma,3-6 some newly antidiabetic drugs were found to be beneficial in NAFLD such as sodium-glucose cotransporter protein-2 (SGLT-2) and glucagon-like peptide-1 (GLP-1). SGLT-2 acts by helping in renal excretion of glucose and, therefore, will cause a reduction of body weight (on average 2.5–3.0 kg) and prevalence of obesity that may improve

the liver histology of those with NAFLD/NASH.7 GLP-1 are a newly introduced class of antidiabetic drugs that improve glycemic control via several molecular pathways.

The relative effectiveness of GLP-1 and SGLT-2 for NAFLD treatments is difficult to discern from the literature, in part because few head-to-head comparison studies are available and traditional pairwise meta-analysis cannot integrate all of the evidence from several competitors. However, to date, no network meta-analysis has been published on the relative effectiveness of GLP-1 and SGLT-2 for NAFLD. A network meta-analysis of NAFLD was published in 2021. Therefore, our goal is to comprehensively review the literature and determine the relative efficacy of each specific drugs for NAFLD, which include the changes in liver enzymes, blood lipids and glycemic parameters, as well as changes in body weight and liver fat, while also to examines the evidence as to whether any drug is better than others.

Methods

Design

We will conduct a systematic review and network meta-analysis.

Eligibility criteria

Studies will include in the systematic review and network meta-analysis if they meet the following criteria: 1) enrolled individuals with NAFLD, in which the diagnosis of this liver disease was based on ultrasonography or liver biopsy or magnetic resonance-based techniques; 2) they were active-controlled or placebo-controlled randomized controlled trials that used GLP-1R agonists, or SGLT2 inhibitors for the treatment of NAFLD; Studies will exclude if they are: 1) observational or non-randomized intervention studies; 2) trials enrolling children or adolescents (younger than 18 years old).

Data sources and searches

We will search four large electronic databases (PubMed, Embase, Web of Science, and Cochrane Library), using predefined keywords to identify relevant active-controlled or placebo-controlled randomized trials of adults with NAFLD. Searches will restrict to human studies. Studies in languages other than English will also exclude. Two independent investigators will review the titles and abstracts of all citations identified by the search.

Study selection

Full-text articles will retrieve for the included abstracts and will subsequently screen for eligibility (according to the aforementioned inclusion criteria) by two independent investigators. Disagreements at this level will resolve by consensus and a third reviewer if needed.

Data extraction

Data extraction will be individually by two investigators. For all studies, we will extract information on first author, publication year, study country, number of participants, main participants' characteristics, types of interventions (and daily dosages of drugs

used), duration of treatment, methods used for the diagnosis of NAFLD, and outcomes.

Outcomes

We will begin by focusing on the patient-important outcomes listed below. We will consider liver enzymes and liver fat parameters as primary outcomes.

- alanine aminotransferase (ALT)
- aspartate aminotransferase (AST)
- γ-glutamyl transferase (GGT)]
- subcutaneous adipose tissue (SAT)
- visceral adipose tissue (VAT)
- liver fat fraction (LFF)
- controlled attenuation parameter (CAP)
- liver stiffness measurement (LSM)]

We will consider anthropometric measures, blood lipids and glycemic parameters as primary outcomes.

- body weight (BW)
- body mass index (BMI)
- waist circumference (WC)
- systobic blood pressure (SBP)
- diastolic blood pressure (DBP)
- [total cholesterol(TC)
- triglycerides (TG)
- high density lipoprotein-cholesterol (HDL-C)
- low density lipoprotein-cholesterol (LDL-C)
- adiponectin
- fasting blood.glucose (FBG)
- postprandial blood glucose (PBG)
- glycosylated hemoglobin (HbA1c)
- glucose and homeostasis model assessment (HOMA-IR)].

Risk of bias assessments

Each eligible study will assess for quality by assessing the risk of bias by two independent reviewers, with disagreements will resolve through con-sensus. The risk of bias for each study will assess using the Cochrane risk of bias tool, by which studies will deem to be at low, high, or unclear risk.[8]

Data synthesis

For each direct comparison of two treatments, we will conduct a frequentist pairwise meta-analysis using a restricted maximum likelihood estimation and reported, with corresponding 95% confidence intervals, mean differences for continuous outcomes.[9] We will assess statistical heterogeneity using the I^2 statistic and funnel plots for evidence of small study effects in analyses including 10 or more

studies. A single estimate will obtain for all outcomes and treatment comparisons, which will be used in combination with the baseline risk estimates to present results as absolute effects. All pairwise and network meta-analyses will perform with Stata 15 (StataCorp, College Station, TX) using published routines.[10]

Subgroups and sensitivity analyses

We will plan subgroup analysis if sufficient data are available

Certainty assessment

We will conduct network meta-analysis using frequentist methods with restricted maximum likelihood estimation to quantify network heterogeneity, assuming a common heterogeneity estimate within a network. We will assess agreement between direct and indirect estimates in every closed loop of evidence using node splitting approaches, and for the entire network using a design-by-treatment interaction model.[11] We will impute missing standard deviations for continuous variables when absent using standard deviations borrowed from other similar randomized controlled trials.[12 13]

Ethical issues

Ethical approval and patient consent are not required since this is a network meta-analysis based on published studies. Publication.

Publication

The papers will be published in a traditional format for systematic reviews and network meta-analyses.

Acknowledgments

We are grateful for the helpful reviewer comments on this paper.

Reference

1. Younossi ZM, Koenig AB, Abdelatif D, et al. Global epidemiology of nonalcoholic fatty liver disease-Meta-analytic assessment of prevalence, incidence, and outcomes. *Hepatology* 2016;64(1):73-84. doi: 10.1002/hep.28431 [published Online First: 2015/12/29]

2. El-Agroudy NN, Kurzbach A, Rodionov RN, et al. Are Lifestyle Therapies Effective for NAFLD Treatment? *Trends in endocrinology and metabolism: TEM* 2019;30(10):701-09. doi: 10.1016/j.tem.2019.07.013 [published Online First: 2019/08/20]

3. Younossi Z, Anstee QM, Marietti M, et al. Global burden of NAFLD and NASH: trends, predictions, risk factors and prevention. *Nature reviews Gastroenterology & hepatology* 2018;15(1):11-20. doi: 10.1038/nrgastro.2017.109 [published Online First: 2017/09/21]

4. Targher G, Lonardo A, Byrne CD. Nonalcoholic fatty liver disease and chronic vascular complications of diabetes mellitus. *Nature reviews Endocrinology* 2018;14(2):99-114. doi: 10.1038/nrendo.2017.173 [published Online First: 2017/12/30]

5. Mantovani A, Scorletti E, Mosca A, et al. Complications, morbidity and mortality of nonalcoholic fatty liver disease. *Metabolism* 2020;111s:154170. doi: 10.1016/j.metabol.2020.154170 [published

Online First: 2020/02/02]

6. Targher G, Corey KE, Byrne CD, et al. The complex link between NAFLD and type 2 diabetes mellitus - mechanisms and treatments. *Nature reviews Gastroenterology & hepatology* 2021;18(9):599-612. doi: 10.1038/s41575-021-00448-y [published Online First: 2021/05/12]
7. Brunton SA. The potential role of sodium glucose co-transporter 2 inhibitors in the early treatment of type 2 diabetes mellitus. *Int J Clin Pract* 2015;69(10):1071-87. doi: 10.1111/ijcp.12675 [published Online First: 2015/07/07]
8. Higgins JP, Altman DG, Gøtzsche PC, et al. The Cochrane Collaboration's tool for assessing risk of bias in randomised trials. *BMJ (Clinical research ed)* 2011;343:d5928. doi: 10.1136/bmj.d5928 [published Online First: 2011/10/20]
9. Liu Y, Wang W, Zhang AB, et al. Epley and Semont maneuvers for posterior canal benign paroxysmal positional vertigo: A network meta-analysis. *The Laryngoscope* 2016;126(4):951-5. doi: 10.1002/lary.25688 [published Online First: 2015/09/26]
10. Chaimani A, Higgins JP, Mavridis D, et al. Graphical tools for network meta-analysis in STATA. *PLoS One* 2013;8(10):e76654. doi: 10.1371/journal.pone.0076654 [published Online First: 2013/10/08]
11. Veroniki AA, Vasiliadis HS, Higgins JP, et al. Evaluation of inconsistency in networks of interventions. *International journal of epidemiology* 2013;42(1):332-45. doi: 10.1093/ije/dys222 [published Online First: 2013/03/20]
12. Dias S, Welton NJ, Caldwell DM, et al. Checking consistency in mixed treatment comparison meta-analysis. *Statistics in medicine* 2010;29(7-8):932-44. doi: 10.1002/sim.3767 [published Online First: 2010/03/10]
13. Furukawa TA, Barbui C, Cipriani A, et al. Imputing missing standard deviations in meta-analyses can provide accurate results. *Journal of clinical epidemiology* 2006;59(1):7-10. doi: 10.1016/j.jclinepi.2005.06.006 [published Online First: 2005/12/20]

Appendix 2 Search strategies

1. Canagliflozin

PubMed

(("Non-alcoholic Fatty Liver Disease"[Mesh]) OR (((((((((((((Non-alcoholic Fatty Liver Disease[Title/Abstract]) OR (Non alcoholic Fatty Liver Disease[Title/Abstract])) OR (NAFLD[Title/Abstract])) OR (Nonalcoholic Fatty Liver Disease[Title/Abstract])) OR (Fatty Liver, Nonalcoholic[Title/Abstract])) OR (Fatty Livers, Nonalcoholic[Title/Abstract])) OR (Liver, Nonalcoholic Fatty[Title/Abstract])) OR (Livers, Nonalcoholic Fatty[Title/Abstract])) OR (Nonalcoholic Fatty Liver[Title/Abstract])) OR (Nonalcoholic Fatty Livers[Title/Abstract])) OR (Nonalcoholic Steatohepatitis[Title/Abstract])) OR (Nonalcoholic Steatohepatitides[Title/Abstract])) OR (Steatohepatitides, Nonalcoholic[Title/Abstract])) OR (Steatohepatitis, Nonalcoholic[Title/Abstract]))) AND (("Canagliflozin"[Mesh]) OR (((((Canagliflozin[Title/Abstract]) OR (Invokana[Title/Abstract])) OR (Canagliflozin Hemihydrate[Title/Abstract])) OR (Canagliflozin, Anhydrous[Title/Abstract])) OR (1-(Glucopyranosyl)-4-methyl-3-(5-(4-fluorophenyl)-2-thienylmethyl)benzene-T777973[Title/Abstract])))

Web of science

TS= (Non-alcoholic Fatty Liver Disease OR Non alcoholic Fatty Liver Disease OR NAFLD OR Nonalcoholic Fatty Liver Disease OR Fatty Liver, Nonalcoholic OR Fatty Livers, Nonalcoholic OR Liver, Nonalcoholic Fatty OR Livers, Nonalcoholic Fatty OR Nonalcoholic Fatty Liver OR Nonalcoholic Fatty Livers OR Nonalcoholic Steatohepatitis OR Nonalcoholic Steatohepatitides OR Steatohepatitides, Nonalcoholic OR Steatohepatitis, Nonalcoholic) AND TS= （Canagliflozin OR Invokana OR Canagliflozin Hemihydrate OR Canagliflozin, Anhydrous OR 1-(Glucopyranosyl)-4-methyl-3-(5-(4-fluorophenyl)-2-thienylmethyl)benzene-T777973）

Embase

#3 #1 AND #2

#2 Canagliflozin:ab,ti OR Invokana:ab,ti OR 'Canagliflozin Hemihydrate':ab,ti OR 'Canagliflozin, Anhydrous':ab,ti OR '1-(Glucopyranosyl)-4-methyl-3-(5-(4-fluorophenyl)-2-thienylmethyl)benzene-T777973':ab,ti

#1 'Non-alcoholic Fatty Liver Disease':ab,ti OR 'Non alcoholic Fatty Liver Disease':ab,ti OR NAFLD:ab,ti OR 'Nonalcoholic Fatty Liver Disease':ab,ti OR 'Fatty Liver, Nonalcoholic':ab,ti OR 'Fatty Livers, Nonalcoholic':ab,ti OR 'Liver, Nonalcoholic Fatty':ab,ti OR 'Livers, Nonalcoholic Fatty':ab,ti OR 'Nonalcoholic Fatty Liver':ab,ti OR 'Nonalcoholic Fatty Livers':ab,ti OR 'Nonalcoholic Steatohepatitis':ab,ti OR 'Nonalcoholic Steatohepatitides':ab,ti OR 'Steatohepatitides, Nonalcoholic':ab,ti OR 'Steatohepatitis, Nonalcoholic':ab,ti

Cochrane library

#3 #1 AND #2

#2 (Canagliflozin OR Invokana OR Canagliflozin Hemihydrate OR Canagliflozin, Anhydrous):ti,ab,kw

#1 (Non-alcoholic Fatty Liver Disease OR Non alcoholic Fatty Liver Disease OR NAFLD OR Nonalcoholic Fatty Liver Disease OR Fatty Liver, Nonalcoholic OR Fatty Livers, Nonalcoholic OR Liver, Nonalcoholic Fatty OR Livers, Nonalcoholic Fatty OR Nonalcoholic Fatty Liver OR Nonalcoholic Fatty Livers OR Nonalcoholic Steatohepatitis OR Nonalcoholic Steatohepatitides OR Steatohepatitides, Nonalcoholic OR Steatohepatitis, Nonalcoholic):ti,ab,kw

2. Dapagliflozin

PubMed

(("Non-alcoholic Fatty Liver Disease"[Mesh]) OR (((((((((((((Non-alcoholic Fatty Liver Disease[Title/Abstract]) OR (Non alcoholic Fatty Liver Disease[Title/Abstract])) OR (NAFLD[Title/Abstract])) OR (Nonalcoholic Fatty Liver Disease[Title/Abstract])) OR (Fatty Liver, Nonalcoholic[Title/Abstract])) OR (Fatty Livers, Nonalcoholic[Title/Abstract])) OR (Liver, Nonalcoholic Fatty[Title/Abstract])) OR (Livers, Nonalcoholic Fatty[Title/Abstract])) OR (Nonalcoholic Fatty Liver[Title/Abstract])) OR (Nonalcoholic Fatty Livers[Title/Abstract])) OR (Nonalcoholic Steatohepatitis[Title/Abstract])) OR (Nonalcoholic Steatohepatitides[Title/Abstract])) OR (Steatohepatitides, Nonalcoholic[Title/Abstract])) OR (Steatohepatitis, Nonalcoholic[Title/Abstract]))) AND (("dapagliflozin"[Supplementary Concept]) OR ((((((((dapagliflozin[Title/Abstract]) OR ((2S,3R,4R,5S,6R)-2-(4-chloro-3-(4-ethoxybenzyl)phenyl)-6- (hydroxymethyl)tetrahydro-2H-pyran-3,4,5-triol[Title/Abstract])) OR (Farxiga[Title/Abstract])) OR (Forxiga[Title/Abstract])) OR (2-(3-(4-ethoxybenzyl)-4-chlorophenyl)-6-hydroxymethyltetrahydro-2H-pyran-3,4,5-triol[Title/Abstract])) OR (BMS 512148[Title/Abstract])) OR (BMS512148[Title/Abstract])) OR (BMS-512148[Title/Abstract])))

Web of science

TS= (Non-alcoholic Fatty Liver Disease OR Non alcoholic Fatty Liver Disease OR NAFLD OR Nonalcoholic Fatty Liver Disease OR Fatty Liver, Nonalcoholic OR Fatty Livers, Nonalcoholic OR Liver, Nonalcoholic Fatty OR Livers, Nonalcoholic Fatty OR Nonalcoholic Fatty Liver OR Nonalcoholic Fatty Livers OR Nonalcoholic Steatohepatitis OR Nonalcoholic Steatohepatitides OR Steatohepatitides, Nonalcoholic OR Steatohepatitis, Nonalcoholic) AND TS= （dapagliflozin OR (2S,3R,4R,5S,6R)-2-(4-chloro-3-(4-ethoxybenzyl)phenyl)-6- (hydroxymethyl)tetrahydro-2H-pyran-3,4,5-triol OR Farxiga OR Forxiga OR 2-(3-(4-ethoxybenzyl)-4-chlorophenyl)-6-hydroxymethyltetrahydro-2H-pyran-3,4,5-triol OR BMS 512148 OR BMS512148 OR BMS-512148）

Embase

#3 #1 AND #2

#2 dapagliflozin:ab,ti OR '(2S,3R,4R,5S,6R)-2-(4-chloro-3-(4-ethoxybenzyl)phenyl)-6-(hydroxymethyl)tetrahydro-2H-pyran-3,4,5-triol':ab,ti OR Farxiga:ab,ti OR Forxiga:ab,ti OR '2-(3-(4-ethoxybenzyl)-4-chlorophenyl)-6-hydroxymethyltetrahydro-2H-pyran-3,4,5-triol':ab,ti OR 'BMS 512148':ab,ti OR 'BMS512148':ab,ti OR 'BMS-512148':ab,ti

#1 'Non-alcoholic Fatty Liver Disease':ab,ti OR 'Non alcoholic Fatty Liver Disease':ab,ti OR NAFLD:ab,ti OR 'Nonalcoholic Fatty Liver Disease':ab,ti OR 'Fatty Liver, Nonalcoholic':ab,ti OR 'Fatty Livers, Nonalcoholic':ab,ti OR 'Liver, Nonalcoholic Fatty':ab,ti OR 'Livers, Nonalcoholic Fatty':ab,ti OR 'Nonalcoholic Fatty Liver':ab,ti OR 'Nonalcoholic Fatty Livers':ab,ti OR 'Nonalcoholic Steatohepatitis':ab,ti OR 'Nonalcoholic Steatohepatitides':ab,ti OR 'Steatohepatitides, Nonalcoholic':ab,ti OR 'Steatohepatitis, Nonalcoholic':ab,ti

Cochrane library

#3 #1 AND #2

#2 (dapagliflozin OR (2S,3R,4R,5S,6R)-2-(4-chloro-3-(4-ethoxybenzyl)phenyl)-6-(hydroxymethyl)tetrahydro-2H-pyran-3,4,5-triol OR Farxiga OR Forxiga OR 2-(3-(4-ethoxybenzyl)-4-chlorophenyl)-6-hydroxymethyltetrahydro-2H-pyran-3,4,5-triol OR BMS 512148 OR BMS512148 OR BMS-512148):ti,ab,kw

#1 (Non-alcoholic Fatty Liver Disease OR Non alcoholic Fatty Liver Disease OR NAFLD OR Nonalcoholic Fatty Liver Disease OR Fatty Liver, Nonalcoholic OR Fatty Livers, Nonalcoholic OR Liver, Nonalcoholic Fatty OR Livers, Nonalcoholic Fatty OR Nonalcoholic Fatty Liver OR Nonalcoholic Fatty Livers OR Nonalcoholic Steatohepatitis OR Nonalcoholic Steatohepatitides OR Steatohepatitides, Nonalcoholic OR Steatohepatitis, Nonalcoholic):ti,ab,kw

3. Ipragliflozin

PubMed

(("Non-alcoholic Fatty Liver Disease"[Mesh]) OR (((((((((((((Non-alcoholic Fatty Liver Disease[Title/Abstract]) OR (Non alcoholic Fatty Liver Disease[Title/Abstract])) OR (NAFLD[Title/Abstract])) OR (Nonalcoholic Fatty Liver Disease[Title/Abstract])) OR (Fatty Liver, Nonalcoholic[Title/Abstract])) OR (Fatty Livers, Nonalcoholic[Title/Abstract])) OR (Liver, Nonalcoholic Fatty[Title/Abstract])) OR (Livers, Nonalcoholic Fatty[Title/Abstract])) OR (Nonalcoholic Fatty Liver[Title/Abstract])) OR (Nonalcoholic Fatty Livers[Title/Abstract])) OR (Nonalcoholic Steatohepatitis[Title/Abstract])) OR (Nonalcoholic Steatohepatitides[Title/Abstract])) OR (Steatohepatitides, Nonalcoholic[Title/Abstract])) OR (Steatohepatitis, Nonalcoholic[Title/Abstract]))) AND (("Ipragliflozin"[Supplementary Concept]) OR (((((Ipragliflozin[Title/Abstract]) OR ((1S)-1,5-anhydro-1-(3-(1-benzothiophen-2-ylmethyl)-4-fluorophenyl)-D-glucitol [Title/Abstract])) OR (Suglat[Title/Abstract])) OR (ASP1941 [Title/Abstract])) OR (ASP-1941[Title/Abstract])))

Web of science

TS= (Non-alcoholic Fatty Liver Disease OR Non alcoholic Fatty Liver Disease OR NAFLD OR Nonalcoholic Fatty Liver Disease OR Fatty Liver, Nonalcoholic OR Fatty Livers, Nonalcoholic OR Liver, Nonalcoholic Fatty OR Livers, Nonalcoholic Fatty OR Nonalcoholic Fatty Liver OR Nonalcoholic Fatty Livers OR Nonalcoholic Steatohepatitis OR Nonalcoholic Steatohepatitides OR Steatohepatitides, Nonalcoholic OR Steatohepatitis, Nonalcoholic) AND TS= （Ipragliflozin OR (1S)-1,5-anhydro-1-(3-(1-benzothiophen-2-ylmethyl)-4-fluorophenyl)-D-glucitol OR Suglat OR ASP1941 OR ASP-1941）

Embase

#3 #1 AND #2

#2 Ipragliflozin:ab,ti OR '(1S)-1,5-anhydro-1-(3-(1-benzothiophen-2-ylmethyl)-4-fluorophenyl)-D-glucitol':ab,ti OR Suglat:ab,ti OR ASP1941:ab,ti OR ASP-1941:ab,ti

#1 'Non-alcoholic Fatty Liver Disease':ab,ti OR 'Non alcoholic Fatty Liver Disease':ab,ti OR NAFLD:ab,ti OR 'Nonalcoholic Fatty Liver Disease':ab,ti OR 'Fatty Liver, Nonalcoholic':ab,ti OR 'Fatty Livers, Nonalcoholic':ab,ti OR 'Liver, Nonalcoholic Fatty':ab,ti OR 'Livers, Nonalcoholic Fatty':ab,ti OR 'Nonalcoholic Fatty Liver':ab,ti OR 'Nonalcoholic Fatty Livers':ab,ti OR 'Nonalcoholic Steatohepatitis':ab,ti OR 'Nonalcoholic Steatohepatitides':ab,ti OR 'Steatohepatitides, Nonalcoholic':ab,ti OR 'Steatohepatitis, Nonalcoholic':ab,ti

Cochrane library

#3 #1 AND #2

#2 (Ipragliflozin OR (1S)-1,5-anhydro-1-(3-(1-benzothiophen-2-ylmethyl)-4-fluorophenyl)-D-glucitol OR Suglat OR ASP1941 OR ASP-1941):ti,ab,kw

#1 (Non-alcoholic Fatty Liver Disease OR Non alcoholic Fatty Liver Disease OR NAFLD OR Nonalcoholic Fatty Liver Disease OR Fatty Liver, Nonalcoholic OR Fatty Livers, Nonalcoholic OR

Liver, Nonalcoholic Fatty OR Livers, Nonalcoholic Fatty OR Nonalcoholic Fatty Liver OR Nonalcoholic Fatty Livers OR Nonalcoholic Steatohepatitis OR Nonalcoholic Steatohepatitides OR Steatohepatitides, Nonalcoholic OR Steatohepatitis, Nonalcoholic):ti,ab,kw

4. Luseogliflozin

PubMed

(("Non-alcoholic Fatty Liver Disease"[Mesh]) OR (((((((((((((Non-alcoholic Fatty Liver Disease[Title/Abstract]) OR (Non alcoholic Fatty Liver Disease[Title/Abstract])) OR (NAFLD[Title/Abstract])) OR (Nonalcoholic Fatty Liver Disease[Title/Abstract])) OR (Fatty Liver, Nonalcoholic[Title/Abstract])) OR (Fatty Livers, Nonalcoholic[Title/Abstract])) OR (Liver, Nonalcoholic Fatty[Title/Abstract])) OR (Livers, Nonalcoholic Fatty[Title/Abstract])) OR (Nonalcoholic Fatty Liver[Title/Abstract])) OR (Nonalcoholic Fatty Livers[Title/Abstract])) OR (Nonalcoholic Steatohepatitis[Title/Abstract])) OR (Nonalcoholic Steatohepatitides[Title/Abstract])) OR (Steatohepatitides, Nonalcoholic[Title/Abstract])) OR (Steatohepatitis, Nonalcoholic[Title/Abstract]))) AND (("1,5-anhydro-1-(5-(4-ethoxybenzyl)-2-methoxy-4-methylphenyl)-1-thioglucitol"[Supplementary Concept]) OR ((((((1,5-anhydro-1-(5-(4-ethoxybenzyl)-2-methoxy-4-methylphenyl)-1-thioglucitol[Title/Abstract]) OR (luseogliflozin[Title/Abstract])) OR (Lusefi[Title/Abstract])) OR (TS 071[Title/Abstract])) OR (TS071 cpd[Title/Abstract])) OR (TS-071[Title/Abstract])))

Web of science

TS= (Non-alcoholic Fatty Liver Disease OR Non alcoholic Fatty Liver Disease OR NAFLD OR Nonalcoholic Fatty Liver Disease OR Fatty Liver, Nonalcoholic OR Fatty Livers, Nonalcoholic OR Liver, Nonalcoholic Fatty OR Livers, Nonalcoholic Fatty OR Nonalcoholic Fatty Liver OR Nonalcoholic Fatty Livers OR Nonalcoholic Steatohepatitis OR Nonalcoholic Steatohepatitides OR Steatohepatitides, Nonalcoholic OR Steatohepatitis, Nonalcoholic) AND TS= （1,5-anhydro-1-(5-(4-ethoxybenzyl)-2-methoxy-4-methylphenyl)-1-thioglucitol OR luseogliflozin OR Lusefi OR TS 071 OR TS071 cpd OR TS-071）

Embase

#3 #1 AND #2

#2 '1,5-anhydro-1-(5-(4-ethoxybenzyl)-2-methoxy-4-methylphenyl)-1-thioglucitol':ab,ti OR luseogliflozin:ab,ti OR Lusefi:ab,ti OR TS 071:ab,ti OR TS071 cpd:ab,ti OR TS-071:ab,ti

#1 'Non-alcoholic Fatty Liver Disease':ab,ti OR 'Non alcoholic Fatty Liver Disease':ab,ti OR NAFLD:ab,ti OR 'Nonalcoholic Fatty Liver Disease':ab,ti OR 'Fatty Liver, Nonalcoholic':ab,ti OR 'Fatty Livers, Nonalcoholic':ab,ti OR 'Liver, Nonalcoholic Fatty':ab,ti OR 'Livers, Nonalcoholic Fatty':ab,ti OR 'Nonalcoholic Fatty Liver':ab,ti OR 'Nonalcoholic Fatty Livers':ab,ti OR 'Nonalcoholic Steatohepatitis':ab,ti OR 'Nonalcoholic Steatohepatitides':ab,ti OR 'Steatohepatitides, Nonalcoholic':ab,ti OR 'Steatohepatitis, Nonalcoholic':ab,ti

Cochrane library

#3 #1 AND #2

#2 (1,5-anhydro-1-(5-(4-ethoxybenzyl)-2-methoxy-4-methylphenyl)-1-thioglucitol OR luseogliflozin OR Lusefi OR TS 071 OR TS071 cpd OR TS-071):ti,ab,kw

#1 (Non-alcoholic Fatty Liver Disease OR Non alcoholic Fatty Liver Disease OR NAFLD OR Nonalcoholic Fatty Liver Disease OR Fatty Liver, Nonalcoholic OR Fatty Livers, Nonalcoholic OR Liver, Nonalcoholic Fatty OR Livers, Nonalcoholic Fatty OR Nonalcoholic Fatty Liver OR

Nonalcoholic Fatty Livers OR Nonalcoholic Steatohepatitis OR Nonalcoholic Steatohepatitides OR Steatohepatitides, Nonalcoholic OR Steatohepatitis, Nonalcoholic):ti,ab,kw

5. Tofogliflozin

PubMed

(("Non-alcoholic Fatty Liver Disease"[Mesh]) OR (((((((((((((Non-alcoholic Fatty Liver Disease[Title/Abstract]) OR (Non alcoholic Fatty Liver Disease[Title/Abstract])) OR (NAFLD[Title/Abstract])) OR (Nonalcoholic Fatty Liver Disease[Title/Abstract])) OR (Fatty Liver, Nonalcoholic[Title/Abstract])) OR (Fatty Livers, Nonalcoholic[Title/Abstract])) OR (Liver, Nonalcoholic Fatty[Title/Abstract])) OR (Livers, Nonalcoholic Fatty[Title/Abstract])) OR (Nonalcoholic Fatty Liver[Title/Abstract])) OR (Nonalcoholic Fatty Livers[Title/Abstract])) OR (Nonalcoholic Steatohepatitis[Title/Abstract])) OR (Nonalcoholic Steatohepatitides[Title/Abstract])) OR (Steatohepatitides, Nonalcoholic[Title/Abstract])) OR (Steatohepatitis, Nonalcoholic[Title/Abstract]))) AND (("6-((4-ethylphenyl)methyl)-3',4',5',6'-tetrahydro-6'-(hydroxymethyl)spiro(isobenzofuran-1(3H),2'-(2H)pyran)-3',4',5'-triol"[Supplementary Concept]) OR ((((((6-((4-ethylphenyl)methyl)-3',4',5',6'-tetrahydro-6'-(hydroxymethyl)spiro(isobenzofuran-1(3H),2'-(2H)pyran)-3',4',5'-triol[Title/Abstract]) OR (tofogliflozin hydrate[Title/Abstract])) OR (CSG452[Title/Abstract])) OR (tofogliflozin anhydrous[Title/Abstract])) OR (Apleway[Title/Abstract])) OR (Deberza[Title/Abstract])) OR (tofogliflozin[Title/Abstract])))

Web of science

TS= (Non-alcoholic Fatty Liver Disease OR Non alcoholic Fatty Liver Disease OR NAFLD OR Nonalcoholic Fatty Liver Disease OR Fatty Liver, Nonalcoholic OR Fatty Livers, Nonalcoholic OR Liver, Nonalcoholic Fatty OR Livers, Nonalcoholic Fatty OR Nonalcoholic Fatty Liver OR Nonalcoholic Fatty Livers OR Nonalcoholic Steatohepatitis OR Nonalcoholic Steatohepatitides OR Steatohepatitides, Nonalcoholic OR Steatohepatitis, Nonalcoholic) AND TS= （6-((4-ethylphenyl)methyl)-3',4',5',6'-tetrahydro-6'-(hydroxymethyl)spiro(isobenzofuran-1(3H),2'-(2H)pyran)-3',4',5'-triol OR tofogliflozin hydrate OR CSG452 OR tofogliflozin anhydrous OR Apleway OR Deberza OR tofogliflozin）

Embase

#3 #1 AND #2

#2 '6-((4-ethylphenyl)methyl)-3',4',5',6'-tetrahydro-6'-(hydroxymethyl)spiro(isobenzofuran-1(3H),2'-(2H)pyran)-3',4',5'-triol':ab,ti OR 'tofogliflozin hydrate':ab,ti OR CSG452:ab,ti OR Apleway:ab,ti OR 'tofogliflozin anhydrous':ab,ti OR Deberza:ab,ti OR tofogliflozin:ab,ti

#1 'Non-alcoholic Fatty Liver Disease':ab,ti OR 'Non alcoholic Fatty Liver Disease':ab,ti OR NAFLD:ab,ti OR 'Nonalcoholic Fatty Liver Disease':ab,ti OR 'Fatty Liver, Nonalcoholic':ab,ti OR 'Fatty Livers, Nonalcoholic':ab,ti OR 'Liver, Nonalcoholic Fatty':ab,ti OR 'Livers, Nonalcoholic Fatty':ab,ti OR 'Nonalcoholic Fatty Liver':ab,ti OR 'Nonalcoholic Fatty Livers':ab,ti OR 'Nonalcoholic Steatohepatitis':ab,ti OR 'Nonalcoholic Steatohepatitides':ab,ti OR 'Steatohepatitides, Nonalcoholic':ab,ti OR 'Steatohepatitis, Nonalcoholic':ab,ti

Cochrane library

#3 #1 AND #2

#2 (6-((4-ethylphenyl)methyl)-3',4',5',6'-tetrahydro-6'-(hydroxymethyl)spiro(isobenzofuran-1(3H),2'-(2H)pyran)-3',4',5'-triol OR tofogliflozin hydrate OR CSG452 OR tofogliflozin anhydrous

OR Apleway OR Deberza OR tofogliflozin):ti,ab,kw
#1 (Non-alcoholic Fatty Liver Disease OR Non alcoholic Fatty Liver Disease OR NAFLD OR Nonalcoholic Fatty Liver Disease OR Fatty Liver, Nonalcoholic OR Fatty Livers, Nonalcoholic OR Liver, Nonalcoholic Fatty OR Livers, Nonalcoholic Fatty OR Nonalcoholic Fatty Liver OR Nonalcoholic Fatty Livers OR Nonalcoholic Steatohepatitis OR Nonalcoholic Steatohepatitides OR Steatohepatitides, Nonalcoholic OR Steatohepatitis, Nonalcoholic):ti,ab,kw

6. Empagliflozin

PubMed

(("Non-alcoholic Fatty Liver Disease"[Mesh]) OR (((((((((((((Non-alcoholic Fatty Liver Disease[Title/Abstract]) OR (Non alcoholic Fatty Liver Disease[Title/Abstract])) OR (NAFLD[Title/Abstract])) OR (Nonalcoholic Fatty Liver Disease[Title/Abstract])) OR (Fatty Liver, Nonalcoholic[Title/Abstract])) OR (Fatty Livers, Nonalcoholic[Title/Abstract])) OR (Liver, Nonalcoholic Fatty[Title/Abstract])) OR (Livers, Nonalcoholic Fatty[Title/Abstract])) OR (Nonalcoholic Fatty Liver[Title/Abstract])) OR (Nonalcoholic Fatty Livers[Title/Abstract])) OR (Nonalcoholic Steatohepatitis[Title/Abstract])) OR (Nonalcoholic Steatohepatitides[Title/Abstract])) OR (Steatohepatitides, Nonalcoholic[Title/Abstract])) OR (Steatohepatitis, Nonalcoholic[Title/Abstract]))) AND (("Empagliflozin"[Supplementary Concept]) OR ((((((Empagliflozin[Title/Abstract]) OR (1-chloro-4-(glucopyranos-1-yl)-2-(4-(tetrahydrofuran-3-yloxy)benzyl)benzene[Title/Abstract])) OR (BI 10773[Title/Abstract])) OR (BI10773[Title/Abstract])) OR (BI-10773[Title/Abstract])) OR (Jardiance[Title/Abstract])))

Web of science

TS= (Non-alcoholic Fatty Liver Disease OR Non alcoholic Fatty Liver Disease OR NAFLD OR Nonalcoholic Fatty Liver Disease OR Fatty Liver, Nonalcoholic OR Fatty Livers, Nonalcoholic OR Liver, Nonalcoholic Fatty OR Livers, Nonalcoholic Fatty OR Nonalcoholic Fatty Liver OR Nonalcoholic Fatty Livers OR Nonalcoholic Steatohepatitis OR Nonalcoholic Steatohepatitides OR Steatohepatitides, Nonalcoholic OR Steatohepatitis, Nonalcoholic) AND TS= （Empagliflozin OR 1-chloro-4-(glucopyranos-1-yl)-2-(4-(tetrahydrofuran-3-yloxy)benzyl)benzene OR BI 10773 OR BI10773 OR BI-10773 OR Jardiance）

Embase

#3 #1 AND #2
#2 Empagliflozin:ab,ti OR '1-chloro-4-(glucopyranos-1-yl)-2-(4-(tetrahydrofuran-3-yloxy)benzyl)benzene':ab,ti OR 'BI 10773':ab,ti OR BI10773:ab,ti OR 'BI-10773':ab,ti OR Jardiance:ab,ti
#1 'Non-alcoholic Fatty Liver Disease':ab,ti OR 'Non alcoholic Fatty Liver Disease':ab,ti OR NAFLD:ab,ti OR 'Nonalcoholic Fatty Liver Disease':ab,ti OR 'Fatty Liver, Nonalcoholic':ab,ti OR 'Fatty Livers, Nonalcoholic':ab,ti OR 'Liver, Nonalcoholic Fatty':ab,ti OR 'Livers, Nonalcoholic Fatty':ab,ti OR 'Nonalcoholic Fatty Liver':ab,ti OR 'Nonalcoholic Fatty Livers':ab,ti OR 'Nonalcoholic Steatohepatitis':ab,ti OR 'Nonalcoholic Steatohepatitides':ab,ti OR 'Steatohepatitides, Nonalcoholic':ab,ti OR 'Steatohepatitis, Nonalcoholic':ab,ti

Cochrane library

#3 #1 AND #2
#2 (Empagliflozin OR 1-chloro-4-(glucopyranos-1-yl)-2-(4-(tetrahydrofuran-3-yloxy)benzyl)benzene OR BI 10773 OR BI10773 OR BI-10773 OR Jardiance):ti,ab,kw

#1 (Non-alcoholic Fatty Liver Disease OR Non alcoholic Fatty Liver Disease OR NAFLD OR Nonalcoholic Fatty Liver Disease OR Fatty Liver, Nonalcoholic OR Fatty Livers, Nonalcoholic OR Liver, Nonalcoholic Fatty OR Livers, Nonalcoholic Fatty OR Nonalcoholic Fatty Liver OR Nonalcoholic Fatty Livers OR Nonalcoholic Steatohepatitis OR Nonalcoholic Steatohepatitides OR Steatohepatitides, Nonalcoholic OR Steatohepatitis, Nonalcoholic):ti,ab,kw

7. Exenatide

PubMed

(("Non-alcoholic Fatty Liver Disease"[Mesh]) OR (((((((((((((Non-alcoholic Fatty Liver Disease[Title/Abstract]) OR (Non alcoholic Fatty Liver Disease[Title/Abstract])) OR (NAFLD[Title/Abstract])) OR (Nonalcoholic Fatty Liver Disease[Title/Abstract])) OR (Fatty Liver, Nonalcoholic[Title/Abstract])) OR (Fatty Livers, Nonalcoholic[Title/Abstract])) OR (Liver, Nonalcoholic Fatty[Title/Abstract])) OR (Livers, Nonalcoholic Fatty[Title/Abstract])) OR (Nonalcoholic Fatty Liver[Title/Abstract])) OR (Nonalcoholic Fatty Livers[Title/Abstract])) OR (Nonalcoholic Steatohepatitis[Title/Abstract])) OR (Nonalcoholic Steatohepatitides[Title/Abstract])) OR (Steatohepatitides, Nonalcoholic[Title/Abstract])) OR (Steatohepatitis, Nonalcoholic[Title/Abstract]))) AND (("exenatide"[Mesh]) OR (((((((((exenatide[Title/Abstract]) OR (Bydureon[Title/Abstract])) OR (ITCA 650[Title/Abstract])) OR (AC 2993 LAR[Title/Abstract])) OR (Exendin-4[Title/Abstract])) OR (Ex4 Peptide[Title/Abstract])) OR (Peptide, Ex4[Title/Abstract])) OR (Exendin 4[Title/Abstract])) OR (Byetta[Title/Abstract])) OR (AC 2993[Title/Abstract])))

Web of science

TS= (Non-alcoholic Fatty Liver Disease OR Non alcoholic Fatty Liver Disease OR NAFLD OR Nonalcoholic Fatty Liver Disease OR Fatty Liver, Nonalcoholic OR Fatty Livers, Nonalcoholic OR Liver, Nonalcoholic Fatty OR Livers, Nonalcoholic Fatty OR Nonalcoholic Fatty Liver OR Nonalcoholic Fatty Livers OR Nonalcoholic Steatohepatitis OR Nonalcoholic Steatohepatitides OR Steatohepatitides, Nonalcoholic OR Steatohepatitis, Nonalcoholic) AND TS= （exenatide OR Bydureon OR ITCA 650 OR AC 2993 LAR OR Exendin-4 OR Ex4 Peptide OR Peptide, Ex4 OR Exendin 4 OR Byetta OR AC 2993）

Embase

#3 #1 AND #2

#2 exenatide:ab,ti OR Bydureon:ab,ti OR 'ITCA 650':ab,ti OR 'AC 2993 LAR':ab,ti OR 'Exendin-4':ab,ti OR 'Ex4 Peptide':ab,ti OR 'Peptide, Ex4':ab,ti OR 'Exendin 4':ab,ti OR Byetta:ab,ti OR 'AC 2993':ab,ti

#1 'Non-alcoholic Fatty Liver Disease':ab,ti OR 'Non alcoholic Fatty Liver Disease':ab,ti OR NAFLD:ab,ti OR 'Nonalcoholic Fatty Liver Disease':ab,ti OR 'Fatty Liver, Nonalcoholic':ab,ti OR 'Fatty Livers, Nonalcoholic':ab,ti OR 'Liver, Nonalcoholic Fatty':ab,ti OR 'Livers, Nonalcoholic Fatty':ab,ti OR 'Nonalcoholic Fatty Liver':ab,ti OR 'Nonalcoholic Fatty Livers':ab,ti OR 'Nonalcoholic Steatohepatitis':ab,ti OR 'Nonalcoholic Steatohepatitides':ab,ti OR 'Steatohepatitides, Nonalcoholic':ab,ti OR 'Steatohepatitis, Nonalcoholic':ab,ti

Cochrane library

#3 #1 AND #2

#2 (exenatide OR Bydureon OR ITCA 650 OR AC 2993 LAR OR Exendin-4 OR Ex4 Peptide OR Peptide, Ex4 OR Exendin 4 OR Byetta OR AC 2993):ti,ab,kw

#1 (Non-alcoholic Fatty Liver Disease OR Non alcoholic Fatty Liver Disease OR NAFLD OR Nonalcoholic Fatty Liver Disease OR Fatty Liver, Nonalcoholic OR Fatty Livers, Nonalcoholic OR Liver, Nonalcoholic Fatty OR Livers, Nonalcoholic Fatty OR Nonalcoholic Fatty Liver OR Nonalcoholic Fatty Livers OR Nonalcoholic Steatohepatitis OR Nonalcoholic Steatohepatitides OR Steatohepatitides, Nonalcoholic OR Steatohepatitis, Nonalcoholic):ti,ab,kw

8. Liraglutide

PubMed

(("Non-alcoholic Fatty Liver Disease"[Mesh]) OR (((((((((((((Non-alcoholic Fatty Liver Disease[Title/Abstract]) OR (Non alcoholic Fatty Liver Disease[Title/Abstract])) OR (NAFLD[Title/Abstract])) OR (Nonalcoholic Fatty Liver Disease[Title/Abstract])) OR (Fatty Liver, Nonalcoholic[Title/Abstract])) OR (Fatty Livers, Nonalcoholic[Title/Abstract])) OR (Liver, Nonalcoholic Fatty[Title/Abstract])) OR (Livers, Nonalcoholic Fatty[Title/Abstract])) OR (Nonalcoholic Fatty Liver[Title/Abstract])) OR (Nonalcoholic Fatty Livers[Title/Abstract])) OR (Nonalcoholic Steatohepatitis[Title/Abstract])) OR (Nonalcoholic Steatohepatitides[Title/Abstract])) OR (Steatohepatitides, Nonalcoholic[Title/Abstract])) OR (Steatohepatitis, Nonalcoholic[Title/Abstract]))) AND (("liraglutide"[Mesh]) OR (((((((liraglutide[Title/Abstract]) OR (Victoza[Title/Abstract])) OR (Saxenda[Title/Abstract])) OR (NN 2211[Title/Abstract])) OR (2211, NN[Title/Abstract])) OR (NN2211[Title/Abstract])) OR (NN-2211[Title/Abstract])))

Web of science

TS= (Non-alcoholic Fatty Liver Disease OR Non alcoholic Fatty Liver Disease OR NAFLD OR Nonalcoholic Fatty Liver Disease OR Fatty Liver, Nonalcoholic OR Fatty Livers, Nonalcoholic OR Liver, Nonalcoholic Fatty OR Livers, Nonalcoholic Fatty OR Nonalcoholic Fatty Liver OR Nonalcoholic Fatty Livers OR Nonalcoholic Steatohepatitis OR Nonalcoholic Steatohepatitides OR Steatohepatitides, Nonalcoholic OR Steatohepatitis, Nonalcoholic) AND TS= （liraglutide OR Victoza OR Saxenda OR NN 2211 OR 2211, NN OR NN2211 OR NN-2211）

Embase

#3 #1 AND #2

#2 liraglutide:ab,ti OR Victoza:ab,ti OR Saxenda:ab,ti OR 'NN 2211':ab,ti OR '2211, NN':ab,ti OR 'NN2211':ab,ti OR 'NN-2211':ab,ti

#1 'Non-alcoholic Fatty Liver Disease':ab,ti OR 'Non alcoholic Fatty Liver Disease':ab,ti OR NAFLD:ab,ti OR 'Nonalcoholic Fatty Liver Disease':ab,ti OR 'Fatty Liver, Nonalcoholic':ab,ti OR 'Fatty Livers, Nonalcoholic':ab,ti OR 'Liver, Nonalcoholic Fatty':ab,ti OR 'Livers, Nonalcoholic Fatty':ab,ti OR 'Nonalcoholic Fatty Liver':ab,ti OR 'Nonalcoholic Fatty Livers':ab,ti OR 'Nonalcoholic Steatohepatitis':ab,ti OR 'Nonalcoholic Steatohepatitides':ab,ti OR 'Steatohepatitides, Nonalcoholic':ab,ti OR 'Steatohepatitis, Nonalcoholic':ab,ti

Cochrane library

#3 #1 AND #2

#2 (liraglutide OR Victoza OR Saxenda OR NN 2211 OR 2211, NN OR NN2211 OR NN-2211):ti,ab,kw

#1 (Non-alcoholic Fatty Liver Disease OR Non alcoholic Fatty Liver Disease OR NAFLD OR Nonalcoholic Fatty Liver Disease OR Fatty Liver, Nonalcoholic OR Fatty Livers, Nonalcoholic OR Liver, Nonalcoholic Fatty OR Livers, Nonalcoholic Fatty OR Nonalcoholic Fatty Liver OR

Nonalcoholic Fatty Livers OR Nonalcoholic Steatohepatitis OR Nonalcoholic Steatohepatitides OR Steatohepatitides, Nonalcoholic OR Steatohepatitis, Nonalcoholic):ti,ab,kw

9. Dulaglutide

PubMed

(("Non-alcoholic Fatty Liver Disease"[Mesh]) OR (((((((((((((Non-alcoholic Fatty Liver Disease[Title/Abstract]) OR (Non alcoholic Fatty Liver Disease[Title/Abstract])) OR (NAFLD[Title/Abstract])) OR (Nonalcoholic Fatty Liver Disease[Title/Abstract])) OR (Fatty Liver, Nonalcoholic[Title/Abstract])) OR (Fatty Livers, Nonalcoholic[Title/Abstract])) OR (Liver, Nonalcoholic Fatty[Title/Abstract])) OR (Livers, Nonalcoholic Fatty[Title/Abstract])) OR (Nonalcoholic Fatty Liver[Title/Abstract])) OR (Nonalcoholic Fatty Livers[Title/Abstract])) OR (Nonalcoholic Steatohepatitis[Title/Abstract])) OR (Nonalcoholic Steatohepatitides[Title/Abstract])) OR (Steatohepatitides, Nonalcoholic[Title/Abstract])) OR (Steatohepatitis, Nonalcoholic[Title/Abstract]))) AND (("dulaglutide"[Supplementary Concept]) OR (((((dulaglutide[Title/Abstract]) OR (LY 2189265[Title/Abstract])) OR (LY-2189265[Title/Abstract])) OR (LY2189265[Title/Abstract])) OR (Trulicity[Title/Abstract])))

Web of science

TS= (Non-alcoholic Fatty Liver Disease OR Non alcoholic Fatty Liver Disease OR NAFLD OR Nonalcoholic Fatty Liver Disease OR Fatty Liver, Nonalcoholic OR Fatty Livers, Nonalcoholic OR Liver, Nonalcoholic Fatty OR Livers, Nonalcoholic Fatty OR Nonalcoholic Fatty Liver OR Nonalcoholic Fatty Livers OR Nonalcoholic Steatohepatitis OR Nonalcoholic Steatohepatitides OR Steatohepatitides, Nonalcoholic OR Steatohepatitis, Nonalcoholic) AND TS= （dulaglutide OR LY 2189265 OR LY-2189265 OR LY2189265 OR Trulicity）

Embase

#3 #1 AND #2

#2 dulaglutide:ab,ti OR 'LY 2189265':ab,ti OR 'LY-2189265':ab,ti OR 'LY2189265':ab,ti OR Trulicity:ab,ti

#1 'Non-alcoholic Fatty Liver Disease':ab,ti OR 'Non alcoholic Fatty Liver Disease':ab,ti OR NAFLD:ab,ti OR 'Nonalcoholic Fatty Liver Disease':ab,ti OR 'Fatty Liver, Nonalcoholic':ab,ti OR 'Fatty Livers, Nonalcoholic':ab,ti OR 'Liver, Nonalcoholic Fatty':ab,ti OR 'Livers, Nonalcoholic Fatty':ab,ti OR 'Nonalcoholic Fatty Liver':ab,ti OR 'Nonalcoholic Fatty Livers':ab,ti OR 'Nonalcoholic Steatohepatitis':ab,ti OR 'Nonalcoholic Steatohepatitides':ab,ti OR 'Steatohepatitides, Nonalcoholic':ab,ti OR 'Steatohepatitis, Nonalcoholic':ab,ti

Cochrane library

#3 #1 AND #2

#2 (dulaglutide OR LY 2189265 OR LY-2189265 OR LY2189265 OR Trulicity):ti,ab,kw

#1 (Non-alcoholic Fatty Liver Disease OR Non alcoholic Fatty Liver Disease OR NAFLD OR Nonalcoholic Fatty Liver Disease OR Fatty Liver, Nonalcoholic OR Fatty Livers, Nonalcoholic OR Liver, Nonalcoholic Fatty OR Livers, Nonalcoholic Fatty OR Nonalcoholic Fatty Liver OR Nonalcoholic Fatty Livers OR Nonalcoholic Steatohepatitis OR Nonalcoholic Steatohepatitides OR Steatohepatitides, Nonalcoholic OR Steatohepatitis, Nonalcoholic):ti,ab,kw

10. Semaglutide

PubMed

(("Non-alcoholic Fatty Liver Disease"[Mesh]) OR (((((((((((((Non-alcoholic Fatty Liver Disease[Title/Abstract]) OR (Non alcoholic Fatty Liver Disease[Title/Abstract])) OR (NAFLD[Title/Abstract])) OR (Nonalcoholic Fatty Liver Disease[Title/Abstract])) OR (Fatty Liver, Nonalcoholic[Title/Abstract])) OR (Fatty Livers, Nonalcoholic[Title/Abstract])) OR (Liver, Nonalcoholic Fatty[Title/Abstract])) OR (Livers, Nonalcoholic Fatty[Title/Abstract])) OR (Nonalcoholic Fatty Liver[Title/Abstract])) OR (Nonalcoholic Fatty Livers[Title/Abstract])) OR (Nonalcoholic Steatohepatitis[Title/Abstract])) OR (Nonalcoholic Steatohepatitides[Title/Abstract])) OR (Steatohepatitides, Nonalcoholic[Title/Abstract])) OR (Steatohepatitis, Nonalcoholic[Title/Abstract]))) AND (("semaglutide"[Supplementary Concept]) OR (((semaglutide[Title/Abstract]) OR (rybelsus[Title/Abstract])) OR (Ozempic[Title/Abstract])))

Web of science

TS= (Non-alcoholic Fatty Liver Disease OR Non alcoholic Fatty Liver Disease OR NAFLD OR Nonalcoholic Fatty Liver Disease OR Fatty Liver, Nonalcoholic OR Fatty Livers, Nonalcoholic OR Liver, Nonalcoholic Fatty OR Livers, Nonalcoholic Fatty OR Nonalcoholic Fatty Liver OR Nonalcoholic Fatty Livers OR Nonalcoholic Steatohepatitis OR Nonalcoholic Steatohepatitides OR Steatohepatitides, Nonalcoholic OR Steatohepatitis, Nonalcoholic) AND TS= （semaglutide OR rybelsus OR Ozempic）

Embase

#3 #1 AND #2

#2 semaglutide:ab,ti OR rybelsus:ab,ti OR Ozempic:ab,ti

#1 'Non-alcoholic Fatty Liver Disease':ab,ti OR 'Non alcoholic Fatty Liver Disease':ab,ti OR NAFLD:ab,ti OR 'Nonalcoholic Fatty Liver Disease':ab,ti OR 'Fatty Liver, Nonalcoholic':ab,ti OR 'Fatty Livers, Nonalcoholic':ab,ti OR 'Liver, Nonalcoholic Fatty':ab,ti OR 'Livers, Nonalcoholic Fatty':ab,ti OR 'Nonalcoholic Fatty Liver':ab,ti OR 'Nonalcoholic Fatty Livers':ab,ti OR 'Nonalcoholic Steatohepatitis':ab,ti OR 'Nonalcoholic Steatohepatitides':ab,ti OR 'Steatohepatitides, Nonalcoholic':ab,ti OR 'Steatohepatitis, Nonalcoholic':ab,ti

Cochrane library

#3 #1 AND #2

#2 (semaglutide OR rybelsus OR Ozempic):ti,ab,kw

#1 (Non-alcoholic Fatty Liver Disease OR Non alcoholic Fatty Liver Disease OR NAFLD OR Nonalcoholic Fatty Liver Disease OR Fatty Liver, Nonalcoholic OR Fatty Livers, Nonalcoholic OR Liver, Nonalcoholic Fatty OR Livers, Nonalcoholic Fatty OR Nonalcoholic Fatty Liver OR Nonalcoholic Fatty Livers OR Nonalcoholic Steatohepatitis OR Nonalcoholic Steatohepatitides OR Steatohepatitides, Nonalcoholic OR Steatohepatitis, Nonalcoholic):ti,ab,kw

Appendix 3 Included studies

Study	Year	Country	Age	%Male	Number of participants	Combined disease	Randomised treatments	Dose	Additional non-randomised intervention	NAFLD diagnostic method
Ohki et al	2012	China	54.2± 13.93	74.4	82	Type-2 diabetes	Liraglutide	0.3mg-0.9mg daily	Exercise and diet therapy	Ultrasonography
							Sitagliptin	50-100mg daily	Exercise and diet therapy	
							Pioglitazone	15mg daily	Exercise and diet therapy	
Fan et al	2013	China	52.35 ±11.83	56.4	117	Type-2 diabetes	Exenatide	5-10μg bid	Lifestyle interventions	Ultrasonography
							Metformin	0.5-2.0g bid	Lifestyle interventions	
Shao et al	2014	China	43± 4.1	48.3	60	Type-2 diabetes/Obesity	Intensive insulin	NR	Insulin glargine	Ultrasonography
			42± 3.2				Exenatide	5-10μg twice daily	Insulin glargine	
FENG et al	2017	China	47.15 ± 1.17	69	93	Type-2 diabetes	Liraglutide	0.6 mg/day during the first week, 1.2mg/day during the second week, and 1.8 mg/day from the third week to the conclusion of the study	Diet and exercise	Ultrasonography
							Gliclazide	30 mg before breakfast, which was gradually titrated to a maximum of 120 mg/day	Diet and exercise	

							Metformin	250 mg thrice a day during the first week, 500 mg thrice a day during the second week, and 1000 mg twice a day from the third week to the conclusion of the study	Diet and exercise	
Tian et al	2018	China	58.5 ± 7.6	58.27%	127	Type-2 diabetes	liraglutide	0.6-1.2 mg/day		B-mode ultrasonic
			56.4 ± 8.4				Metformin	1000-1500 mg/day		
Yan et al	2019	China	43.1 ± 9.7	70.8	24	Type-2 diabetes	Liraglutide	1.8mg daily	Metformin	MRI-PDFF
			45.7 ± 9.2	77.8	27		Sitagliptin	100mg daily	Metformin	
			45.6 ± 7.6	58.3	24		Insulin glargine	0.2IU/Kg/day	Metformin	
Zhang et al	2020	China	50.2 ± 11.5	43.3	30	Type-2 diabetes	Liraglutide	0.6-1.2mg daily	Metformin	MRI
			51.5 ± 12.1	50.0	30		Pioglitazone	15-30mg daily	Metformin	
Liu et al	2020	China	47.63 ± 10.14	54.3	35	Ttype 2 diabetes	Exenatide	5μg/10μg bid	Diet-exercise	MRI
			50.56 ± 11.78	52.8	36		Insulin glargine	0.1-0.3 IU/Kg daily	Diet-exercise	
Guo et al	2020	China	52.0 ± 8.7	60.0	91	Type-2 diabetes	Insulin glargine	10IU		Liver biopsy or ultrasongraphy

			53.1 ± 6.3	52.0		Type-2 diabetes	Liraglutide	0.6mg1/day-1.8mg		
			52.6 ± 3.9	67.0		Type-2 diabetes	Placebo			
Jiang et al	2020	China	44.59 ± 5. 17	56.9	116	Type-2 diabetes	Liraglutide	0. 6 mg-1. 2 mg/day	Melbine， insulin	Guideline diagnostic Criteria
			43.14 ± 6. 25	55.2		Type-2 diabetes	Metformin	0.75g/day	Insulin	
Pang et al	2020	China	45.0± 3.8	47.1	204	Type-2 diabetes	Pioglitazone	30 mg/day	Metformin	CT
			45.9± 3.5	41.2		Type-2 diabetes	Daglitazine	10 mg/day	Metformin	
Armstrong et al	2016	UK	NR	64.3	14	NR	Placebo	1.8mg daily	NR	Liver biopsy
							Liraglutide	0.6mg-1.8mg daily	NR	
Armstrong et al	2016	UK	51±11 .44	59.6	52	NR	Liraglutide	0.6mg-1.8mg daily	NR	Liver biopsy
							Placebo		NR	
Newsome et al	2021	UK	55	39.0	320	With and without Type-2 diabetes	Semaglutide	0.1 mg once daily		Liver biopsy
							Placebo	0.1-0.4 mg once daily		
Newsome et al	2021	UK	55	39.0	320	With and without Type-2 diabetes	Semaglutide	0.4 mg once daily		
							Placebo	0.1-0.4 mg once daily		

Newsome et al	2021	UK	55	39.0	320	With and without Type-2 diabetes	Semaglutide	0.2 mg once daily		
							Placebo	0.1-0.4 mg once daily		
Ito et al	2017	Japan	59.1 ± 9.8	48.48%	66	Type-2 diabetes	Pioglitazone	15-30mg daily	Diet 、exercise and diabetes treatment	CT or ultrasound
			57.3 ± 12.1				Ipragliflozin	50mg daily	Diet 、exercise and diabetes treatment	
Shibuya et al	2018	Japan	51 ± 11.11	56.25%	32	Type-2 diabetes	Luseogliflozin	2.5 mg daily		CT or ultrasound
			60 ± 9.63				Metformin	1500 mg daily		
Aso et al	2019	Japan	NR	NR	57	Type-2 diabetes	Dapagliflozin	5mg/day		Ultrasonography
							Control			
Shimizu et al	2019	Japan	56.2 ± 11.5	57.6	33	Type-2 diabetes	Dapagliflozin	5 mg daily		Ultrasonography
			57.1 ± 13.8	62.5	24		Non-SGLT-2		Intensifying diet and exercise	
Kinoshita T et al	2020	Japan	58.7 ± 1.6	46.9	32	Type 2 diabetes	Dapagliflozin	5mg daily	Baseline antidiabetic drugs	Computed tomography
			59.0 ± 1.9	45.5	33		Pioglitazone	7.5-15mg daily	Baseline antidiabetic drugs	
			58.0 ± 2.3	45.5	33		Glimepiride	0.5-1mg daily	Baseline antidiabetic drugs	
Aso et al	2021	Japan			57	Type-2 diabetes	Dapagliflozin	5 mg/d		Ultrasonography
						Type-2	Standard treatment for			

						diabetes	type 2 diabetes			
Tobita et al	2021	Japan	47.15 ±14.99	68.2	22	Metabolic syndrome/Hypertension/Dyslipidemia	Dapagliflozin	5mg once daily		Ultrasonography
							Teneligliptin	20mg once daily		
Yoneda et al	2021	Japan	58.59 ±10.33	52.5	40	Type-2 diabetes	Pioglitazone	15-30mg once a day		MRI
							Tofogliflozin	20mg once a day		
Cho et al	2021	Japan	63.45 ±8.67	52.8	53	Type-2 diabetes	Dapagliflozin			Fatty liver index (FLI)
							Pioglitazone			
Takahashi et al	2021	Japan	55.0 (47.0-65.0)	14 (66.7)	46	Type-2 diabetes	Ipragliflozin	50 mg/day		Liver biopsy
			50.0 (48.0-69.0)	13 (52.0)			Enhanced lifestyle modification including antidiabetic agents except SGLT2i，pioglitazone and GLP-1 analog			
Kuchay et al	2018	India	NR	60%	42	Type-2 diabetes	Control		Diabetes treatment	MRI
							Empagliflozin	10 mg daily	Diabetes treatment	
Hussain et al	2021	India	29±16	50.4	138	Type-2 diabetes	Dapaglifozin	5–10 mg	Glimepiride	Ultrasonography
			31±14	50.7		Type-2 diabetes	Placebo		Glimepiride	
Khoo et al	2017	Singapore	41.35 ±9.43	91.7	24	Obese	Diet and exercise	dieting (restriction by 400 kilocalories/day and	NR	Magnetic resonance

								exercise (200 minutes/week)		imaging (MRI)
							Liraglutide	3 mg daily	NR	
Khoo et al	2019	Singapore	38.6 ± 8.2	100.0	15	Obesity	Liraglutide	0.6-3.0mg daily		MRI
			43.6 ± 9.9	86.7	15		Diet-exercise			
Taheri et al	2020	Iran	43.8 ± 9.7	65.1	43		Empagliflozin	10mg daily	Diet-exercise	Ultrasound
			44.1 ± 9.3	46.8	47		Placebo		Diet-exercise	
Chehrehgosha et al	2021	Iran	50.5 ± 8.4	42.9	106	Type-2 diabetes	Empaglifflozin	10 mg		Not clear
			52.5 ± 7.9	50.0		Type-2 diabetes	Pioglitazone	30 mg		
			51.8 ± 7.8	37.8		Type-2 diabetes	Placebo			
Flint et al	2021	Germany	60.0± 9.3	70.1	67	With Type-2 diabetes	Semaglutide	0.4 mg once daily		MRI
							Placebo	0.4 mg once daily		
Flint et al	2021	Germany	60.0± 9.3	70.1	67	Without Type-2 diabetes	Semaglutide	0.4 mg once daily		MRI
							Placebo	0.4 mg once daily		
Savvidou et al	2016	Greece	62.9± 7.1	39.1	103	Type-2 diabetes	Exenatide	5-10μg twice daily	NR	Liver biopsy
							Insulin	NR	NR	
Eriksson et al	2018	Sweden	65.6 ±6.1	70.24%	84	Type-2 diabetes	Placebo			MRI
			66.2± 5.9				OM-3CA	4 g daily		

			65.0 ±6.5				Dapagliflozin	10 mg daily		
			65.0± 5.4				OM-3CA + dapagliflozin	10mg+4 g daily		
Kuchay et al	2020	Germany	46.6 ± 9.1	72.0	64	Type-2 diabetes	Dulaglutide	0.75mg 4week then 1.5mg 20week	Standard treatment for type 2 diabetes	MRI
			48.1 ± 8.9	69.0			Metformin,DPP-4 inhibitors in the control group,sulfonylureas and/or insulin	standard treatment for type 2 diabetes		
Vedtofte et al	2020	Denmark	38.8± 4.7	0.0	18	Gestational Diabetes Mellitus	Liraglutide	1.8 mg daily		Ultrasonography
			38.3± 4.2	0.0		Gestational Diabetes Mellitus	Placebo			
Han et al	2020	Korea	52.5 ± 10.3	63.3	44	Type-2 diabetes	Ipragliflflozin，metformin and pioglitazone	50 mg/day	Metformin and pioglitazone	Ultrasonography
			56.7 ± 11.8	60.0		Type-2 diabetes	Metformin and pioglitazone			
Phrueksotsai et al	2021	Thailand	59.2 ± 7.3	31.6	38	Type-2 diabetes	Dapagliflozin	10 mg/day		Ultrasonography or CT
							Placebo	10 mg/day		

Appendix 4 Reference list for included studies

Ohki 2012

Ohki T, Isogawa A, Iwamoto M, et al. The effectiveness of liraglutide in nonalcoholic fatty liver disease patients with type 2 diabetes mellitus compared to sitagliptin and pioglitazone. *The Scientific World Journal* 2012;2012.

Fan 2013

Fan H, Pan Q, Xu Y, et al. Exenatide improves type 2 diabetes concomitant with non-alcoholic fatty liver disease. *Arquivos brasileiros de endocrinologia e metabologia* 2013;57(9):702-08.

Shao 2014

Shao N, Kuang HY, Hao M, et al. Benefits of exenatide on obesity and non-alcoholic fatty liver disease with elevated liver enzymes in patients with type 2 diabetes. *Diabetes/metabolism research and reviews* 2014;30(6):521-29.

Savvidou 2016

Savvidou S, Karatzidou K, Tsakiri K, et al. Circulating adiponectin levels in type 2 diabetes mellitus patients with or without non-alcoholic fatty liver disease: results of a small, open-label, randomized controlled intervention trial in a subgroup receiving short-term exenatide. *Diabetes research and clinical practice* 2016;113:125-34.

Armstrong 2016

Armstrong MJ, Hull D, Guo K, et al. Glucagon-like peptide 1 decreases lipotoxicity in non-alcoholic steatohepatitis. *J Hepatol* 2016;64(2):399-408.

Armstrong 2016

Armstrong MJ, Gaunt P, Aithal GP, et al. Liraglutide safety and efficacy in patients with non-alcoholic steatohepatitis (LEAN): a multicentre, double-blind, randomised, placebo-controlled phase 2 study. *Lancet (london, england)* 2016;387(10019):679-90.

Feng 2017

Feng W, Gao C, Bi Y, et al. Randomized trial comparing the effects of gliclazide, liraglutide, and metformin on diabetes with non-alcoholic fatty liver disease. *Journal of diabetes* 2017;9(8):800-09.

Khoo 2017

Khoo J, Hsiang J, Taneja R, et al. Comparative effects of liraglutide 3 mg vs structured lifestyle modification on body weight, liver fat and liver function in obese patients with non-alcoholic fatty liver disease: A pilot randomized trial. *Diabetes Obesity & Metabolism* 2017;19(12):1814-17.

Ito 2017

Ito D, Shimizu S, Inoue K, et al. Comparison of Ipragliflozin and Pioglitazone Effects on Nonalcoholic Fatty Liver Disease in Patients With Type 2 Diabetes: a Randomized, 24-Week, Open-Label, Active-Controlled Trial. *Diabetes care* 2017;40(10):1364-72.

Tian 2018

Tian F, Zheng Z, Zhang D, et al. Efficacy of liraglutide in treating type 2 diabetes mellitus complicated with non-alcoholic fatty liver disease. *Bioscience reports* 2018;38(6).

Shibuya 2018

Shibuya T, Fushimi N, Kawai M, et al. Luseogliflozin improves liver fat deposition compared to metformin in type 2 diabetes patients with non-alcoholic fatty liver disease: a prospective randomized controlled pilot study. *Diabetes, obesity & metabolism* 2018;20(2):438-42.

Eriksson 2018

Eriksson JW, Lundkvist P, Jansson PA, et al. Effects of dapagliflozin and n-3 carboxylic acids on non-alcoholic fatty liver disease in people with type 2 diabetes: a double-blind randomised placebo-controlled study. *Diabetologia* 2018:1-12.

Kuchay 2018

Kuchay MS, Krishan S, Mishra SK, et al. Effect of empagliflozin on liver fat in patients with type 2 diabetes and nonalcoholic fatty liver disease: A randomized controlled trial (E-LIFT Trial). *Diabetes Care* 2018;41(8):1801-08.

Aso 2019

Aso Y, Kato K, Sakurai S, et al. Impact of dapagliflozin, an SGLT2 inhibitor, on serum levels of soluble dipeptidyl peptidase-4 in patients with type 2 diabetes and non-alcoholic fatty liver disease. *International journal of clinical practice* 2019;73(5):e13335.

Khoo 2019

Khoo J, Hsiang JC, Taneja R, et al. Randomized trial comparing effects of weight loss by liraglutide with lifestyle modification in non-alcoholic fatty liver disease. *Liver international* 2019;39(5):941-49.

Shimizu 2019

Shimizu M, Suzuki K, Kato K, et al. Evaluation of the effects of dapagliflozin, a sodium-glucose co-transporter-2 inhibitor, on hepatic steatosis and fibrosis using transient elastography in patients with type 2 diabetes and non-alcoholic fatty liver disease. *Diabetes, Obesity and Metabolism* 2019;21(2):285-92.

Yan 2019

Yan J, Yao B, Kuang H, et al. Liraglutide, Sitagliptin, and Insulin Glargine Added to Metformin: the Effect on Body Weight and Intrahepatic Lipid in Patients With Type 2 Diabetes Mellitus and Nonalcoholic Fatty Liver Disease. *Hepatology (Baltimore, Md)* 2019;69(6):2414-26.

Zhang 2020

Zhang LY, Qu XN, Sun ZY, et al. Effect of liraglutide therapy on serum fetuin A in patients with type 2 diabetes and non-alcoholic fatty liver disease. *Clinics and research in hepatology and gastroenterology* 2020;44(5):674-80.

Taheri 2020

Taheri H, Malek M, Ismail-Beigi F, et al. Effect of Empagliflozin on Liver Steatosis and Fibrosis in Patients With Non-Alcoholic Fatty Liver Disease Without Diabetes: a Randomized,

Double-Blind, Placebo-Controlled Trial. *Advances in therapy* 2020;37(11):4697-708.

Kinoshita 2020

Kinoshita T, Shimoda M, Nakashima K, et al. Comparison of the effects of three kinds of glucose-lowering drugs on non-alcoholic fatty liver disease in patients with type 2 diabetes: a randomized, open-label, three-arm, active control study. *Journal of diabetes investigation* 2020;11(6):1612-22.

Liu 2020

Liu L, Yan H, Xia M, et al. Efficacy of exenatide and insulin glargine on nonalcoholic fatty liver disease in patients with type 2 diabetes. *Diabetes/Metabolism Research and Reviews* 2020;36(5).

Kuchay 2020

Kuchay MS, Krishan S, Mishra SK, et al. Effect of dulaglutide on liver fat in patients with type 2 diabetes and NAFLD: randomised controlled trial (D-LIFT trial). *Diabetologia* 2020;63(11):2434-45.

Vedtofte 2020

Vedtofte L, Bahne E, Foghsgaard S, et al. One year's treatment with the glucagon-like peptide 1 receptor agonist liraglutide decreases hepatic fat content in women with nonalcoholic fatty liver disease and prior gestational diabetes mellitus in a randomized, placebo-controlled trial. *Journal of Clinical Medicine* 2020;9(10):1-14.

Han 2020

Han E, Lee YH, Lee BW, et al. Ipragliflozin additively ameliorates non-alcoholic fatty liver disease in patients with type 2 diabetes controlled with metformin and pioglitazone: a 24-week randomized controlled trial. *Journal of clinical medicine* 2020;9(1).

Guo 2020

Guo W, Tian W, Lin L, et al. Liraglutide or insulin glargine treatments improves hepatic fat in obese patients with type 2 diabetes and nonalcoholic fatty liver disease in twenty-six weeks: a randomized placebo-controlled trial. *Diabetes research and clinical practice* 2020;170:108487.

Jiang 2020

Jiang Z, Chen J. Clinical trial of liraglutide in the treatment of patients with type 2 diabetes mellitus and non-alcoholic fatty liver. *The Chinese Journal of Clinical Pharmacology* 2020;36(4):400-03.

Pang 2020

Pang Q, Wang M, Gao Q, et al. Comparative efficacy of daglitazine or pioglitazone combined with metform in patients with type 2 diabetes mellitus and non-alcoholic fatty liver. *Chinese Journal of New Drugs and Clinical Remedies* 2020;39(11):675-79.

Hussain 2021

Hussain M, Babar MZM, Tariq S, et al. Therapeutic outcome of dapagliflozin on various parameters

in non-alcoholic fatty liver disease (NAFLD) patients. *International journal of diabetes in developing countries* 2021.

Aso 2021

Aso Y, Sagara M, Niitani T, et al. Serum high-molecular-weight adiponectin and response to dapagliflozin in patients with type 2 diabetes and non-alcoholic fatty liver disease. *Journal of investigative medicine* 2021.

Chehrehgosha 2021

Chehrehgosha H, Sohrabi MR, Ismail-Beigi F, et al. Empagliflozin Improves Liver Steatosis and Fibrosis in Patients with Non-Alcoholic Fatty Liver Disease and Type 2 Diabetes: a Randomized, Double-Blind, Placebo-Controlled Clinical Trial. *Diabetes therapy* 2021.

Tobita 2021

Tobita H, Yazaki T, Kataoka M, et al. Comparison of dapagliflozin and teneligliptin in nonalcoholic fatty liver disease patients without type 2 diabetes mellitus: A prospective randomized study. *Journal of Clinical Biochemistry and Nutrition* 2021;68(2):173-80.

Yoneda 2021

Yoneda M, Honda Y, Ogawa Y, et al. Comparing the effects of tofogliflozin and pioglitazone in non-Alcoholic fatty liver disease patients with type 2 diabetes mellitus (ToPiND study): A randomized prospective open-label controlled trial. *BMJ Open Diabetes Research and Care* 2021;9(1).

Phrueksotsai 2021

Phrueksotsai S, Pinyopornpanish K, Euathrongchit J, et al. The effects of dapagliflozin on hepatic and visceral fat in type 2 diabetes patients with non-alcoholic fatty liver disease. *Journal of gastroenterology and hepatology (australia)* 2021.

Cho 2021

Cho KY, Nakamura A, Omori K, et al. Favorable effect of sodium-glucose cotransporter 2 inhibitor, dapagliflozin, on non-alcoholic fatty liver disease compared with pioglitazone. *Journal of diabetes investigation* 2021;12(7):1272-77.

Takahashi 2021

Takahashi H, Kessoku T, Kawanaka M, et al. Ipragliflozin Improves the Hepatic Outcomes of Patients With Diabetes with NAFLD. *Hepatology communications* 2021.

Flint 2021

Flint A, Andersen G, Hockings P, et al. Randomised clinical trial: semaglutide versus placebo reduced liver steatosis but not liver stiffness in subjects with non-alcoholic fatty liver disease assessed by magnetic resonance imaging. *Alimentary pharmacology & therapeutics* 2021;54(9):1150-61.

Newsome 2021

Newsome PN, Buchholtz K, Cusi K, et al. A Placebo-Controlled Trial of Subcutaneous Semaglutide in Nonalcoholic Steatohepatitis. *New England journal of medicine* 2021;384(12):1113-24.

Appendix 5 Risk of bias in included studies

Study	Trial registration	Number of participants	Randomization process	Deviations from the intended interventions	Measurement of the outcome	Missing outcome data	Selection of the reported result
Ohki et al（2012）	No provided	82	Unclear	Unclear	Low	Low	Low
Fan et al（2013）	No provided	117	Unclear	Unclear	Low	Low	Low
Shao et al（2014）	No provided	60	Low	High	Low	Low	Low
Savviou et al（2016）	No provided	103	Low	Low	Low	Low	Low
Armstrong et al（2016）	NCT01237119	14	Low	Low	Low	Low	Low
Armstrong et al（2016）	NCT01237119	52	Low	Low	Low	Low	Unclear
FENG et al（2017）	NCT03068065,	93	Low	High	Low	Low	Low
Khoo et al（2017）	No provided	24	Low	High	Unclear	Low	Low
Ito et al（2017）	UMIN 000022651	66	Low	High	Low	Unclear	Unclear
Tian et al（2018）	No provided	127	Low	Low	Low	Unclear	Low
Shibuya et al（2018）	UMIN000016090	32	Low	High	Low	Low	Unclear
W. Eriksson et al（2018）	NCT02279407	84	Low	Low	Low	Unclear	Low
Kuchay et al（2018）	NCT02686476	42	Low	Unclear	Low	Unclear	Low
Aso et al（2019）	UMIN000022155	57	Low	High	Low	Low	Low
Khoo et al（2019）	NCT02654665	15	Low	Low	Low	Low	Unclear
Shimizu et al（2019）	UMIN000022155	33	Low	Low	Unclear	Low	Unclear
Yan et al（2019）	NCT02147925	24	Low	Unclear	Low	Low	Unclear
Zhang et al（2020）	No applicable	30	Low	Low	Unclear	Low	Low
Taheri et al（2020）	IRCT20190122042450 N1	43	Low	Low	Low	Unclear	Low
Kinoshita et al(2020)	UMIN 000021291	98	Low	Low	Low	Low	Low
Liu et al(2020)	ESR-14-10096	76	Low	High	Low	Low	Low
Kuchay et al(2020)	NCT03590626	64	Low	Low	Unclear	Low	Low
Vedtofte et al(2020)	EudraCT number: 2012-001371-27	82	Low	Low	Low	Low	High
Han et al(2020)	NCT02875821	44	Low	High	Unclear	Low	Low
Guo et al(2020)	ChiCTR2000035091	91	Low	High	High	Low	Low
Jiang et al(2020)	Not provided	204	Low	Low	Low	Low	High
Pang et al(2020)	Not provided	116	Low	High	Unclear	Low	Low
Hussain et al(2021)	Not provided	450	Low	Low	Low	Low	High
Aso et al(2021)	UMIN000022155	57	Low	High	Low	Low	Low
Chehrehgosha et al(2021)	IRCT20190122042450 N3	186	Unclear	Low	Low	Unclear	Low
Tobita(2021)	UMIN000027304	22	Low	Low	Low	Low	Low
Yoneda(2021)	jRCTs031180159	40	Low	Unclear	Unclear	Low	Low
Phrueksotsai(2021)	TCTR20170511001	38	Low	Low	Low	Low	High
Cho(2021)	UMIN000022804	53	Low	High	Low	Low	Low
Takahashi(2021)	UMIN000015727 and jRCTs071180069	50	Unclear	Low	Low	Low	Low
Flint(2021)	NCT03357380	67	Low	Low	Unclear	Low	Low
Newsome(2021)	NCT02970942	320	Low	Low	Low	Low	Low

Appendix 6 Network plots for each outcome

The size of the circle in each network is proportional to the number of participants randomly assigned to the treatment comparison. The width of each line is proportional to the number of trials comparing the two connected treatments. When a line is absent, this indicates that there were no head-to-head trials of the corresponding treatments reporting the outcome of interest. The number provided for each treatment class (in parentheses) indicates the number of patients assigned to the treatment in the network.

Figure 1 Network plot for alanine aminotransferase (ALT)

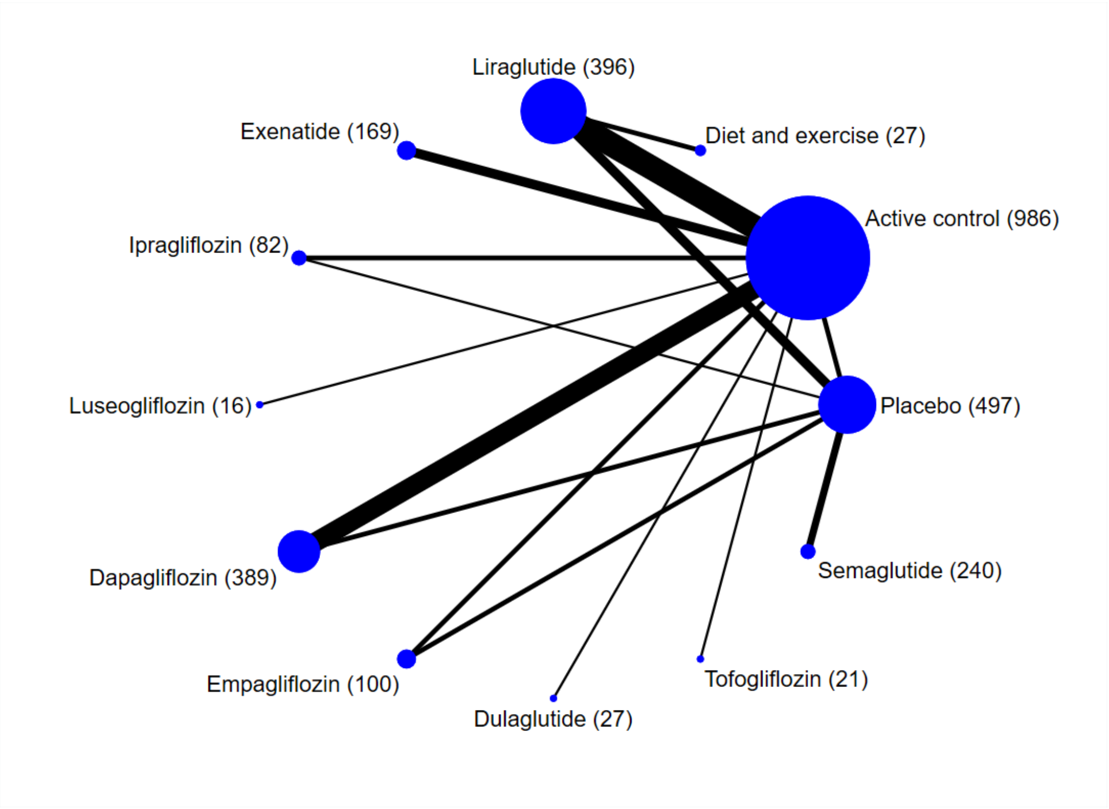

Figure 2 Network plot for aspartate aminotransferase (AST)

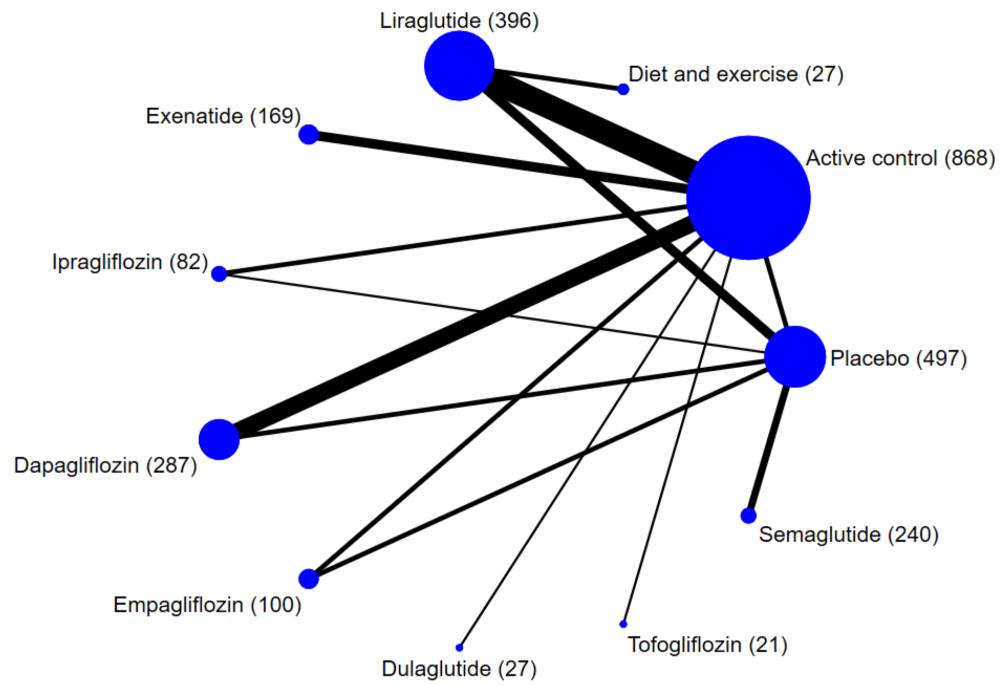

Figure 3 Network plot for γ-glutamyl transferase (GGT)

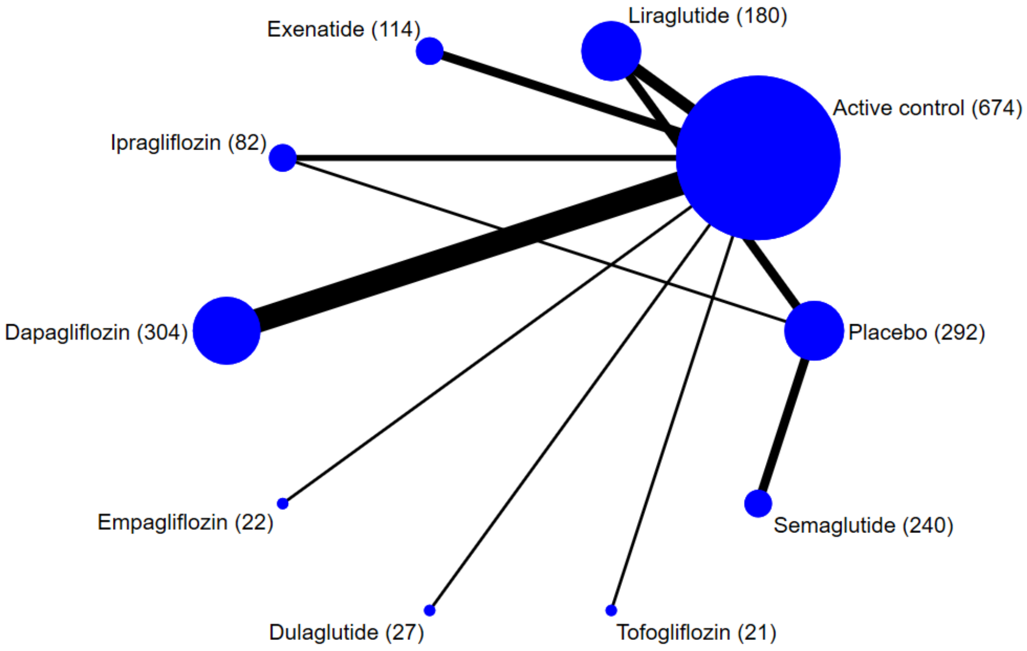

Figure 4 Network plot for subcutaneous adipose tissue (SAT)

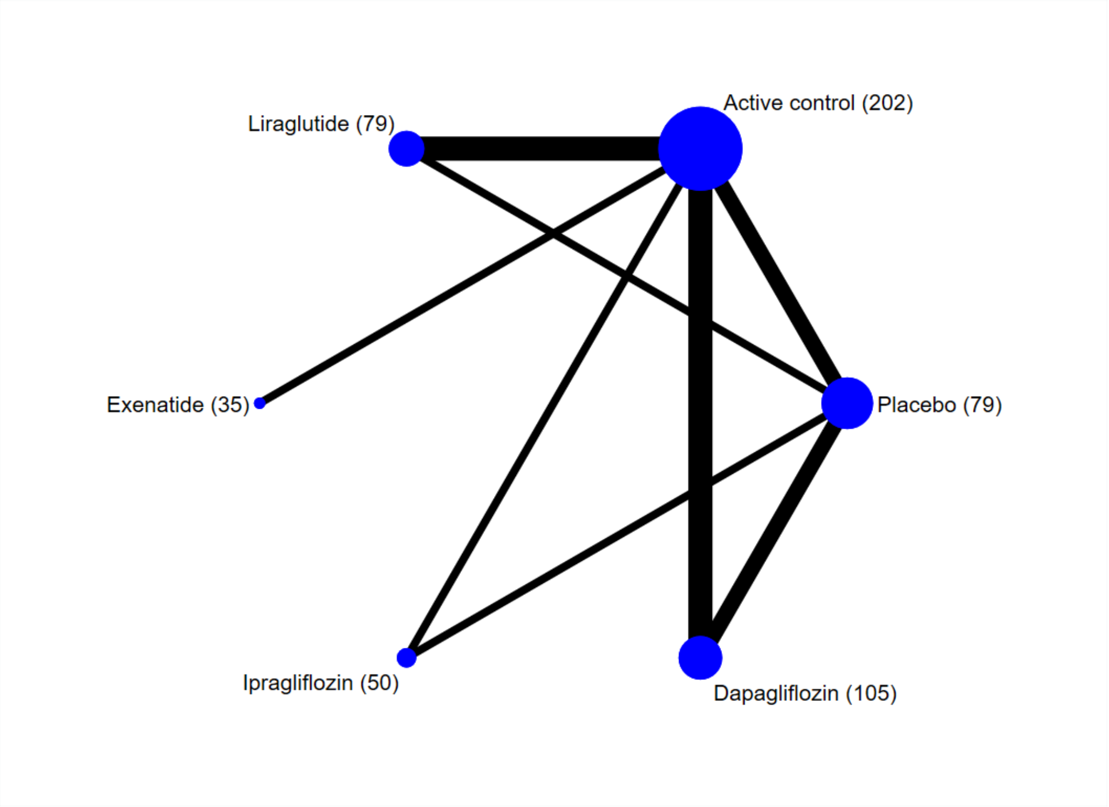

Figure 5 Network plot for visceral adipose tissue (VAT)

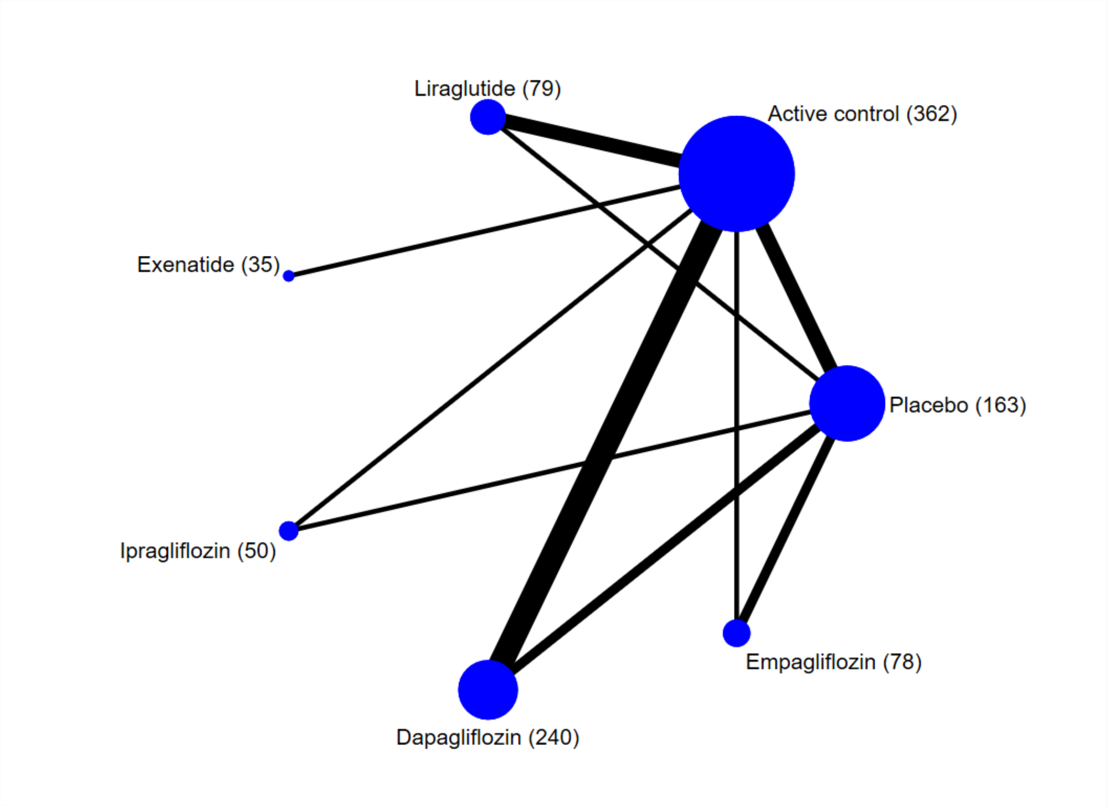

Figure 6 Network plot for liver fat fraction (LFF)

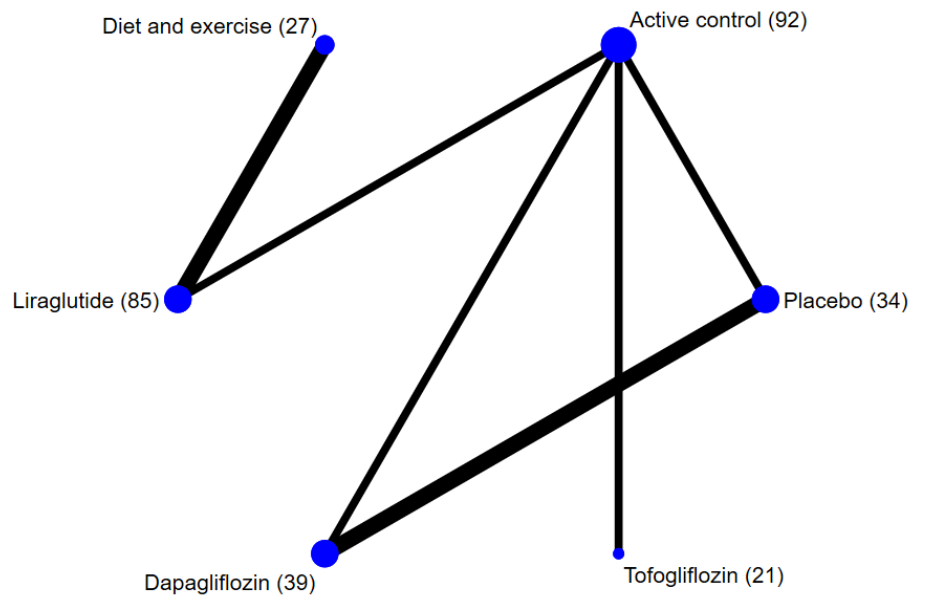

Figure 7 Network plot for controlled attenuation parameter (CAP)

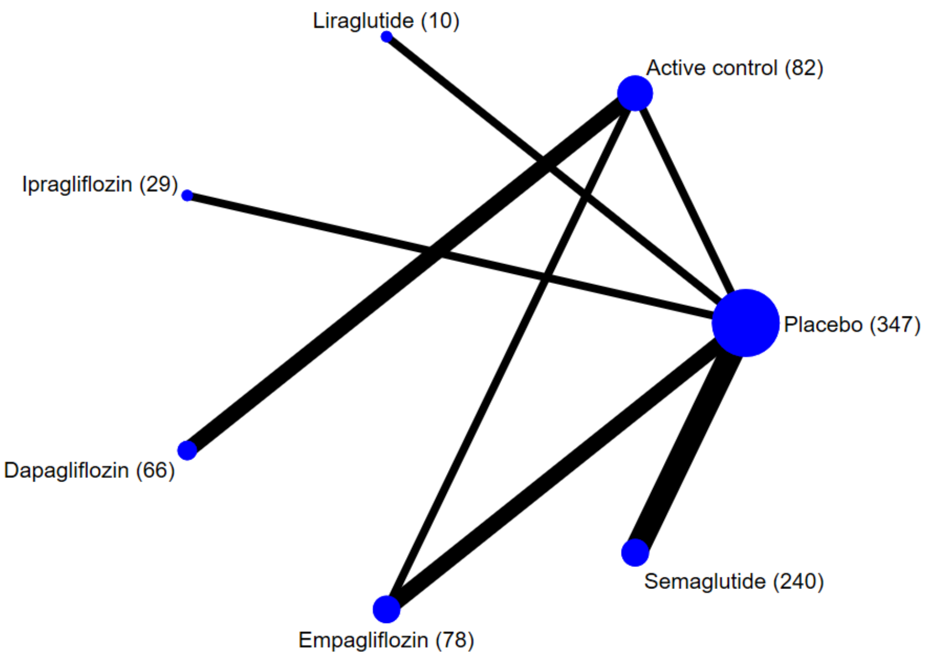

Figure 8 Network plot for liver stiffness measurement (LSM)

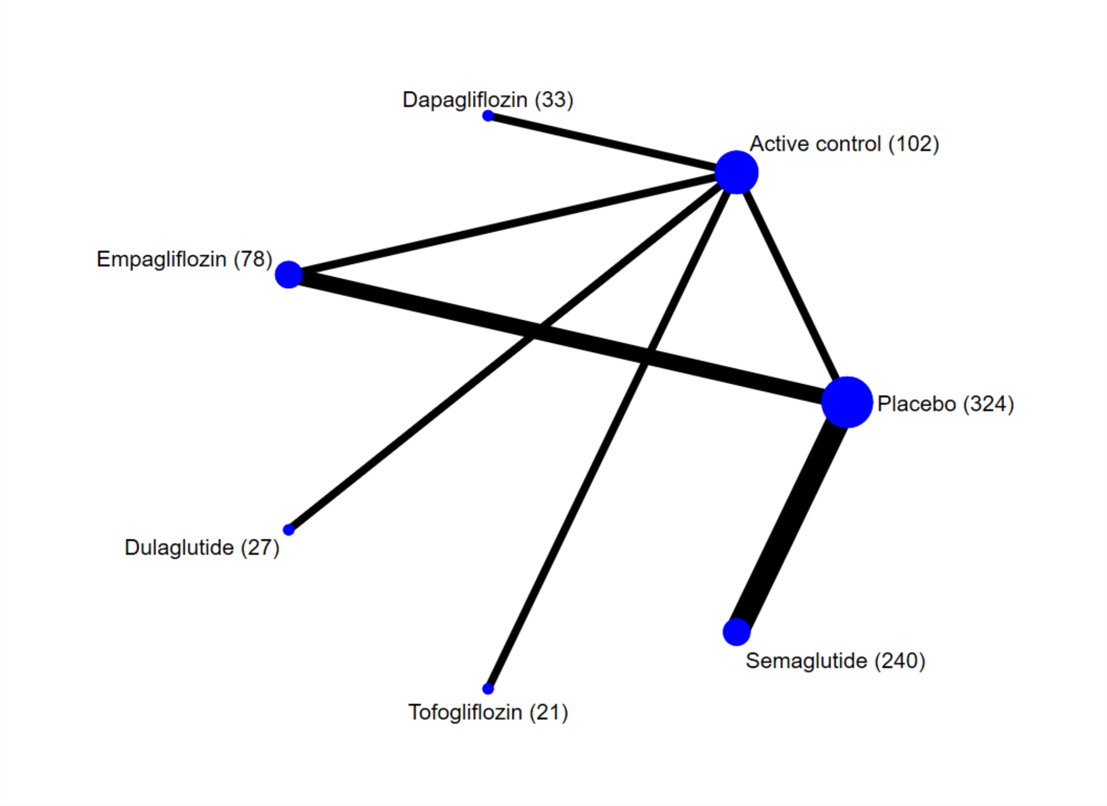

Figure 9 Network plot for body weight (BW)

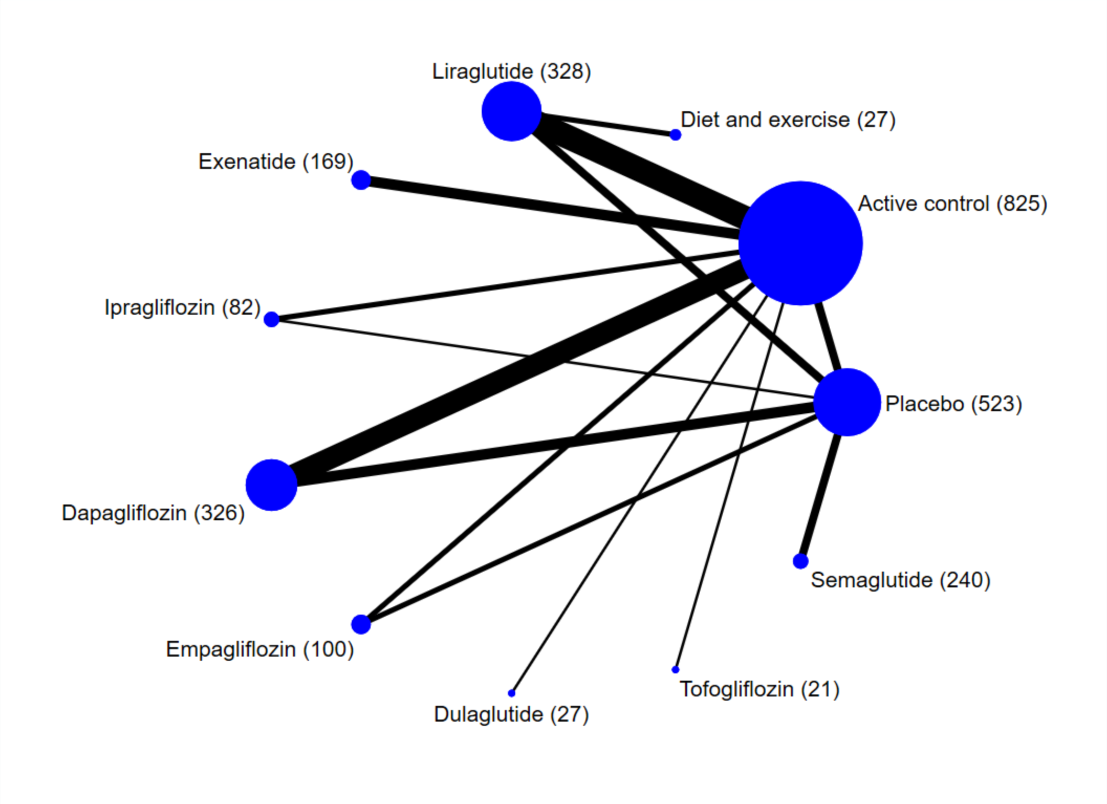

Figure 10 Network plot for body mass index (BMI)

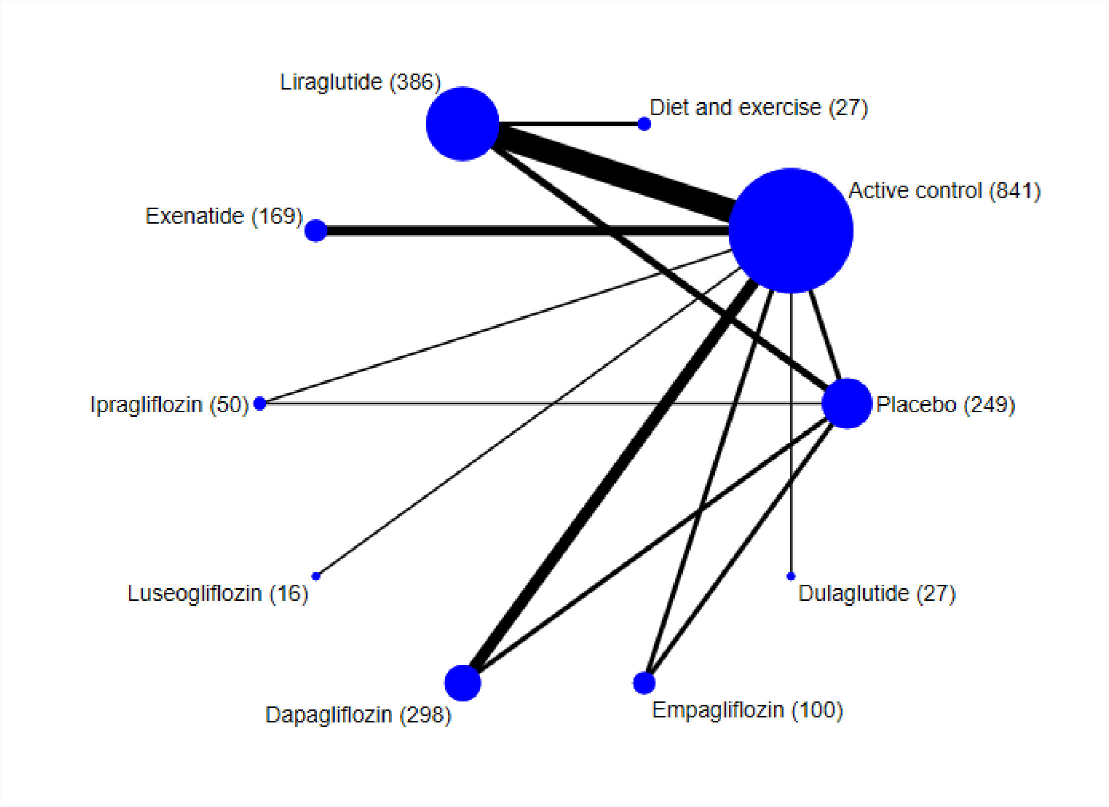

Figure 11 Network plot for waist circumference (WC)

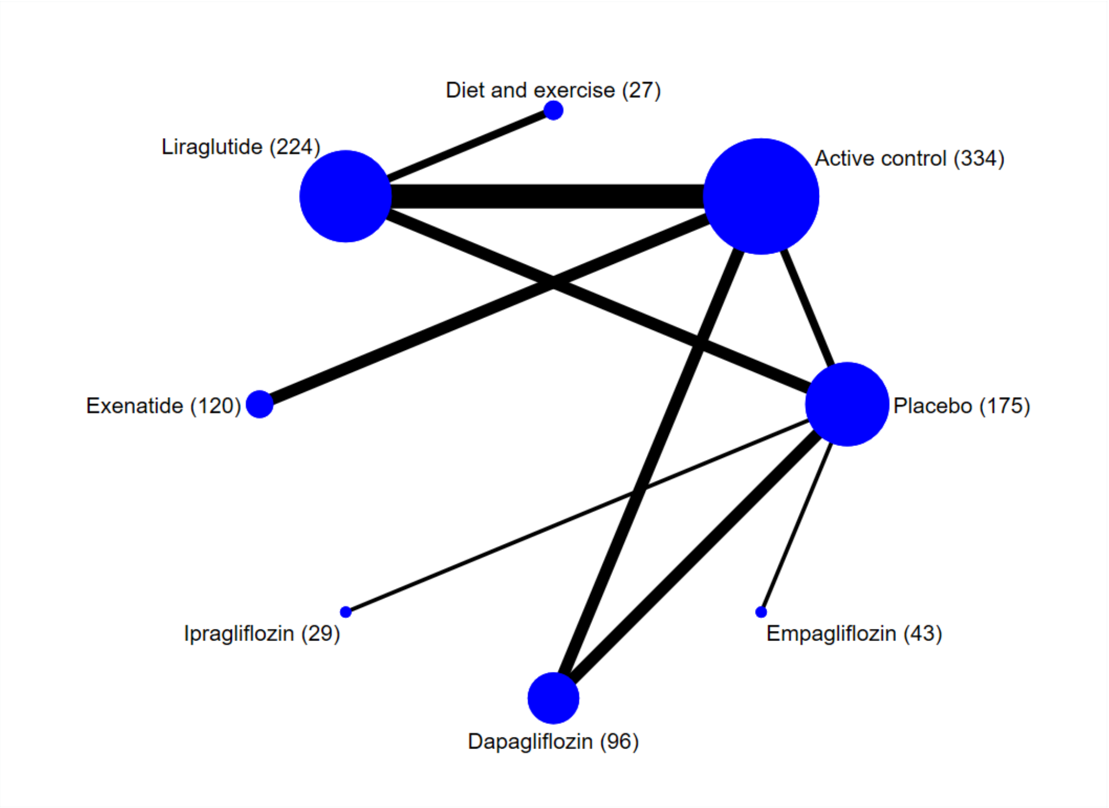

Figure 12 Network plot for systobic blood pressure (SBP)

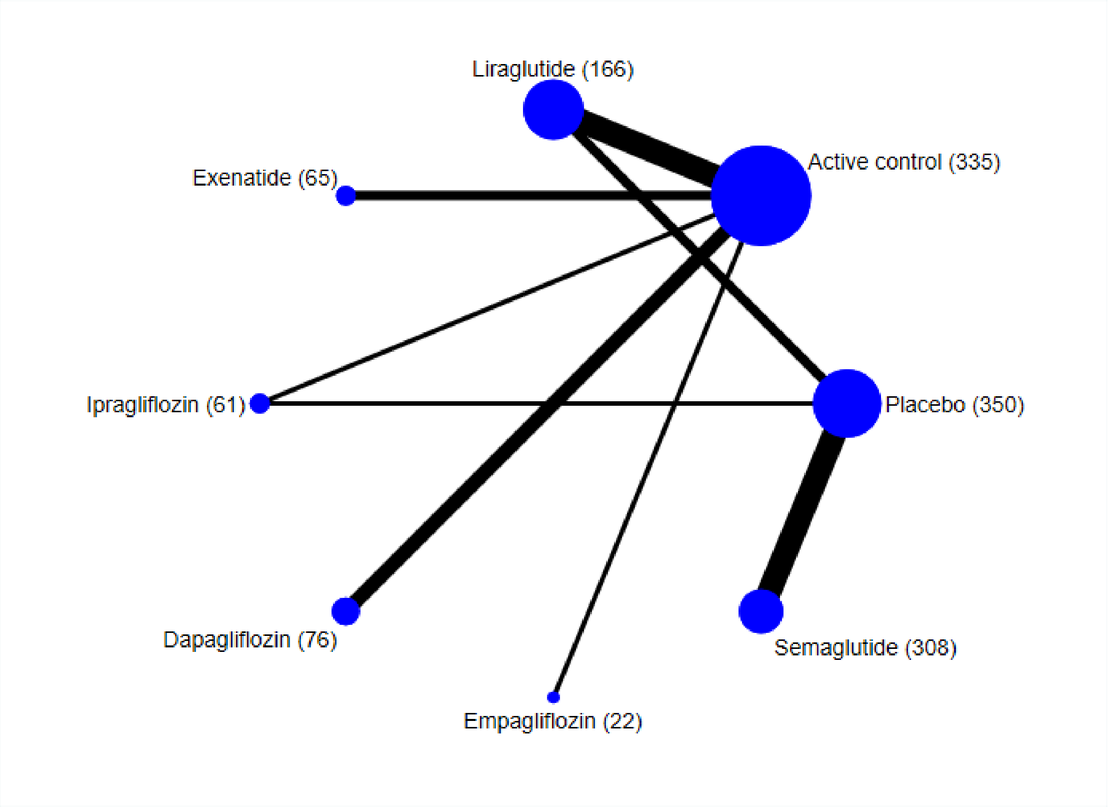

Figure 13 Network plot for diastolic blood pressure (DBP)

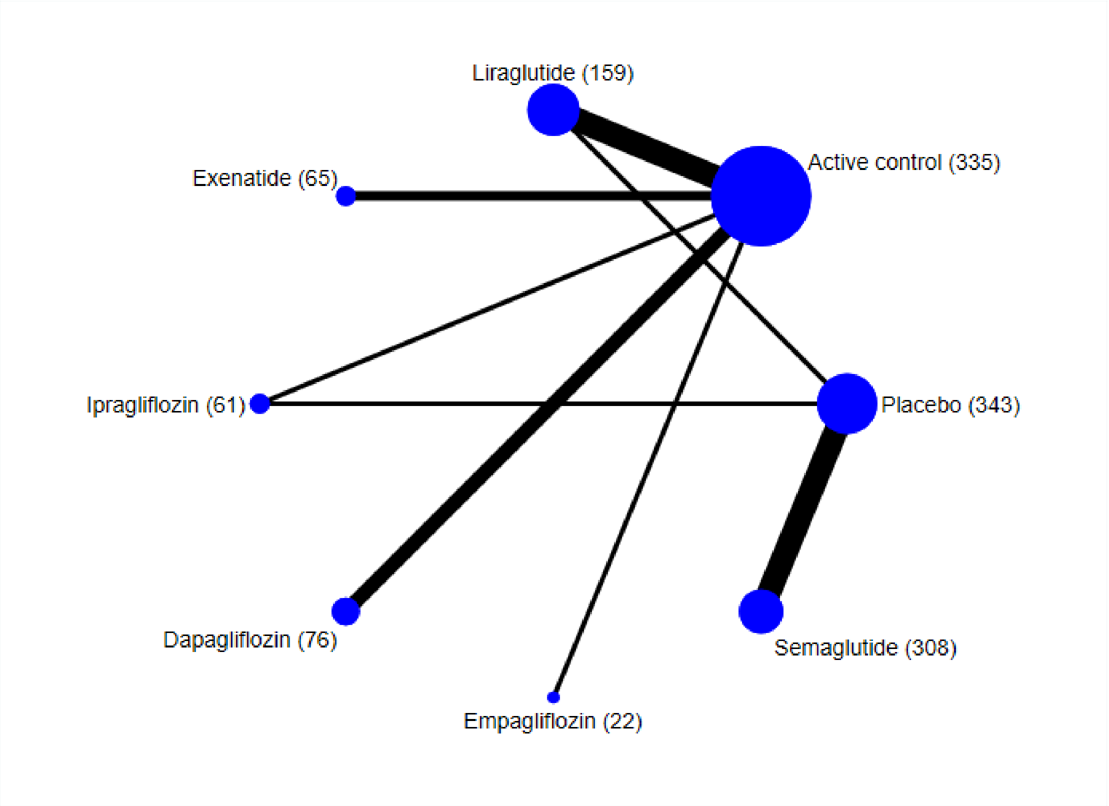

Figure 14 Network plot for blood lipids [total cholesterol(TC)

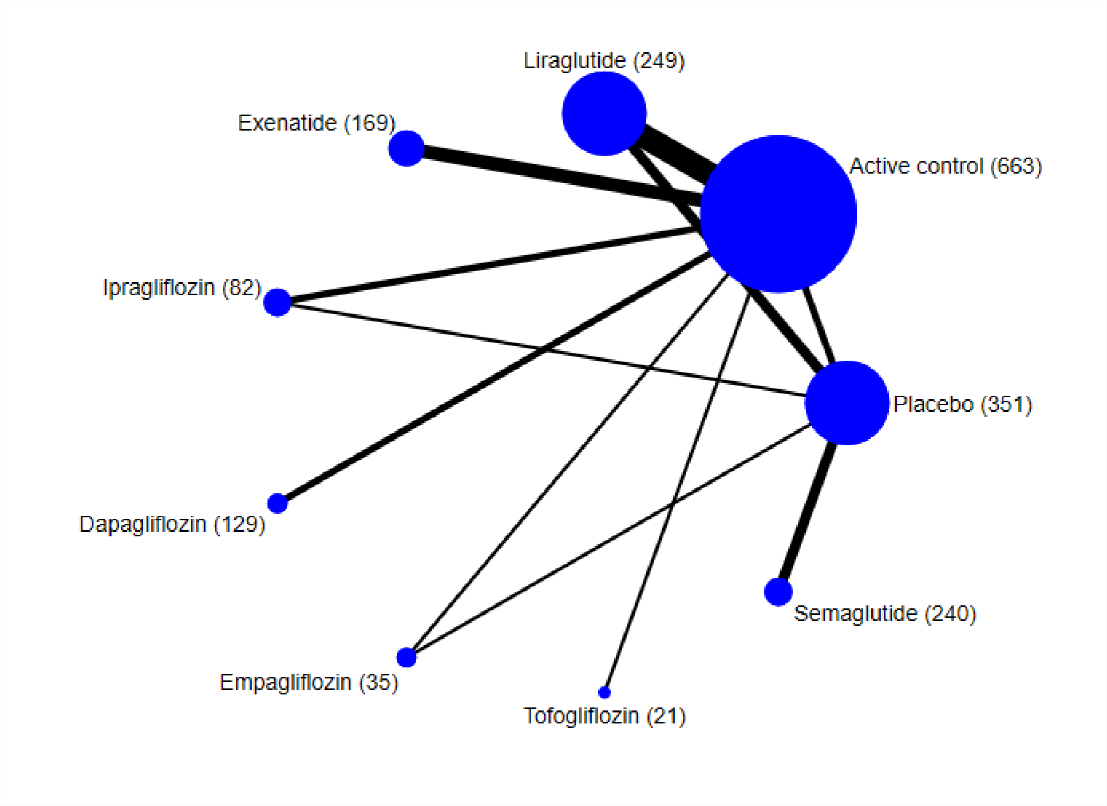

Figure 15 Network plot for triglycerides (TG)

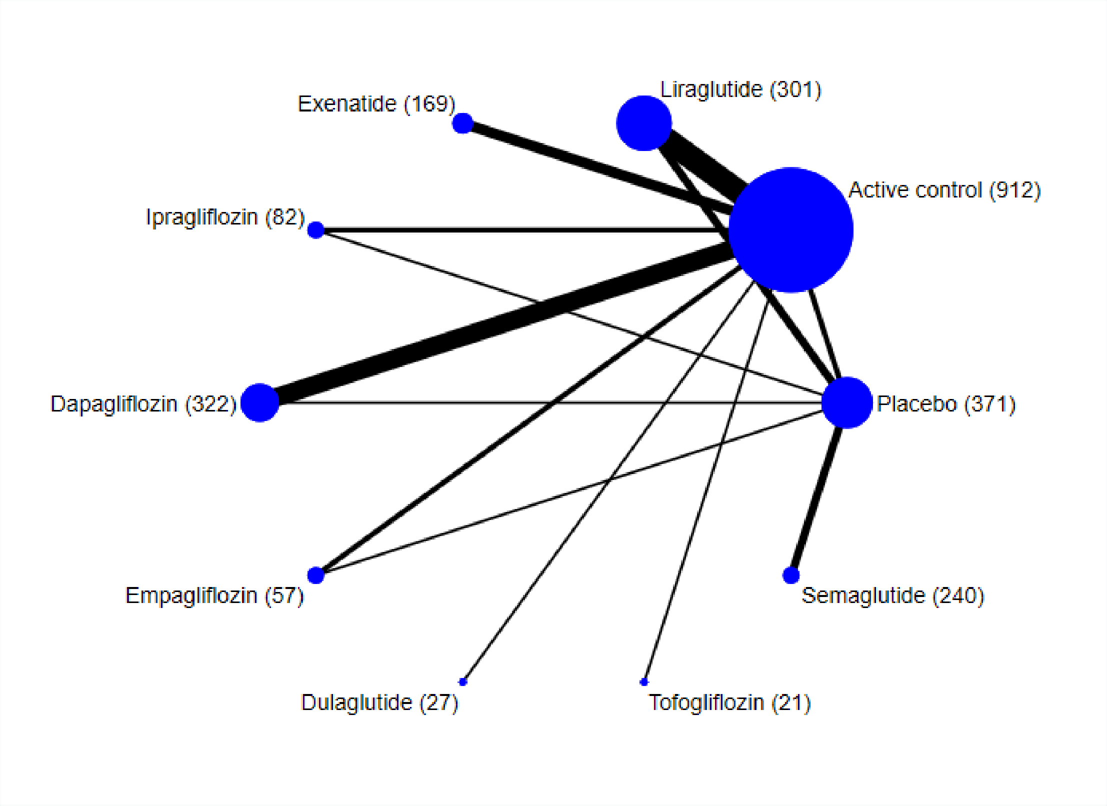

Figure 16 Network plot for high density lipoprotein-cholesterol (HDL-C)

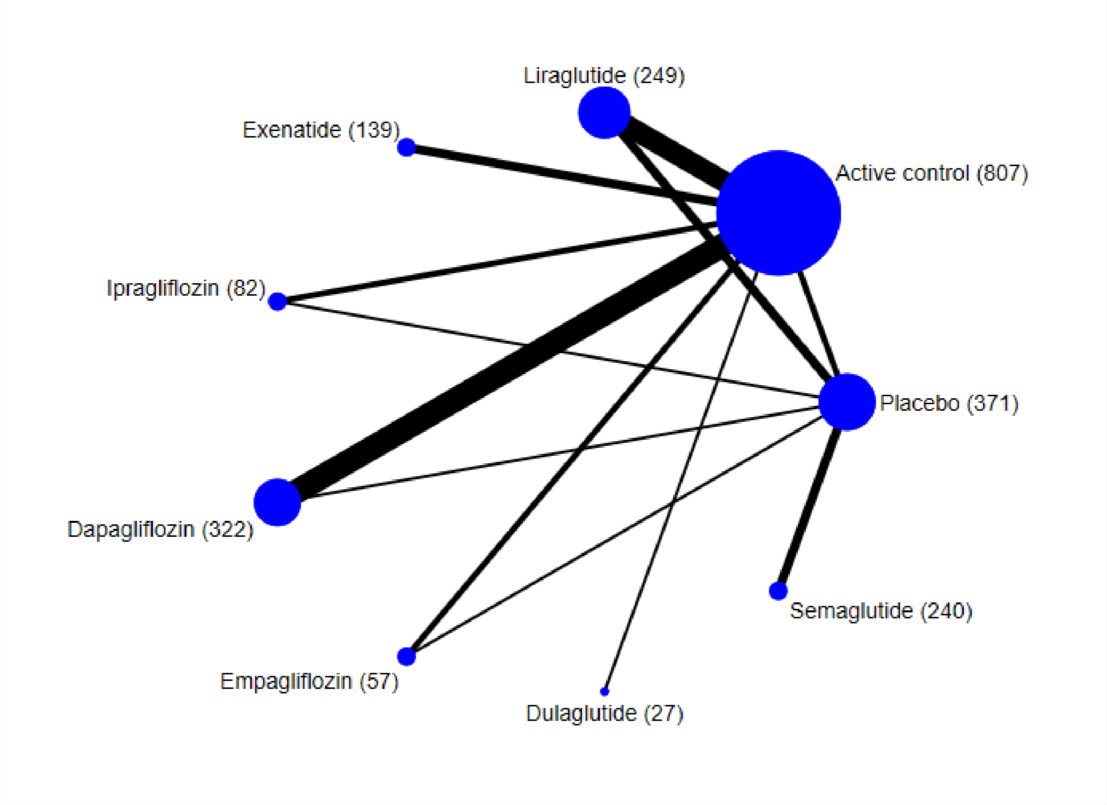

Figure 17 Network plot for low density lipoprotein-cholesterol (LDL-C)

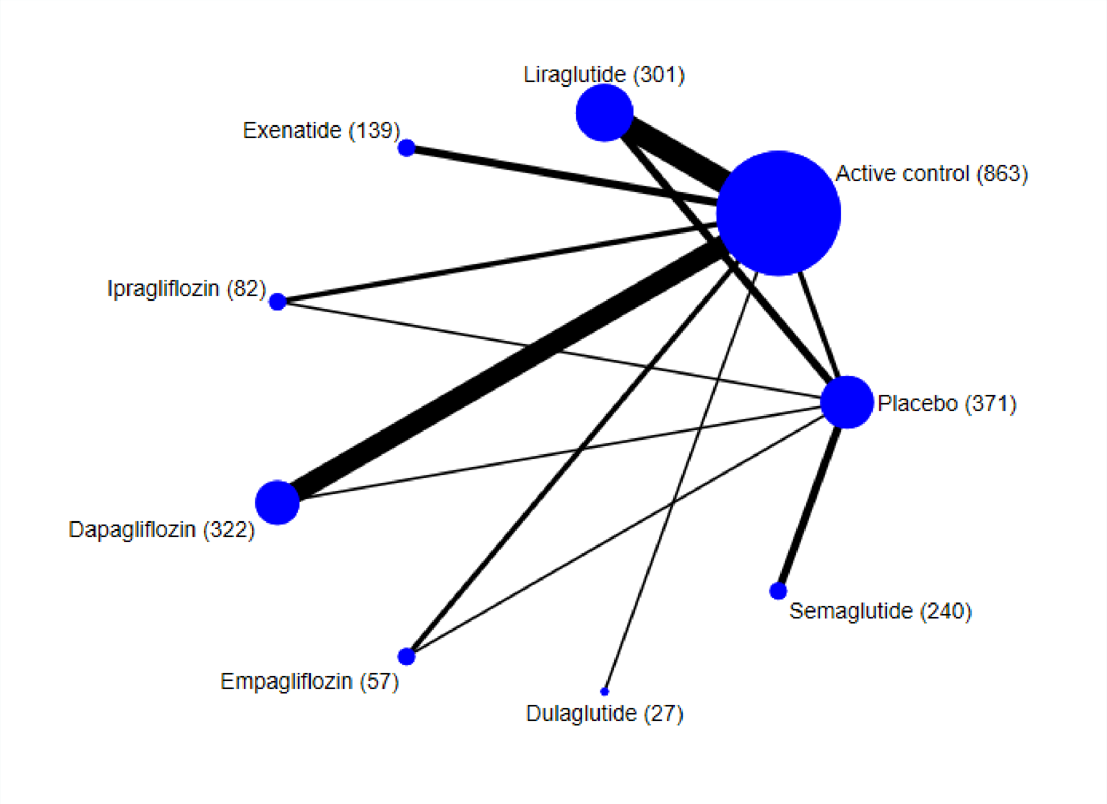

Figure 18 Network plot for serum adiponectin

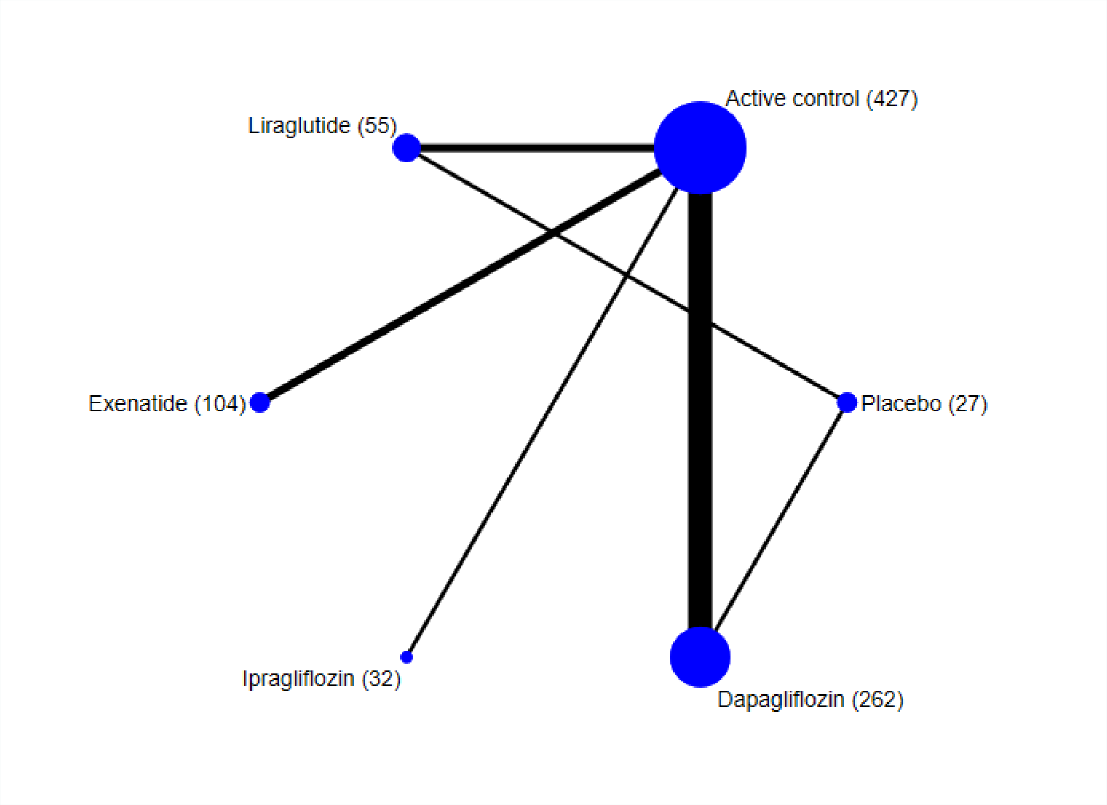

Figure 19 Network plot for fasting blood glucose (FBG)

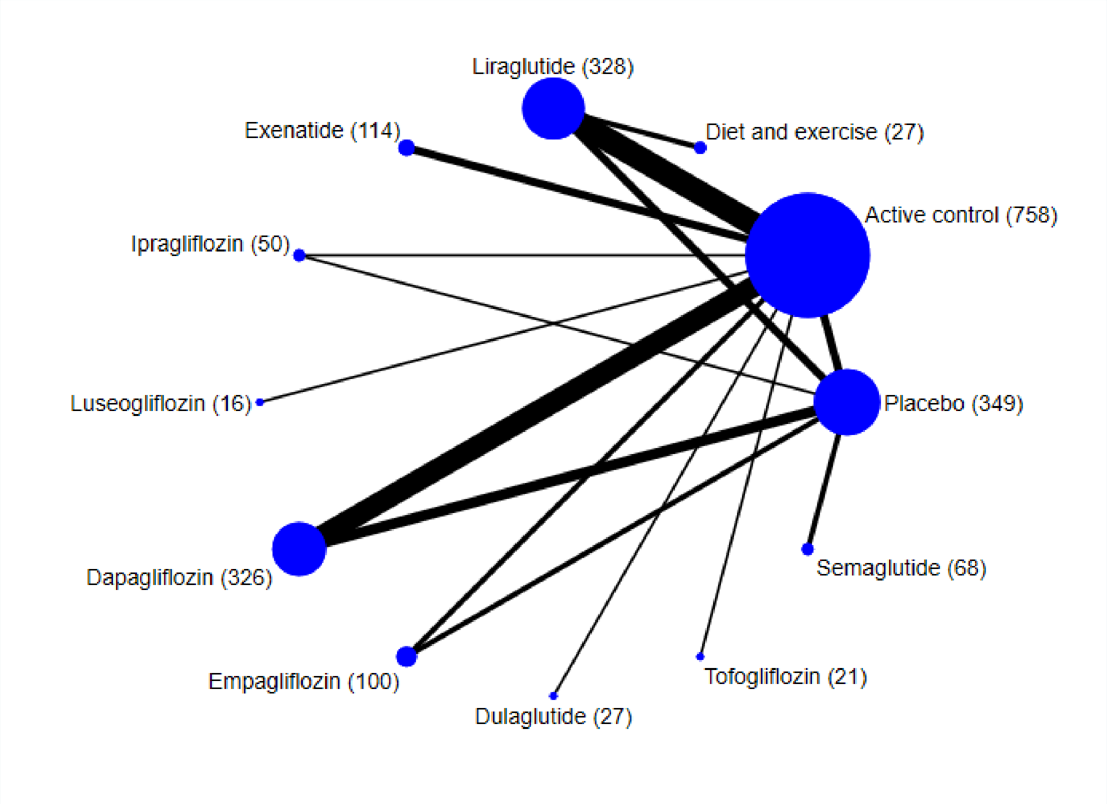

Figure 20 Network plot for postprandial blood glucose (PBG)

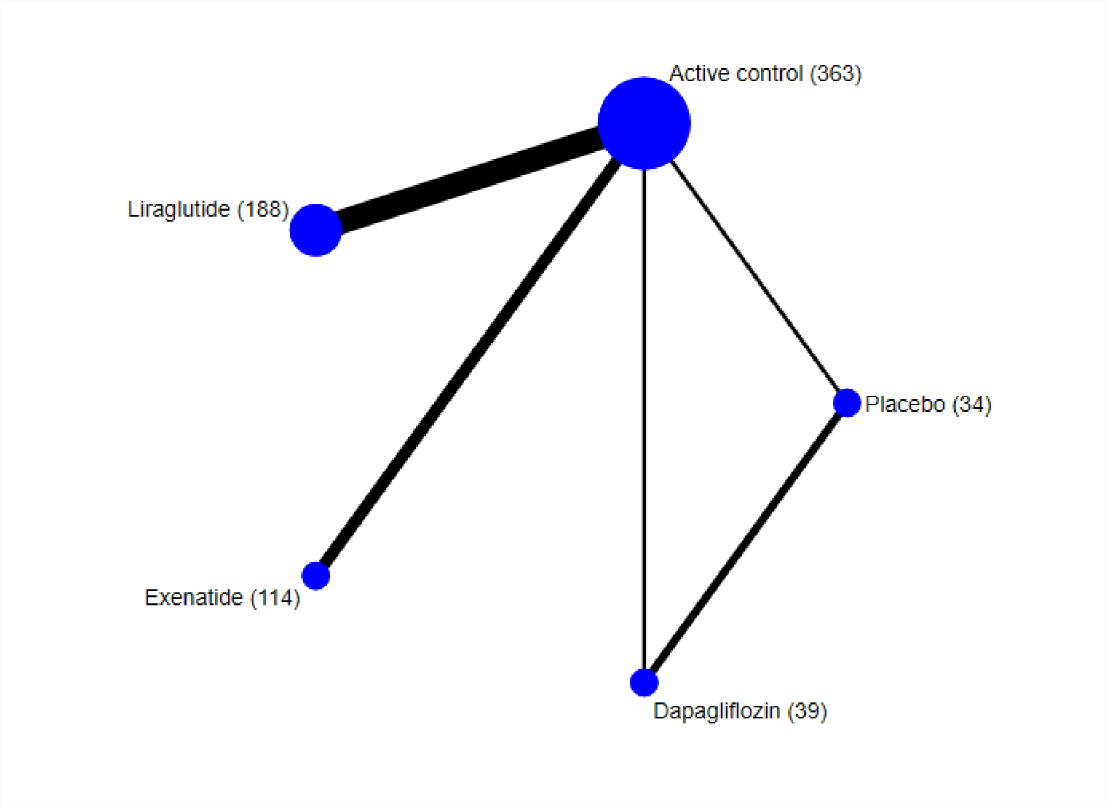

Figure 21 Network plot for glycosylated hemoglobin (HbA1c)

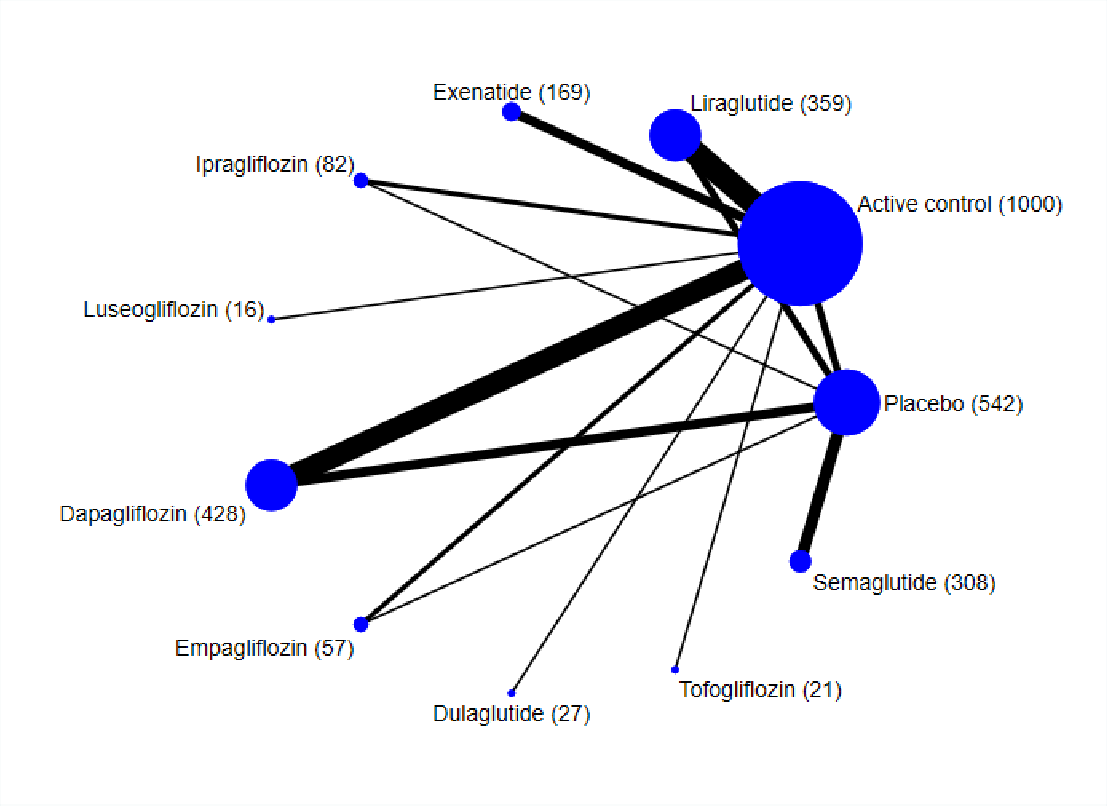

Figure 22 Network plot for glucose and homeostasis model assessment (HOMA-IR)

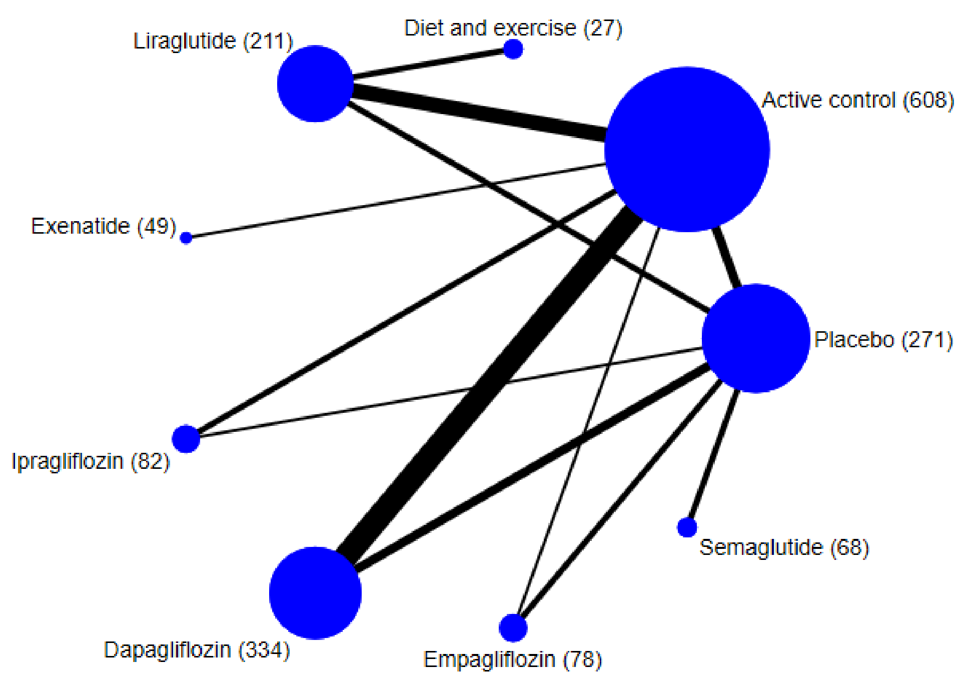

Appendix 7 Evaluations of network inconsistency

	Global consistency	
**Outcome**	**Chi square**	**P value**
Alanine Aminotransferase (ALT)	2.39	0.9354
Aspartate Aminotransferase (AST)	5.72	0.5722
γ-Glutamyl Transferase (GGT)	0.05	0.8317
Subcutaneous Adipose Tissue (SAT)	1.88	0.8654
Visceral Adipose Tissue (VAT)	9.26	0.2345
Liver Fat Fraction (LFF)	1.4	0.2363
Controlled Attenuation Parameter (CAP)	0.15	0.7017
Liver Stiffness Measurement (LSM)	0	0.9892
Body Weight (BW)	1.78	0.9945
Body Mass Index (BMI)	1.23	0.9903
Waist Circumference (WC)	1	0.9624
Systobic Blood Pressure (SBP)	0.03	0.863
Diastolic Blood Pressure (DBP)	0.45	0.5013
Blood Lipids [Total Cholesterol(TC)	4.4	0.355
Triglycerides (TG)	1.77	0.9398
High Density Lipoprotein-Cholesterol (HDL-C)	10.45	0.1069
Low Density Lipoprotein-Cholesterol (LDL-C)	4.62	0.5933
Adiponectin	1.76	0.1841
Fasting Blood Glucose (FBG)	14.61	0.1022
Postprandial Blood Glucose (PBG)	0.65	0.4187
Glycosylated Hemoglobin (HbA1C)	7.1	0.5256
Glucose And Homeostasis Model Assessment (HOMA-IR)	15.78	0.0456

Appendix 8 Direct, indirect and network treatment estimates

Table 1 Alanine aminotransferase (ALT)

Intervention		Comparator	Direct estimate		Indirect estimate		Incoherence	Network estimate
			Mean	SD	Mean	SD	p value	MD (95%CI)
Active control	v	Placebo	-1.47	6.85	-4.96	4.63	0.67	-3.81 (-11.16,3.54)
Liraglutide	v	Placebo	-11.62	5.89	-4.95	5.96	0.43	-8.30 (-16.16,-0.43)
Ipragliflozin	v	Placebo	-3.10	9.12	-13.37	9.30	0.43	-8.09 (-20.72,4.54)
Dapagliflozin	v	Placebo	-12.34	6.72	-8.20	5.79	0.64	-9.94 (-18.42,-1.46)
Empagliflozin	v	Placebo	-2.79	7.08	-13.05	12.14	0.47	-5.37 (-17.17,6.43)
Liraglutide	v	Active control	-3.47	3.34	-11.69	9.00	0.39	-4.49 (-10.38,1.41)
Exenatide	v	Active control	-5.85	4.81	7.69	250.16	0.96	-5.84 (-15.26,3.58)
Ipragliflozin	v	Active control	-8.56	8.37	1.71	9.96	0.43	-4.28 (-16.67,8.12)
Luseogliflozin	v	Active control	4.50	10.07	7.62	820.11	1.00	4.50 (-15.23,24.23)
Dapagliflozin	v	Active control	-5.33	3.82	-9.47	8.01	0.64	-6.13 (-12.82,0.57)
Empagliflozin	v	Active control	-3.33	7.99	1.34	10.23	0.72	-1.56 (-13.65,10.53)
Dulaglutide	v	Active control	-12.50	11.61	8.10	1698.74	0.99	-6.13 (-12.82,0.57)
Tofogliflozin	v	Active control	10.50	12.70	7.10	1784.77	1.00	10.50 (-14.40,35.40)
Liraglutide	v	Diet and exercise	10.88	11.60	-16.15	1573.51	0.99	-4.49 (-10.38,1.41)

Table 2 Aspartate aminotransferase (AST)

Intervention		Comparator	Direct estimate		Indirect estimate		Incoherence	Network estimate
			**Mean**	**SD**	**Mean**	**SD**	**p value**	**MD (95%CI)**
Active control	v	Placebo	-1.38	4.08	-5.36	2.86	0.43	-4.04 (-8.58,0.50)
Liraglutide	v	Placebo	-4.56	3.59	-4.69	3.65	0.98	-4.60 (-9.48,0.29)
Ipragliflozin	v	Placebo	-3.40	5.25	-11.74	5.89	0.29	-7.08(-14.76,0.60)
Dapagliflozin	v	Placebo	-13.67	3.72	-1.62	3.23	0.02	-6.70(-12.03,-1.37)
Empagliflozin	v	Placebo	-3.19	4.04	-11.75	7.50	0.31	-5.08 (-12.07,1.92)
Liraglutide	v	Active control	-0.69	1.97	0.38	5.41	0.85	-0.55 (-4.12,3.02)
Exenatide	v	Active control	-4.46	2.84	8.22	234.91	0.96	-4.45 (-10.03,1.12)
Ipragliflozin	v	Active control	-6.88	5.34	1.46	5.80	0.29	-3.04 (-10.71,4.64)
Dapagliflozin	v	Active control	-0.45	2.22	-12.50	4.43	0.02	-2.66 (-6.97,1.65)
Empagliflozin	v	Active control	-3.89	4.88	3.02	5.81	0.36	-1.03 (-8.34,6.28)
Dulaglutide	v	Active control	-9.30	7.23	8.44	1157.99	0.99	-9.30 (-23.47,4.87)
Tofogliflozin	v	Active control	12.10	7.34	7.48	1043.61	1.00	12.10 (-2.29,26.49)
Liraglutide	v	Diet and exercise	6.65	6.48	-9.12	860.76	0.99	6.65 (-6.05,19.34)

Table 3 γ-glutamyl transferase (GGT)

Intervention		Comparator	Direct estimate		Indirect estimate		Incoherence	Network estimate
			**Mean**	**SD**	**Mean**	**SD**	**p value**	**MD (95%CI)**
Liraglutide	v	Placebo	-9.59	7.90	-2.03	12.33	0.61	-7.29(-19.88,5.30)
Ipragliflozin	v	Placebo	-9.30	9.91	-13.08	14.79	0.83	-10.37 (-26.04,5.31)
Liraglutide	v	Active control	-0.64	6.40	-8.18	16.62	0.67	-1.69 (-12.94,9.56)
Exenatide	v	Active control	-1.69	5.97	11.75	443.74	0.98	-1.69 (-13.38,10.01)
Ipragliflozin	v	Active control	-6.29	11.62	-2.51	13.50	0.83	-4.76 (-21.65,12.12)
Dapagliflozin	v	Active control	-8.22	4.36	11.87	512.95	0.97	-8.22 (-16.75,0.32)
Empagliflozin	v	Active control	-11.00	15.06	11.73	2338.13	0.99	-11.00 (-40.52,18.52)
Dulaglutide	v	Active control	-13.10	11.08	11.71	1279.68	0.99	-13.10 (-34.82,8.63)
Tofogliflozin	v	Active control	29.40	13.83	9.73	1997.59	0.99	29.40 (2.29,56.50)

Table 4 Subcutaneous adipose tissue (SAT)

Intervention		Comparator	Direct estimate		Indirect estimate		Incoherence	Network estimate
			**Mean**	**SD**	**Mean**	**SD**	**p value**	**MD (95%CI)**
Active control	v	Placebo	-2.60	6.67	7.80	14.06	0.57	0.10 (-0.01,0.21)
Liraglutide	v	Placebo	-32.25	8.82	-29.03	7.13	0.77	-30.27(-41.54,-19.01)
Ipragliflozin	v	Placebo	-7.90	1.86	-21.90	27.88	0.62	-7.96 (-11.60,-4.33)
Dapagliflozin	v	Placebo	-0.26	0.05	-14.21	14.63	0.34	-0.26 (-0.36,-0.17)
Liraglutide	v	Active control	-27.68	6.50	-42.76	15.17	0.38	-30.37 (-41.63,-19.11)
Exenatide	v	Active control	-31.03	12.62	-0.63	3363.97	0.99	-31.03 (-55.76,-6.30)
Ipragliflozin	v	Active control	-22.00	27.88	-8.00	1.86	0.62	-8.06 (-11.70,-4.42)
Dapagliflozin	v	Active control	-0.34	0.07	-0.44	0.17	0.63	-0.36 (-0.48,-0.24)

Table 5 Visceral adipose tissue (VAT)

Intervention		Comparator	Direct estimate		Indirect estimate		Incoherence	Network estimate
			**Mean**	**SD**	**Mean**	**SD**	**p value**	**MD (95%CI)**
Active control	v	Placebo	-4.24	7.24	-7.24	10.52	0.82	4.99 (-5.85,15.84)
Liraglutide	v	Placebo	-43.51	8.56	-15.83	8.65	0.02	**-30.12 (-45.36,-14.89)**
Ipragliflozin	v	Placebo	-33.20	9.13	21.54	23.80	0.03	**-25.13 (-44.49,-5.76)**
Dapagliflozin	v	Placebo	-0.23	0.06	-26.86	5.17	0.00	-6.96 (-18.37,4.46)
Empagliflozin	v	Placebo	-8.66	10.56	-30.53	29.13	0.49	-11.45 (-30.12,7.22)
Liraglutide	v	Active control	-22.22	6.07	-57.93	20.09	0.09	-25.13 (-38.14,-12.12)
Exenatide	v	Active control	-35.27	16.47	9.49	3352.63	0.99	-35.27 (-67.55,-2.99)
Ipragliflozin	v	Active control	24.00	23.24	-30.74	10.47	0.03	-20.13 (-42.06,1.80)
Dapagliflozin	v	Active control	-5.29	4.99	15.04	11.38	0.10	-1.96 (-11.76,7.84)
Empagliflozin	v	Active control	-14.30	14.76	1.30	14.62	0.45	-6.45 (-26.29,13.39)

Table 6 Liver fat fraction (LFF)

Intervention		Comparator	Direct estimate		Indirect estimate		Incoherence	Network estimate
			**Mean**	**SD**	**Mean**	**SD**	**p value**	**MD (95%CI)**
Active control	v	Placebo	-2.56	0.86	0.72	2.77	0.24	**-2.34 (-4.36,-0.33)**
Dapagliflozin	v	Placebo	-0.95	0.80	6.37	96.06	0.94	-0.95 (-2.52,0.62)
Liraglutide	v	Active control	-2.82	0.62	4.42	92.68	0.94	-4.21 (-6.69,-1.73)
Dapagliflozin	v	Active control	0.92	1.06	4.19	2.55	0.24	1.39 (-0.78,3.56)
Tofogliflozin	v	Active control	3.42	2.07	4.51	397.38	1.00	3.42 (-0.64,7.48)
Liraglutide	v	Diet and exercise	1.36	2.93	-10.35	456.16	0.98	1.36 (-4.37,7.10)

Table 7 Controlled attenuation parameter (CAP)

Intervention		Comparator	Direct estimate		Indirect estimate		Incoherence	Network estimate
			**Mean**	**SD**	**Mean**	**SD**	**p value**	**MD (95%CI)**
Active control	v	Placebo	-11.44	14.87	4.09	37.54	0.70	-9.26 (-33.78,15.26)
Empagliflozin	v	Placebo	-9.04	9.10	43.76	2427.30	0.98	-9.04 (-26.88,8.81)
Dapagliflozin	v	Active control	-29.60	12.41	23.20	2426.15	0.98	-29.60 (-53.91,-5.28)
Empagliflozin	v	Active control	-1.72	14.54	13.77	37.95	0.70	0.22 (-23.92,24.37)

Table 8 Liver stiffness measurement (LSM)

Intervention		Comparator	Direct estimate		Indirect estimate		Incoherence	Network estimate
			**Mean**	**SD**	**Mean**	**SD**	**p value**	**MD (95%CI)**
Active control	v	Placebo	0.26	0.62	0.28	1.29	0.99	0.26 (-0.77,1.30)
Empagliflozin	v	Placebo	-0.49	0.32	-0.97	62.98	0.99	-0.49 (-1.11,0.12)
Dapagliflozin	v	Active control	-1.93	1.34	-0.22	322.69	1.00	-1.93 (-4.56,0.70)
Empagliflozin	v	Active control	-0.76	0.57	-0.74	1.35	0.99	-0.76 (-1.76,0.25)
Dulaglutide	v	Active control	-1.31	0.89	-0.48	191.24	1.00	-1.31 (-3.04,0.43)
Tofogliflozin	v	Active control	0.20	0.45	-0.54	68.20	0.99	0.20 (-0.68,1.08)

Table 9 Body weight (BW)

Intervention		Comparator	Direct estimate		Indirect estimate		Incoherence	Network estimate
			**Mean**	**SD**	**Mean**	**SD**	**p value**	**MD (95%CI)**
Active control	v	Placebo	0.36	1.88	0.03	1.56	0.89	0.16 (-2.16,2.47)
Liraglutide	v	Placebo	-5.17	1.74	-1.92	1.97	0.22	-3.75 (-6.34,-1.15)
Ipragliflozin	v	Placebo	-2.00	2.89	-4.88	3.84	0.55	-3.04 (-7.54,1.47)
Dapagliflozin	v	Placebo	-2.87	1.48	-4.80	2.18	0.47	-3.48 (-5.88,-1.08)
Empagliflozin	v	Placebo	-2.31	2.87	-3.29	4.49	0.85	-2.60 (-7.31,2.12)
Liraglutide	v	Active control	-3.29	1.20	-6.76	2.58	0.22	-3.90 (-6.05,-1.75)
Exenatide	v	Active control	-4.56	1.48	-0.15	101.53	0.97	-4.56 (-7.47,-1.65)
Ipragliflozin	v	Active control	-4.85	3.64	-1.97	3.14	0.55	-3.19 (-7.84,1.45)
Dapagliflozin	v	Active control	-3.95	1.36	-2.70	2.35	0.64	-3.64 (-5.92,-1.35)
Empagliflozin	v	Active control	-3.41	3.17	-1.79	3.82	0.74	-2.75 (-7.53,2.02)
Dulaglutide	v	Active control	-2.30	2.99	-0.22	191.82	0.99	-2.30 (-8.16,3.56)
Tofogliflozin	v	Active control	1.44	2.99	-0.39	173.76	0.99	1.44 (-4.43,7.31)
Liraglutide	v	Diet and exercise	0.25	2.16	-7.49	121.93	0.95	0.25 (-3.99,4.49)

Table 10 Body mass index (BMI)

Intervention		Comparator	Direct estimate		Indirect estimate		Incoherence	Network estimate
			**Mean**	**SD**	**Mean**	**SD**	**p value**	**MD (95%CI)**
Active control	v	Placebo	0.01	0.80	-0.27	0.55	0.77	-0.18 (-1.06,0.69)
Liraglutide	v	Placebo	-1.79	0.59	-1.01	0.74	0.41	-1.49 (-2.39,-0.59)
Ipragliflozin	v	Placebo	-0.80	0.91	-2.05	1.73	0.52	-1.07 (-2.64,0.50)
Dapagliflozin	v	Placebo	-1.16	0.71	-1.07	0.80	0.93	-1.13 (-2.14,-0.11)
Empagliflozin	v	Placebo	-0.88	0.85	-1.52	1.32	0.69	-1.07 (-2.45,0.31)
Liraglutide	v	Active control	-1.22	0.37	-1.93	0.97	0.50	-1.30 (-1.97,-0.63)
Exenatide	v	Active control	-1.67	0.47	0.45	32.99	0.95	-1.67 (-2.59,-0.76)
Ipragliflozin	v	Active control	-1.80	1.67	-0.55	1.02	0.52	-0.88 (-2.59,0.82)
Luseogliflozin	v	Active control	-0.65	0.92	0.37	29.46	0.97	-0.65 (-2.44,1.15)
Dapagliflozin	v	Active control	-0.91	0.60	-1.00	0.88	0.93	-0.94 (-1.89,0.01)
Empagliflozin	v	Active control	-1.24	0.92	-0.32	1.17	0.54	-0.88 (-2.29,0.52)
Dulaglutide	v	Active control	-0.82	0.97	0.40	79.76	0.99	-0.82 (-2.72,1.08)
Liraglutide	v	Diet and exercise	0.20	0.69	-2.98	42.51	0.94	0.20 (-1.15,1.56)

Table 11 Waist circumference (WC)

Intervention		Comparator	Direct estimate		Indirect estimate		Incoherence	Network estimate
			**Mean**	**SD**	**Mean**	**SD**	**p value**	**MD (95%CI)**
Active control	v	Placebo	-0.42	2.03	-1.43	2.14	0.74	-0.90 (-3.70,1.90)
Liraglutide	v	Placebo	-5.53	1.77	-2.52	2.50	0.33	-4.53 (-7.37,-1.69)
Dapagliflozin	v	Placebo	-2.29	1.62	-4.98	3.42	0.48	-2.78 (-5.61,0.06)
Liraglutide	v	Active control	-3.28	1.26	-5.87	3.20	0.45	-3.63 (-5.90,-1.35)
Exenatide	v	Active control	-5.22	1.56	1.86	103.53	0.95	-5.22 (-8.28,-2.15)
Dapagliflozin	v	Active control	-2.78	2.03	-0.21	2.77	0.46	-1.88 (-5.04,1.29)
Liraglutide	v	Diet and exercise	-1.05	2.10	-9.06	168.78	0.96	-1.05 (-5.18,3.07)

Table 12 Systobic blood pressure (SBP)

Intervention		Comparator	Direct estimate		Indirect estimate		Incoherence	Network estimate
			**Mean**	**SD**	**Mean**	**SD**	**p value**	**MD (95%CI)**
Liraglutide	v	Placebo	-3.71	3.31	-2.94	2.92	0.86	-3.27 (-7.45,0.91)
Ipragliflozin	v	Placebo	-3.80	1.68	-4.56	4.08	0.86	-3.90 (-6.79,-1.01)
Liraglutide	v	Active control	-2.24	1.67	-3.01	4.09	0.86	-2.36 (-5.35,0.63)
Exenatide	v	Active control	-2.20	1.93	1.68	379.02	0.99	-2.20 (-5.98,1.58)
Ipragliflozin	v	Active control	-3.10	1.71	-2.34	4.07	0.86	-2.99 (-5.94,-0.05)
Dapagliflozin	v	Active control	-2.71	2.52	1.92	473.04	0.99	-2.71 (-7.65,2.24)
Empagliflozin	v	Active control	6.00	4.72	1.53	888.49	1.00	6.00 (-3.25,15.25)

Table 13 Diastolic blood pressure (DBP)

Intervention		Comparator	Direct estimate		Indirect estimate		Incoherence	Network estimate
			Mean	SD	Mean	SD	p value	MD (95%CI)
Liraglutide	v	Placebo	-1.80	3.39	0.76	1.72	0.50	0.24 (-2.76,3.24)
Ipragliflozin	v	Placebo	0.50	0.98	-2.04	3.66	0.50	0.33 (-1.50,2.17)
Liraglutide	v	Active control	0.56	1.18	-2.00	3.61	0.50	0.31 (-1.87,2.50)
Exenatide	v	Active control	-0.70	1.24	0.12	280.24	1.00	-0.70 (-3.13,1.73)
Ipragliflozin	v	Active control	0.30	0.79	2.85	3.71	0.50	0.40 (-1.08,1.89)
Dapagliflozin	v	Active control	-1.52	1.55	-0.37	303.54	1.00	-1.52 (-4.56,1.52)
Empagliflozin	v	Active control	2.00	3.05	0.05	601.26	1.00	2.00 (-3.97,7.97)

Table 14 Blood lipids [total cholesterol (TC)

Intervention		Comparator	Direct estimate		Indirect estimate		Incoherence	Network estimate
			Mean	SD	Mean	SD	p value	MD (95%CI)
Active control	v	Placebo	-0.24	0.23	-0.13	0.20	0.71	-0.17 (-0.47,0.12)
Liraglutide	v	Placebo	-0.43	0.19	0.07	0.24	0.10	-0.24 (-0.55,0.06)
Ipragliflozin	v	Placebo	0.24	0.22	-0.17	0.24	0.20	0.05 (-0.28,0.38)
Empagliflozin	v	Placebo	-0.14	0.31	-0.57	0.66	0.58	-0.23 (-0.76,0.30)
Liraglutide	v	Active control	-0.03	0.13	-0.35	0.32	0.35	-0.07 (-0.30,0.16)
Exenatide	v	Active control	-0.16	0.14	0.34	21.50	0.98	-0.16 (-0.44,0.12)
Ipragliflozin	v	Active control	0.09	0.18	0.53	0.28	0.18	0.22 (-0.09,0.53)
Dapagliflozin	v	Active control	0.47	0.19	0.35	22.21	1.00	0.47 (0.09,0.85)
Empagliflozin	v	Active control	-0.13	0.31	0.29	0.67	0.58	-0.06 (-0.58,0.46)
Tofogliflozin	v	Active control	0.16	0.38	0.34	60.99	1.00	0.16 (-0.59,0.91)

Table 15 Triglycerides (TG)

Intervention		Comparator	Direct estimate		Indirect estimate		Incoherence	Network estimate
			Mean	SD	Mean	SD	p value	MD (95%CI)
Active control	v	Placebo	-0.06	0.17	-0.04	0.12	0.93	-0.05 (-0.24,0.15)
Liraglutide	v	Placebo	-0.35	0.16	-0.04	0.17	0.18	-0.20 (-0.43,0.03)
Ipragliflozin	v	Placebo	-0.25	0.14	-0.28	0.19	0.88	-0.26 (-0.48,-0.04)
Dapagliflozin	v	Placebo	-0.12	0.18	-0.32	0.15	0.39	-0.23 (-0.46,-0.01)
Empagliflozin	v	Placebo	-0.04	0.36	-0.13	0.35	0.86	-0.09 (-0.60,0.42)
Liraglutide	v	Active control	-0.13	0.09	-0.36	0.26	0.39	-0.15 (-0.33,0.02)
Exenatide	v	Active control	-0.17	0.09	0.11	13.41	0.98	-0.17 (-0.35,0.02)
Ipragliflozin	v	Active control	-0.23	0.15	-0.19	0.18	0.88	-0.21 (-0.44,0.01)
Dapagliflozin	v	Active control	-0.22	0.10	-0.02	0.21	0.39	-0.19 (-0.36,-0.02)
Empagliflozin	v	Active control	-0.05	0.27	0.02	0.62	0.91	-0.04 (-0.53,0.45)
Dulaglutide	v	Active control	-0.30	1.23	0.10	271.42	1.00	-0.19 (-0.36,-0.02)
Tofogliflozin	v	Active control	0.56	0.31	-0.08	49.12	0.99	0.56 (-0.05,1.17)

Table 16 High density lipoprotein-cholesterol (HDL-C)

Intervention		Comparator	Direct estimate		Indirect estimate		Incoherence	Network estimate
			**Mean**	**SD**	**Mean**	**SD**	**p value**	**MD (95%CI)**
Active control	v	Placebo	0.15	0.06	0.05	0.04	0.16	0.08 (0.02,0.14)
Liraglutide	v	Placebo	0.12	0.04	0.02	0.05	0.10	0.08 (0.02,0.15)
Ipragliflozin	v	Placebo	0.06	0.03	0.13	0.07	0.35	0.08 (0.01,0.14)
Dapagliflozin	v	Placebo	0.05	0.07	0.17	0.04	0.13	0.14 (0.06,0.21)
Empagliflozin	v	Placebo	0.02	0.06	0.07	0.10	0.61	0.03 (-0.07,0.14)
Liraglutide	v	Active control	-0.02	0.03	0.08	0.06	0.11	0.00 (-0.05,0.06)
Exenatide	v	Active control	-0.01	0.04	-0.16	8.44	0.99	-0.01 (-0.08,0.07)
Ipragliflozin	v	Active control	0.04	0.06	-0.03	0.05	0.35	-0.00 (-0.08,0.07)
Dapagliflozin	v	Active control	0.07	0.03	-0.05	0.07	0.13	0.06 (0.01,0.11)
Empagliflozin	v	Active control	-0.04	0.06	-0.07	0.13	0.83	-0.04 (-0.15,0.06)
Dulaglutide	v	Active control	-0.10	0.28	-0.15	64.53	1.00	-0.10 (-0.66,0.46)

Table 17 Low density lipoprotein-cholesterol (LDL-C)

Intervention		Comparator	Direct estimate		Indirect estimate		Incoherence	Network estimate
			**Mean**	**SD**	**Mean**	**SD**	**p value**	**MD (95%CI)**
Active control	v	Placebo	-0.03	0.24	-0.09	0.19	0.83	-0.07 (-0.36,0.22)
Liraglutide	v	Placebo	-0.14	0.21	0.11	0.24	0.43	-0.03 (-0.34,0.28)
Ipragliflozin	v	Placebo	0.29	0.29	-0.13	0.29	0.31	0.08 (-0.32,0.49)
Dapagliflozin	v	Placebo	-0.06	0.36	0.01	0.21	0.85	-0.00 (-0.35,0.34)
Empagliflozin	v	Placebo	-0.11	0.32	-0.53	0.38	0.40	-0.28 (-0.76,0.19)
Liraglutide	v	Active control	0.10	0.12	-0.41	0.32	0.14	0.04 (-0.18,0.26)
Exenatide	v	Active control	-0.18	0.19	0.14	22.79	0.99	-0.18 (-0.55,0.19)
Ipragliflozin	v	Active control	0.00	0.24	0.43	0.33	0.31	0.15 (-0.24,0.53)
Dapagliflozin	v	Active control	0.07	0.13	-0.01	0.39	0.85	0.06 (-0.17,0.30)
Empagliflozin	v	Active control	-0.24	0.24	-0.05	0.67	0.79	-0.22 (-0.66,0.22)
Dulaglutide	v	Active control	0.20	1.12	0.13	239.23	1.00	0.20 (-2.00,2.40)

Table 18 Serum adiponectin

Intervention		Comparator	Direct estimate		Indirect estimate		Incoherence	Network estimate
			**Mean**	**SD**	**Mean**	**SD**	**p value**	**MD (95%CI)**
Liraglutide	v	Placebo	1.55	2.01	6.47	3.10	0.18	3.03 (-0.43,6.49)
Dapagliflozin	v	Placebo	-0.20	1.96	-5.03	3.11	0.19	-1.61 (-5.02,1.81)
Liraglutide	v	Active control	6.05	2.27	1.13	2.91	0.18	4.25 (0.56,7.94)
Exenatide	v	Active control	-0.37	1.44	2.32	91.35	0.98	-0.37 (-3.20,2.46)
Ipragliflozin	v	Active control	-5.96	2.43	2.26	327.44	0.98	-5.96 (-10.73,-1.19)
Dapagliflozin	v	Active control	-0.63	0.82	4.27	3.60	0.19	-0.39 (-2.02,1.25)

Table 19 Fasting blood glucose (FBG)

Intervention		Comparator	Direct estimate		Indirect estimate		Incoherence	Network estimate
			**Mean**	**SD**	**Mean**	**SD**	**p value**	**MD (95%CI)**
Active control	v	Placebo	-0.61	0.28	-0.15	0.21	0.19	-0.32 (-0.66,0.02)
Liraglutide	v	Placebo	-0.93	0.28	-0.60	0.31	0.41	-0.77 (-1.19,-0.35)
Ipragliflozin	v	Placebo	-0.09	0.29	-0.90	0.46	0.13	-0.33 (-0.84,0.18)
Dapagliflozin	v	Placebo	-0.63	0.22	-1.01	0.31	0.31	-0.75 (-1.12,-0.39)
Empagliflozin	v	Placebo	-0.03	0.26	0.74	0.71	0.30	0.06 (-0.44,0.55)
Liraglutide	v	Active control	-0.30	0.18	-1.07	0.40	0.09	-0.45 (-0.79,-0.11)
Exenatide	v	Active control	-0.04	0.23	0.65	39.46	0.99	-0.04 (-0.48,0.41)
Ipragliflozin	v	Active control	-0.50	0.42	0.31	0.34	0.13	-0.01 (-0.55,0.54)
Luseogliflozin	v	Active control	-2.00	7.36	0.64	1311.72	1.00	-2.00 (-16.42,12.42)
Dapagliflozin	v	Active control	-0.56	0.18	0.08	0.33	0.08	-0.43 (-0.76,-0.11)
Empagliflozin	v	Active control	0.94	0.51	0.12	0.34	0.19	0.38 (-0.19,0.95)
Dulaglutide	v	Active control	-1.00	4.49	0.68	1023.12	1.00	-1.00 (-9.80,7.80)
Tofogliflozin	v	Active control	0.99	0.81	0.59	152.08	1.00	0.99 (-0.59,2.58)
Liraglutide	v	Diet and exercise	-0.15	0.25	-1.55	24.60	0.96	-0.15 (-0.64,0.33)

Table 20 Postprandial blood glucose (PBG)

Intervention		Comparator	Direct estimate		Indirect estimate		Incoherence	Network estimate
			**Mean**	**SD**	**Mean**	**SD**	**p value**	**MD (95%CI)**
Active control	v	Placebo	-0.30	1.02	2.27	2.98	0.42	-0.00 (-1.82,1.81)
Dapagliflozin	v	Placebo	-2.14	0.78	-1.33	51.09	0.99	-2.14 (-3.67,-0.61)
Liraglutide	v	Active control	-1.70	0.48	-0.06	77.35	0.98	-1.70 (-2.63,-0.76)
Exenatide	v	Active control	-0.15	0.60	0.02	68.02	1.00	-0.15 (-1.33,1.04)
Dapagliflozin	v	Active control	-2.60	1.16	-0.02	2.83	0.42	-2.14 (-4.09,-0.19)

Table 21 Glycosylated hemoglobin (HbA1c)

Intervention		Comparator	Direct estimate		Indirect estimate		Incoherence	Network estimate
			Mean	SD	Mean	SD	p value	MD (95%CI)
Active control	v	Placebo	-0.34	0.20	-0.36	0.19	0.93	-0.35 (-0.61,-0.09)
Liraglutide	v	Placebo	-0.57	0.23	-0.44	0.21	0.68	-0.50 (-0.81,-0.19)
Ipragliflozin	v	Placebo	-0.30	0.29	-0.43	0.31	0.76	-0.36 (-0.77,0.04)
Dapagliflozin	v	Placebo	-0.61	0.21	-0.82	0.21	0.47	-0.72 (-1.01,-0.42)
Empagliflozin	v	Placebo	-0.42	0.34	0.28	0.39	0.18	-0.12 (-0.64,0.39)
Liraglutide	v	Active control	-0.13	0.13	-0.25	0.33	0.74	-0.15 (-0.38,0.09)
Exenatide	v	Active control	-0.06	0.16	0.70	21.46	0.97	-0.06 (-0.37,0.26)
Ipragliflozin	v	Active control	-0.06	0.27	0.07	0.32	0.76	-0.01 (-0.41,0.39)
Luseogliflozin	v	Active control	-0.70	0.36	0.70	35.14	0.97	-0.70 (-1.41,0.01)
Dapagliflozin	v	Active control	-0.41	0.12	-0.06	0.32	0.31	-0.36 (-0.58,-0.14)
Empagliflozin	v	Active control	0.42	0.26	-0.90	0.63	0.05	0.23 (-0.26,0.72)
Dulaglutide	v	Active control	-0.30	0.40	0.71	64.53	0.99	-0.30 (-1.08,0.48)
Tofogliflozin	v	Active control	0.29	0.28	0.69	11.06	0.97	0.29 (-0.26,0.85)

Table 22 Glucose and homeostasis model assessment (HOMA-IR)

Intervention		Comparator	Direct estimate		Indirect estimate		Incoherence	Network estimate
			**Mean**	**SD**	**Mean**	**SD**	**p value**	**MD (95%CI)**
Active control	v	Placebo	0.08	0.36	-0.99	0.47	0.08	-0.35 (-0.95,0.25)
Liraglutide	v	Placebo	-1.57	0.59	-1.01	0.61	0.50	-1.57 (-2.18,-0.96)
Ipragliflozin	v	Placebo	-0.60	0.58	-0.30	0.73	0.75	-0.60 (-1.01,-0.19)
Dapagliflozin	v	Placebo	-0.84	0.49	-0.87	0.51	0.97	-0.84 (-1.53,-0.15)
Empagliflozin	v	Placebo	-0.11	0.50	1.38	1.63	0.37	-0.11 (-0.49,0.27)
Liraglutide	v	Active control	-0.97	0.38	-0.65	1.19	0.79	-0.93 (-1.65,-0.22)
Exenatide	v	Active control	0.01	0.53	0.70	27.57	0.98	0.01 (-1.02,1.04)
Ipragliflozin	v	Active control	0.00	0.65	-0.29	0.67	0.75	-0.13 (-1.03,0.76)
Dapagliflozin	v	Active control	-0.55	0.31	-0.26	0.76	0.73	-0.51 (-1.05,0.04)
Empagliflozin	v	Active control	1.10	0.93	0.00	0.65	0.32	0.35 (-0.70,1.40)
Liraglutide	v	Diet and exercise	0.69	0.72	-2.56	102.26	0.98	-0.93 (-1.65,-0.22)

Appendix 9 Network meta-analysis treatment estimates

Table 1 Alanine aminotransferase (ALT)

The columns present the row drug class compared to the column drug class. The rows present the row drug class compared to the column drug class. The effect estimates are expressed as mean difference and 95% confidence interval. For example, the mean difference in ALT for diet and exercise therapy compared to semaglutide therapy is -4.47 U/L (95% confidence interval -30.55 U/L to 21.61 U/L). The mean difference in ALT for semaglutide therapy compared to diet and exercise therapy is the inverse (4.47 U/L (95% confidence interval -21.61 U/L to 30.55 U/L)).

Diet and exercise	4.47 (-21.61,30.55)	2.86 (-29.84,35.56)	9.24 (-15.04,33.52)	9.52 (-15.78,34.82)	10.87 (-11.85,33.60)	11.08 (-15.23,37.40)	13.80 (-12.31,39.91)	15.36 (-8.12,38.84)	19.86 (-10.81,50.53)	19.17 (-4.88,43.22)	25.86 (-8.37,60.09)
-4.47 (-30.55,21.61)	Semaglutide	-1.61 (-27.56,24.34)	4.76 (-8.42,17.95)	5.05 (-10.63,20.73)	6.40 (-6.39,19.20)	6.61 (-9.55,22.78)	9.33 (-6.20,24.86)	10.89 (-1.60,23.37)	15.39 (-7.96,38.74)	14.70 (4.61,24.79)	21.39 (-6.47,49.24)
-2.86 (-35.56,29.84)	1.61 (-24.34,27.56)	Dulaglutide	6.37 (-17.34,30.09)	6.66 (-17.97,31.29)	8.01 (-15.49,31.52)	8.22 (-17.69,34.13)	10.94 (-14.83,36.70)	12.50 (-10.25,35.25)	17.00 (-13.12,47.12)	16.31 (-7.60,40.22)	23.00 (-10.73,56.73)
-9.24 (-33.52,15.04)	-4.76 (-17.95,8.42)	-6.37 (-30.09,17.34)	Dapagliflozin	0.29 (-11.30,11.87)	1.64 (-6.90,10.18)	1.85 (-11.82,15.51)	4.57 (-8.70,17.83)	6.13 (-0.57,12.82)	10.63 (-10.21,31.46)	9.94 (1.46,18.42)	16.62 (-9.16,42.41)
-9.52 (-34.82,15.78)	-5.05 (-20.73,10.63)	-6.66 (-31.29,17.97)	-0.29 (-11.87,11.30)	Exenatide	1.35 (-9.75,12.46)	1.56 (-13.98,17.10)	4.28 (-11.04,19.60)	5.84 (-3.58,15.26)	10.34 (-11.53,32.21)	9.65 (-2.35,21.65)	16.34 (-10.28,42.96)
-10.87 (-33.60,11.85)	-6.40 (-19.20,6.39)	-8.01 (-31.52,15.49)	-1.64 (-10.18,6.90)	-1.35 (-12.46,9.75)	Liraglutide	0.21 (-13.05,13.46)	2.93 (-9.93,15.78)	4.49 (-1.41,10.38)	8.99 (-11.61,29.58)	8.30 (0.43,16.16)	14.99 (-10.60,40.57)
-11.08 (-37.40,15.23)	-6.61 (-22.78,9.55)	-8.22 (-34.13,17.69)	-1.85 (-15.51,11.82)	-1.56 (-17.10,13.98)	-0.21 (-13.46,13.05)	Ipragliflozin	2.72 (-13.77,19.20)	4.28 (-8.12,16.67)	8.78 (-14.52,32.08)	8.09 (-4.54,20.72)	14.78 (-13.04,42.59)
-13.80 (-39.91,12.31)	-9.33 (-24.86,6.20)	-10.94 (-36.70,14.83)	-4.57 (-17.83,8.70)	-4.28 (-19.60,11.04)	-2.93 (-15.78,9.93)	-2.72 (-19.20,13.77)	Empagliflozin	1.56 (-10.53,13.65)	6.06 (-17.08,29.20)	5.37 (-6.43,17.17)	12.06 (-15.62,39.74)
-15.36 (-38.84,8.12)	-10.89 (-23.37,1.60)	-12.50 (-35.25,10.25)	-6.13 (-12.82,0.57)	-5.84 (-15.26,3.58)	-4.49 (-10.38,1.41)	-4.28 (-16.67,8.12)	-1.56 (-13.65,10.53)	Active control	4.50 (-15.23,24.23)	3.81 (-3.54,11.16)	10.50 (-14.40,35.40)
-19.86 (-50.53,10.81)	-15.39 (-38.74,7.96)	-17.00 (-47.12,13.12)	-10.63 (-31.46,10.21)	-10.34 (-32.21,11.53)	-8.99 (-29.58,11.61)	-8.78 (-32.08,14.52)	-6.06 (-29.20,17.08)	-4.50 (-24.23,15.23)	Luseogliflozin	-0.69 (-21.75,20.37)	6.00 (-25.77,37.77)
-19.17 (-43.22,4.88)	**-14.70 (-24.79,-4.61)**	-16.31 (-40.22,7.60)	**-9.94 (-18.42,-1.46)**	-9.65 (-21.65,2.35)	**-8.30 (-16.16,-0.43)**	-8.09 (-20.72,4.54)	-5.37 (-17.17,6.43)	-3.81 (-11.16,3.54)	0.69 (-20.37,21.75)	Placebo	6.69 (-19.27,32.65)
-25.86 (-60.09,8.37)	-21.39 (-49.24,6.47)	-23.00 (-56.73,10.73)	-16.62 (-42.41,9.16)	-16.34 (-42.96,10.28)	-14.99 (-40.57,10.60)	-14.78 (-42.59,13.04)	-12.06 (-39.74,15.62)	-10.50 (-35.40,14.40)	-6.00 (-37.77,25.77)	-6.69 (-32.65,19.27)	Tofogliflozin

Table 2 Aspartate aminotransferase (AST)

The columns present the row drug class compared to the column drug class. The rows present the row drug class compared to the column drug class. The effect estimates are expressed as mean difference and 95% confidence interval. For example, the mean difference in AST for dulaglutide therapy compared to diet and exercise therapy is -2.10 U/L (95% confidence interval -21.46 U/L to 17.26 U/L). The mean difference in AST for diet and exercise therapy compared to dulaglutide therapy is the inverse (2.10 U/L (95% confidence interval -17.26 U/L to 21.46 U/L)).

Dulaglutide	2.10 (-17.26,21.46)	4.02 (-11.95,19.99)	4.85 (-10.38,20.07)	6.26 (-9.85,22.37)	6.64 (-8.17,21.45)	8.27 (-7.68,24.21)	8.75 (-5.86,23.36)	9.30 (-4.87,23.47)	13.34 (-1.53,28.22)	21.40 (1.21,41.59)
-2.10 (-21.46,17.26)	Diet and exercise	1.92 (-12.87,16.71)	2.74 (-11.57,17.06)	4.16 (-10.93,19.25)	4.54 (-9.26,18.33)	6.17 (-8.72,21.05)	6.65 (-6.05,19.34)	7.20 (-5.99,20.39)	11.24 (-2.36,24.84)	19.30 (-0.22,38.82)
-4.02 (-19.99,11.95)	-1.92 (-16.71,12.87)	Semaglutide	0.82 (-8.43,10.08)	2.24 (-7.38,11.86)	2.62 (-5.26,10.49)	4.25 (-4.84,13.33)	4.73 (-2.85,12.30)	5.28 (-2.09,12.64)	9.32 (3.52,15.12)	17.38 (1.22,33.54)
-4.85 (-20.07,10.38)	-2.74 (-17.06,11.57)	-0.82 (-10.08,8.43)	Exenatide	1.42 (-8.01,10.85)	1.79 (-5.30,8.88)	3.42 (-5.76,12.60)	3.90 (-2.70,10.51)	4.45 (-1.12,10.03)	8.50 (1.29,15.70)	16.55 (1.13,31.98)
-6.26 (-22.37,9.85)	-4.16 (-19.25,10.93)	-2.24 (-11.86,7.38)	-1.42 (-10.85,8.01)	Ipragliflozin	0.38 (-8.17,8.92)	2.00 (-7.95,11.95)	2.48 (-5.67,10.63)	3.04 (-4.64,10.71)	7.08 (-0.60,14.76)	15.14 (-1.17,31.44)
-6.64 (-21.45,8.17)	-4.54 (-18.33,9.26)	-2.62 (-10.49,5.26)	-1.79 (-8.88,5.30)	-0.38 (-8.92,8.17)	Dapagliflozin	1.63 (-6.49,9.74)	2.11 (-3.29,7.50)	2.66 (-1.65,6.97)	6.70 (1.37,12.03)	14.76 (-0.26,29.78)
-8.27 (-24.21,7.68)	-6.17 (-21.05,8.72)	-4.25 (-13.33,4.84)	-3.42 (-12.60,5.76)	-2.00 (-11.95,7.95)	-1.63 (-9.74,6.49)	Empagliflozin	0.48 (-7.28,8.24)	1.03 (-6.28,8.34)	5.08 (-1.92,12.07)	13.13 (-3.00,29.27)
-8.75 (-23.36,5.86)	-6.65 (-19.34,6.05)	-4.73 (-12.30,2.85)	-3.90 (-10.51,2.70)	-2.48 (-10.63,5.67)	-2.11 (-7.50,3.29)	-0.48 (-8.24,7.28)	Liraglutide	0.55 (-3.02,4.12)	4.60 (-0.29,9.48)	12.65 (-2.17,27.48)
-9.30 (-23.47,4.87)	-7.20 (-20.39,5.99)	-5.28 (-12.64,2.09)	-4.45 (-10.03,1.12)	-3.04 (-10.71,4.64)	-2.66 (-6.97,1.65)	-1.03 (-8.34,6.28)	-0.55 (-4.12,3.02)	Active control	4.04 (-0.50,8.58)	12.10 (-2.29,26.49)
-13.34 (-28.22,1.53)	-11.24 (-24.84,2.36)	**-9.32 (-15.12,-3.52)**	**-8.50 (-15.70,-1.29)**	-7.08 (-14.76,0.60)	**-6.70 (-12.03,-1.37)**	-5.08 (-12.07,1.92)	-4.60 (-9.48,0.29)	-4.04 (-8.58,0.50)	Placebo	8.06 (-7.03,23.14)
-21.40 (-41.59,-1.21)	-19.30 (-38.82,0.22)	-17.38 (-33.54,-1.22)	-16.55 (-31.98,-1.13)	-15.14 (-31.44,1.17)	-14.76 (-29.78,0.26)	-13.13 (-29.27,3.00)	-12.65 (-27.48,2.17)	-12.10 (-26.49,2.29)	-8.06 (-23.14,7.03)	Tofogliflozin

Table 3 γ-glutamyl transferase (GGT)

The columns present the row drug class compared to the column drug class. The rows present the row drug class compared to the column drug class. The effect estimates are expressed as mean difference and 95% confidence interval. For example, the mean difference in GGT for dulaglutide therapy compared to semaglutide therapy is -2.14 U/L (95% confidence interval -30.67 U/L to 26.39 U/L). The mean difference in GGT for semaglutide therapy compared to dulaglutide therapy is the inverse (2.14 U/L (95% confidence interval -26.39 U/L to 30.67 U/L)).

Dulaglutide	2.14 (-26.39,30.67)	4.88 (-18.46,28.22)	2.10 (-34.55,38.75)	8.33 (-19.18,35.85)	11.41 (-13.06,35.87)	11.41 (-13.26,36.08)	13.10 (-8.63,34.82)	18.70 (-7.73,45.13)	42.50 (7.76,77.23)
-2.14 (-30.67,26.39)	Semaglutide	2.74 (-17.69,23.18)	-0.04 (-34.87,34.79)	6.19 (-12.81,25.20)	9.27 (-7.28,25.82)	9.27 (-12.68,31.22)	10.96 (-7.54,29.46)	16.56 (5.82,27.30)	40.36 (7.54,73.17)
-4.88 (-28.22,18.46)	-2.74 (-23.18,17.69)	Dapagliflozin	-2.78 (-33.51,27.94)	3.45 (-15.50,22.40)	6.53 (-7.63,20.68)	6.53 (-7.93,20.99)	8.22 (-0.32,16.75)	13.82 (-3.57,31.20)	37.61 (9.20,66.03)
-2.10 (-38.75,34.55)	0.04 (-34.79,34.87)	2.78 (-27.94,33.51)	Empagliflozin	6.23 (-27.77,40.24)	9.31 (-22.28,40.90)	9.31 (-22.44,41.06)	11.00 (-18.52,40.52)	16.60 (-16.53,49.74)	40.40 (0.32,80.47)
-8.33 (-35.85,19.18)	-6.19 (-25.20,12.81)	-3.45 (-22.40,15.50)	-6.23 (-40.24,27.77)	Ipragliflozin	3.07 (-14.17,20.31)	3.08 (-17.49,23.65)	4.76 (-12.12,21.65)	10.37 (-5.31,26.04)	34.16 (2.23,66.10)
-11.41 (-35.87,13.06)	-9.27 (-25.82,7.28)	-6.53 (-20.68,7.63)	-9.31 (-40.90,22.28)	-3.07 (-20.31,14.17)	Liraglutide	0.00 (-16.25,16.26)	1.69 (-9.56,12.94)	7.29 (-5.30,19.88)	31.09 (1.74,60.44)
-11.41 (-36.08,13.26)	-9.27 (-31.22,12.68)	-6.53 (-20.99,7.93)	-9.31 (-41.06,22.44)	-3.08 (-23.65,17.49)	-0.00 (-16.26,16.25)	Exenatide	1.69 (-10.01,13.38)	7.29 (-11.85,26.43)	31.08 (1.56,60.60)
-13.10 (-34.82,8.63)	-10.96 (-29.46,7.54)	-8.22 (-16.75,0.32)	-11.00 (-40.52,18.52)	-4.76 (-21.65,12.12)	-1.69 (-12.94,9.56)	-1.69 (-13.38,10.01)	Active control	5.60 (-9.45,20.66)	29.40 (2.29,56.50)
-18.70 (-45.13,7.73)	**-16.56 (-27.30,-5.82)**	-13.82 (-31.20,3.57)	-16.60 (-49.74,16.53)	-10.37 (-26.04,5.31)	-7.29 (-19.88,5.30)	-7.29 (-26.43,11.85)	-5.60 (-20.66,9.45)	Placebo	23.80 (-7.21,54.80)
-42.50 (-77.23,-7.76)	-40.36 (-73.17,-7.54)	-37.61 (-66.03,-9.20)	-40.40 (-80.47,-0.32)	-34.16 (-66.10,-2.23)	-31.09 (-60.44,-1.74)	-31.08 (-60.60,-1.56)	-29.40 (-56.50,-2.29)	-23.80 (-54.80,7.21)	Tofogliflozin

Table 4 Subcutaneous adipose tissue (SAT)

The columns present the row drug class compared to the column drug class. The rows present the row drug class compared to the column drug class. The effect estimates are expressed as mean difference and 95% confidence interval. For example, the mean difference in SAT for exenatide therapy compared to liraglutide therapy is -0.66 cm^2 (95% confidence interval -27.84 cm^2 to 26.52 cm^2). The mean difference in SAT for liraglutide therapy compared to exenatide therapy is the inverse (0.66 cm^2 (95% confidence interval -26.52 cm^2 to 27.84 cm^2)).

Exenatide	0.66 (-26.52,27.84)	22.97 (-2.03,47.97)	30.67 (5.93,55.40)	30.93 (6.20,55.67)	31.03 (6.30,55.76)
-0.66 (-27.84,26.52)	Liraglutide	22.31 (10.48,34.15)	30.01 (18.75,41.27)	30.27 (19.01,41.54)	30.37 (19.11,41.63)
-22.97 (-47.97,2.03)	-22.31 (-34.15,-10.48)	Ipragliflozin	7.70 (4.06,11.34)	7.96 (4.33,11.60)	8.06 (4.42,11.70)
-30.67 (-55.40,-5.93)	-30.01 (-41.27,-18.75)	-7.70 (-11.34,-4.06)	Dapagliflozin	0.26 (0.17,0.36)	0.36 (0.24,0.48)
**-30.93 (-55.67,-6.20)**	**-30.27 (-41.54,-19.01)**	**-7.96 (-11.60,-4.33)**	**-0.26 (-0.36,-0.17)**	Placebo	0.10 (-0.01,0.21)
-31.03 (-55.76,-6.30)	-30.37 (-41.63,-19.11)	-8.06 (-11.70,-4.42)	-0.36 (-0.48,-0.24)	-0.10 (-0.21,0.01)	Active control

Table 5 Visceral adipose tissue (VAT)

The columns present the row drug class compared to the column drug class. The rows present the row drug class compared to the column drug class. The effect estimates are expressed as mean difference and 95% confidence interval. For example, the mean difference in VAT for exenatide therapy compared to liraglutide therapy is -10.14 cm^2 (95% confidence interval -44.95 cm^2 to 24.66 cm^2). The mean difference in VAT for liraglutide therapy compared to exenatide therapy is the inverse (10.14 cm^2 (95% confidence interval -24.66 cm^2 to 44.95 cm^2)).

Exenatide	10.14 (-24.66,44.95)	15.14 (-23.89,54.17)	28.81 (-9.08,66.71)	33.31 (-0.43,67.05)	35.27 (2.99,67.55)	40.26 (6.21,74.32)
-10.14 (-44.95,24.66)	Liraglutide	5.00 (-19.43,29.43)	18.67 (-4.24,41.59)	23.17 (7.49,38.85)	25.13 (12.12,38.14)	30.12 (14.89,45.36)
-15.14 (-54.17,23.89)	-5.00 (-29.43,19.43)	Ipragliflozin	13.68 (-13.18,40.53)	18.17 (-4.25,40.59)	20.13 (-1.80,42.06)	25.13 (5.76,44.49)
-28.81 (-66.71,9.08)	-18.67 (-41.59,4.24)	-13.68 (-40.53,13.18)	Empagliflozin	4.49 (-16.29,25.28)	6.45 (-13.39,26.29)	11.45 (-7.22,30.12)
-33.31 (-67.05,0.43)	-23.17 (-38.85,-7.49)	-18.17 (-40.59,4.25)	-4.49 (-25.28,16.29)	Dapagliflozin	1.96 (-7.84,11.76)	6.96 (-4.46,18.37)
-35.27 (-67.55,-2.99)	-25.13 (-38.14,-12.12)	-20.13 (-42.06,1.80)	-6.45 (-26.29,13.39)	-1.96 (-11.76,7.84)	Active control	4.99 (-5.85,15.84)
**-40.26 (-74.32,-6.21)**	**-30.12 (-45.36,-14.89)**	**-25.13 (-44.49,-5.76)**	-11.45 (-30.12,7.22)	-6.96 (-18.37,4.46)	-4.99 (-15.84,5.85)	Placebo

Table 6 Liver fat fraction (LFF)

The columns present the row drug class compared to the column drug class. The rows present the row drug class compared to the column drug class. The effect estimates are expressed as mean difference and 95% confidence interval. For example, the mean difference in LFF for diet and exercise therapy compared to liraglutide therapy is -1.36 % (95% confidence interval -7.10 % to 4.37 %). The mean difference in LFF for liraglutide therapy compared to diet and exercise therapy is the inverse (1.36 % (95% confidence interval -4.37 % to 7.10 %)).

Diet and exercise	1.36 (-4.37,7.10)	4.18 (-1.68,10.04)	5.57 (-0.67,11.82)	6.53 (0.33,12.72)	7.60 (0.48,14.73)
-1.36 (-7.10,4.37)	Liraglutide	2.82 (1.60,4.04)	4.21 (1.73,6.69)	5.16 (2.81,7.51)	6.24 (2.00,10.48)
-4.18 (-10.04,1.68)	-2.82 (-4.04,-1.60)	Active control	1.39 (-0.78,3.56)	2.34 (0.33,4.36)	3.42 (-0.64,7.48)
-5.57 (-11.82,0.67)	-4.21 (-6.69,-1.73)	-1.39 (-3.56,0.78)	Dapagliflozin	0.95 (-0.62,2.52)	2.03 (-2.57,6.63)
**-6.53 (-12.72,-0.33)**	**-5.16 (-7.51,-2.81)**	**-2.34 (-4.36,-0.33)**	-0.95 (-2.52,0.62)	Placebo	1.08 (-3.45,5.61)
-7.60 (-14.73,-0.48)	-6.24 (-10.48,-2.00)	-3.42 (-7.48,0.64)	-2.03 (-6.63,2.57)	-1.08 (-5.61,3.45)	Tofogliflozin

Table 7 Controlled attenuation parameter (CAP)

The columns present the row drug class compared to the column drug class. The rows present the row drug class compared to the column drug class. The effect estimates are expressed as mean difference and 95% confidence interval. For example, the mean difference in CAP for dapagliflozin therapy compared to ipragliflozin therapy is -19.16 db/m (95% confidence interval -60.19 db/m to 21.87 db/m). The mean difference in CAP for ipragliflozin therapy compared to dapagliflozin therapy is the inverse (19.16 db/m (95% confidence interval -21.87 db/m to 60.19 db/m)).

Dapagliflozin	19.16 (-21.87,60.19)	23.29 (-13.86,60.44)	22.16 (-50.14,94.46)	29.60 (5.28,53.91)	29.82 (-4.45,64.09)	38.86 (4.33,73.39)
-19.16 (-60.19,21.87)	Ipragliflozin	4.13 (-21.93,30.19)	3.00 (-64.27,70.27)	10.44 (-22.61,43.49)	10.66 (-17.79,39.11)	19.70 (-2.46,41.86)
-23.29 (-60.44,13.86)	-4.13 (-30.19,21.93)	Semaglutide	-1.13 (-66.11,63.85)	6.31 (-21.79,34.40)	6.53 (-15.98,29.04)	15.57 (1.85,29.29)
-22.16 (-94.46,50.14)	-3.00 (-70.27,64.27)	1.13 (-63.85,66.11)	Liraglutide	7.44 (-60.65,75.52)	7.66 (-58.32,73.64)	16.70 (-46.82,80.22)
-29.60 (-53.91,-5.28)	-10.44 (-43.49,22.61)	-6.31 (-34.40,21.79)	-7.44 (-75.52,60.65)	Active control	0.22 (-23.92,24.37)	9.26 (-15.26,33.78)
-29.82 (-64.09,4.45)	-10.66 (-39.11,17.79)	-6.53 (-29.04,15.98)	-7.66 (-73.64,58.32)	-0.22 (-24.37,23.92)	Empagliflozin	9.04 (-8.81,26.88)
**-38.86 (-73.39,-4.33)**	-19.70 (-41.86,2.46)	**-15.57 (-29.29,-1.85)**	-16.70 (-80.22,46.82)	-9.26 (-33.78,15.26)	-9.04 (-26.88,8.81)	Placebo

Table 8 Liver stiffness measurement (LSM)

The columns present the row drug class compared to the column drug class. The rows present the row drug class compared to the column drug class. The effect estimates are expressed as mean difference and 95% confidence interval. For example, the mean difference in LSM for semaglutide therapy compared to dapagliflozin therapy is -1.42 kPa (95% confidence interval -4.26 kPa to 1.42 kPa). The mean difference in LSM for dapagliflozin therapy compared to semaglutide therapy is the inverse (1.42 kPa (95% confidence interval -1.42 kPa to 4.26 kPa)).

Semaglutide	1.42 (-1.42,4.26)	2.04 (-0.01,4.08)	2.59 (1.90,3.28)	3.08 (2.77,3.39)	3.35 (2.27,4.42)	3.55 (2.16,4.94)
-1.42 (-4.26,1.42)	Dapagliflozin	0.62 (-2.53,3.77)	1.17 (-1.64,3.99)	1.67 (-1.16,4.49)	1.93 (-0.70,4.56)	2.13 (-0.64,4.90)
-2.04 (-4.08,0.01)	-0.62 (-3.77,2.53)	Dulaglutide	0.55 (-1.45,2.56)	1.05 (-0.97,3.07)	1.31 (-0.43,3.04)	1.51 (-0.43,3.45)
-2.59 (-3.28,-1.90)	-1.17 (-3.99,1.64)	-0.55 (-2.56,1.45)	Empagliflozin	0.49 (-0.12,1.11)	0.76 (-0.25,1.76)	0.96 (-0.38,2.29)
**-3.08 (-3.39,-2.77)**	-1.67 (-4.49,1.16)	-1.05 (-3.07,0.97)	-0.49 (-1.11,0.12)	Placebo	0.26 (-0.77,1.30)	0.46 (-0.89,1.82)
-3.35 (-4.42,-2.27)	-1.93 (-4.56,0.70)	-1.31 (-3.04,0.43)	-0.76 (-1.76,0.25)	-0.26 (-1.30,0.77)	Active control	0.20 (-0.68,1.08)
-3.55 (-4.94,-2.16)	-2.13 (-4.90,0.64)	-1.51 (-3.45,0.43)	-0.96 (-2.29,0.38)	-0.46 (-1.82,0.89)	-0.20 (-1.08,0.68)	Tofogliflozin

Table 9 Body weight (BW)

The columns present the row drug class compared to the column drug class. The rows present the row drug class compared to the column drug class. The effect estimates are expressed as mean difference and 95% confidence interval. For example, the mean difference in BW for semaglutide therapy compared to exenatide therapy is -3.74 kg (95% confidence interval -8.71 kg to 1.23 kg). The mean difference in BW for exenatide therapy compared to semaglutide therapy is the inverse (3.74 kg (95% confidence interval -1.23 kg to 8.71 kg)).

Semaglutide	3.74 (-1.23,8.71)	4.15 (-1.82,10.12)	4.40 (0.20,8.60)	4.66 (0.58,8.75)	5.11 (-0.48,10.69)	5.55 (-0.21,11.30)	6.00 (-1.11,13.12)	8.14 (4.84,11.45)	8.30 (4.27,12.33)	9.74 (2.62,16.86)
-3.74 (-8.71,1.23)	Exenatide	0.41 (-5.17,5.98)	0.66 (-2.96,4.28)	0.92 (-2.77,4.62)	1.37 (-4.12,6.85)	1.81 (-3.78,7.40)	2.26 (-4.28,8.80)	4.40 (0.69,8.12)	4.56 (1.65,7.47)	6.00 (-0.55,12.55)
-4.15 (-10.12,1.82)	-0.41 (-5.98,5.17)	Diet and exercise	0.25 (-3.99,4.49)	0.52 (-4.62,5.65)	0.96 (-5.53,7.45)	1.40 (-5.20,8.00)	1.85 (-5.70,9.40)	4.00 (-0.97,8.97)	4.15 (-0.60,8.91)	5.59 (-1.96,13.14)
-4.40 (-8.60,-0.20)	-0.66 (-4.28,2.96)	-0.25 (-4.49,3.99)	Liraglutide	0.27 (-2.63,3.16)	0.71 (-4.20,5.62)	1.15 (-3.91,6.21)	1.60 (-4.64,7.85)	3.75 (1.15,6.34)	3.90 (1.75,6.05)	5.34 (-0.91,11.59)
-4.66 (-8.75,-0.58)	-0.92 (-4.62,2.77)	-0.52 (-5.65,4.62)	-0.27 (-3.16,2.63)	Dapagliflozin	0.44 (-4.44,5.32)	0.88 (-4.15,5.92)	1.34 (-4.95,7.63)	3.48 (1.08,5.88)	3.64 (1.35,5.92)	5.08 (-1.22,11.37)
-5.11 (-10.69,0.48)	-1.37 (-6.85,4.12)	-0.96 (-7.45,5.53)	-0.71 (-5.62,4.20)	-0.44 (-5.32,4.44)	Ipragliflozin	0.44 (-5.94,6.82)	0.89 (-6.59,8.38)	3.04 (-1.47,7.54)	3.19 (-1.45,7.84)	4.63 (-2.85,12.12)
-5.55 (-11.30,0.21)	-1.81 (-7.40,3.78)	-1.40 (-8.00,5.20)	-1.15 (-6.21,3.91)	-0.88 (-5.92,4.15)	-0.44 (-6.82,5.94)	Empagliflozin	0.45 (-7.11,8.01)	2.60 (-2.12,7.31)	2.75 (-2.02,7.53)	4.19 (-3.37,11.76)
-6.00 (-13.12,1.11)	-2.26 (-8.80,4.28)	-1.85 (-9.40,5.70)	-1.60 (-7.85,4.64)	-1.34 (-7.63,4.95)	-0.89 (-8.38,6.59)	-0.45 (-8.01,7.11)	Dulaglutide	2.14 (-4.16,8.45)	2.30 (-3.56,8.16)	3.74 (-4.55,12.03)
**-8.14 (-11.45,-4.84)**	**-4.40 (-8.12,-0.69)**	-4.00 (-8.97,0.97)	**-3.75 (-6.34,-1.15)**	**-3.48 (-5.88,-1.08)**	-3.04 (-7.54,1.47)	-2.60 (-7.31,2.12)	-2.14 (-8.45,4.16)	Placebo	0.16 (-2.16,2.47)	1.60 (-4.71,7.90)
-8.30 (-12.33,-4.27)	-4.56 (-7.47,-1.65)	-4.15 (-8.91,0.60)	-3.90 (-6.05,-1.75)	-3.64 (-5.92,-1.35)	-3.19 (-7.84,1.45)	-2.75 (-7.53,2.02)	-2.30 (-8.16,3.56)	-0.16 (-2.47,2.16)	Active control	1.44 (-4.43,7.31)
-9.74 (-16.86,-2.62)	-6.00 (-12.55,0.55)	-5.59 (-13.14,1.96)	-5.34 (-11.59,0.91)	-5.08 (-11.37,1.22)	-4.63 (-12.12,2.85)	-4.19 (-11.76,3.37)	-3.74 (-12.03,4.55)	-1.60 (-7.90,4.71)	-1.44 (-7.31,4.43)	Tofogliflozin

Table 10 Body mass index (BMI)

The columns present the row drug class compared to the column drug class. The rows present the row drug class compared to the column drug class. The effect estimates are expressed as mean difference and 95% confidence interval. For example, the mean difference in BMI for exenatide therapy compared to diet and exercise therapy is -0.17 kg/m² (95% confidence interval -1.94 kg/m² to 1.60 kg/m²). The mean difference in BMI for diet and exercise therapy compared to exenatide therapy is the inverse (0.17 kg/m² (95% confidence interval -1.60 kg/m² to 1.94 kg/m²)).

Exenatide	0.17 (-1.60,1.94)	0.37 (-0.76,1.50)	0.73 (-0.59,2.05)	0.79 (-1.14,2.72)	0.79 (-0.88,2.47)	0.85 (-1.26,2.97)	1.03 (-0.99,3.04)	1.67 (0.76,2.59)	1.86 (0.59,3.12)
-0.17 (-1.94,1.60)	Diet and exercise	0.20 (-1.15,1.56)	0.56 (-1.18,2.30)	0.62 (-1.58,2.82)	0.62 (-1.39,2.63)	0.68 (-1.75,3.11)	0.86 (-1.49,3.20)	1.50 (-0.01,3.02)	1.69 (0.06,3.31)
-0.37 (-1.50,0.76)	-0.20 (-1.56,1.15)	Liraglutide	0.36 (-0.73,1.45)	0.42 (-1.32,2.16)	0.42 (-1.06,1.90)	0.48 (-1.53,2.50)	0.66 (-1.26,2.57)	1.30 (0.63,1.97)	1.49 (0.59,2.39)
-0.73 (-2.05,0.59)	-0.56 (-2.30,1.18)	-0.36 (-1.45,0.73)	Dapagliflozin	0.06 (-1.76,1.88)	0.06 (-1.53,1.65)	0.12 (-2.00,2.25)	0.29 (-1.74,2.33)	0.94 (-0.01,1.89)	1.13 (0.11,2.14)
-0.79 (-2.72,1.14)	-0.62 (-2.82,1.58)	-0.42 (-2.16,1.32)	-0.06 (-1.88,1.76)	Ipragliflozin	0.00 (-2.05,2.05)	0.06 (-2.49,2.62)	0.24 (-2.24,2.71)	0.88 (-0.82,2.59)	1.07 (-0.50,2.64)
-0.79 (-2.47,0.88)	-0.62 (-2.63,1.39)	-0.42 (-1.90,1.06)	-0.06 (-1.65,1.53)	-0.00 (-2.05,2.05)	Empagliflozin	0.06 (-2.30,2.43)	0.24 (-2.04,2.51)	0.88 (-0.52,2.29)	1.07 (-0.31,2.45)
-0.85 (-2.97,1.26)	-0.68 (-3.11,1.75)	-0.48 (-2.50,1.53)	-0.12 (-2.25,2.00)	-0.06 (-2.62,2.49)	-0.06 (-2.43,2.30)	Dulaglutide	0.17 (-2.44,2.79)	0.82 (-1.08,2.72)	1.00 (-1.09,3.10)
-1.03 (-3.04,0.99)	-0.86 (-3.20,1.49)	-0.66 (-2.57,1.26)	-0.29 (-2.33,1.74)	-0.24 (-2.71,2.24)	-0.24 (-2.51,2.04)	-0.17 (-2.79,2.44)	Luseogliflozin	0.65 (-1.15,2.44)	0.83 (-1.16,2.83)
-1.67 (-2.59,-0.76)	-1.50 (-3.02,0.01)	-1.30 (-1.97,-0.63)	-0.94 (-1.89,0.01)	-0.88 (-2.59,0.82)	-0.88 (-2.29,0.52)	-0.82 (-2.72,1.08)	-0.65 (-2.44,1.15)	Active control	0.18 (-0.69,1.06)
**-1.86 (-3.12,-0.59)**	**-1.69 (-3.31,-0.06)**	**-1.49 (-2.39,-0.59)**	**-1.13 (-2.14,-0.11)**	-1.07 (-2.64,0.50)	-1.07 (-2.45,0.31)	-1.00 (-3.10,1.09)	-0.83 (-2.83,1.16)	-0.18 (-1.06,0.69)	Placebo

Table 11 Waist circumference (WC)

The columns present the row drug class compared to the column drug class. The rows present the row drug class compared to the column drug class. The effect estimates are expressed as mean difference and 95% confidence interval. For example, the mean difference in WC for exenatide therapy compared to liraglutide therapy is -1.59 cm (95% confidence interval -5.40 cm to 2.23 cm). The mean difference in WC for liraglutide therapy compared to exenatide therapy is the inverse (1.59 cm (95% confidence interval -2.23 cm to 5.40 cm)).

Exenatide	1.59 (-2.23,5.40)	2.42 (-4.15,8.99)	2.64 (-2.97,8.26)	3.34 (-1.06,7.74)	4.82 (-2.67,12.31)	5.22 (2.15,8.28)	6.12 (1.97,10.26)
-1.59 (-5.40,2.23)	Liraglutide	0.83 (-5.01,6.66)	1.05 (-3.07,5.18)	1.75 (-1.73,5.23)	3.23 (-3.62,10.08)	3.63 (1.35,5.90)	4.53 (1.69,7.37)
-2.42 (-8.99,4.15)	-0.83 (-6.66,5.01)	Ipragliflozin	0.23 (-6.92,7.37)	0.92 (-4.91,6.75)	2.40 (-5.65,10.45)	2.80 (-3.02,8.61)	3.70 (-1.40,8.80)
-2.64 (-8.26,2.97)	-1.05 (-5.18,3.07)	-0.23 (-7.37,6.92)	Diet and exercise	0.70 (-4.70,6.09)	2.17 (-5.82,10.17)	2.57 (-2.14,7.28)	3.47 (-1.53,8.48)
-3.34 (-7.74,1.06)	-1.75 (-5.23,1.73)	-0.92 (-6.75,4.91)	-0.70 (-6.09,4.70)	Dapagliflozin	1.48 (-5.37,8.33)	1.88 (-1.29,5.04)	2.78 (-0.06,5.61)
-4.82 (-12.31,2.67)	-3.23 (-10.08,3.62)	-2.40 (-10.45,5.65)	-2.17 (-10.17,5.82)	-1.48 (-8.33,5.37)	Empagliflozin	0.40 (-6.44,7.23)	1.30 (-4.94,7.54)
-5.22 (-8.28,-2.15)	-3.63 (-5.90,-1.35)	-2.80 (-8.61,3.02)	-2.57 (-7.28,2.14)	-1.88 (-5.04,1.29)	-0.40 (-7.23,6.44)	Active control	0.90 (-1.90,3.70)
**-6.12 (-10.26,-1.97)**	**-4.53 (-7.37,-1.69)**	-3.70 (-8.80,1.40)	-3.47 (-8.48,1.53)	-2.78 (-5.61,0.06)	-1.30 (-7.54,4.94)	-0.90 (-3.70,1.90)	Placebo

Table 12 Systobic blood pressure (SBP)

The columns present the row drug class compared to the column drug class. The rows present the row drug class compared to the column drug class. The effect estimates are expressed as mean difference and 95% confidence interval. For example, the mean difference in SBP for ipragliflozin therapy compared to dapagliflozin therapy is -0.29 mmHg (95% confidence interval -6.04 mmHg to 5.46 mmHg). The mean difference in SBP for dapagliflozin therapy compared to ipragliflozin therapy is the inverse (0.29 mmHg (95% confidence interval -5.46 mmHg to 6.04 mmHg)).

Ipragliflozin	0.29 (-5.46,6.04)	0.63 (-3.19,4.46)	0.79 (-4.00,5.59)	1.66 (-1.82,5.15)	2.99 (0.05,5.94)	3.90 (1.01,6.79)	8.99 (-0.71,18.70)
-0.29 (-6.04,5.46)	Dapagliflozin	0.34 (-5.43,6.12)	0.51 (-5.71,6.72)	1.37 (-5.14,7.89)	2.71 (-2.24,7.65)	3.61 (-2.61,9.84)	8.71 (-1.78,19.19)
-0.63 (-4.46,3.19)	-0.34 (-6.12,5.43)	Liraglutide	0.16 (-4.66,4.98)	1.03 (-3.58,5.64)	2.36 (-0.63,5.35)	3.27 (-0.91,7.45)	8.36 (-1.36,18.09)
-0.79 (-5.59,4.00)	-0.51 (-6.72,5.71)	-0.16 (-4.98,4.66)	Exenatide	0.87 (-4.82,6.56)	2.20 (-1.58,5.98)	3.11 (-2.25,8.46)	8.20 (-1.79,18.19)
-1.66 (-5.15,1.82)	-1.37 (-7.89,5.14)	-1.03 (-5.64,3.58)	-0.87 (-6.56,4.82)	Semaglutide	1.33 (-2.92,5.58)	2.24 (0.27,4.20)	7.33 (-2.85,17.51)
-2.99 (-5.94,-0.05)	-2.71 (-7.65,2.24)	-2.36 (-5.35,0.63)	-2.20 (-5.98,1.58)	-1.33 (-5.58,2.92)	Active control	0.91 (-2.88,4.69)	6.00 (-3.25,15.25)
**-3.90 (-6.79,-1.01)**	-3.61 (-9.84,2.61)	-3.27 (-7.45,0.91)	-3.11 (-8.46,2.25)	**-2.24 (-4.20,-0.27)**	-0.91 (-4.69,2.88)	Placebo	5.09 (-4.90,15.09)
-8.99 (-18.70,0.71)	-8.71 (-19.19,1.78)	-8.36 (-18.09,1.36)	-8.20 (-18.19,1.79)	-7.33 (-17.51,2.85)	-6.00 (-15.25,3.25)	-5.09 (-15.09,4.90)	Empagliflozin

Table 13 Diastolic blood pressure (DBP)

The columns present the row drug class compared to the column drug class. The rows present the row drug class compared to the column drug class. The effect estimates are expressed as mean difference and 95% confidence interval. For example, the mean difference in DBP for dapagliflozin therapy compared to exenatide therapy is -0.82 mm Hg (95% confidence interval -4.71 mm Hg to 3.07 mm Hg). The mean difference in DBP for exenatide therapy compared to dapagliflozin therapy is the inverse (0.82 mm Hg (95% confidence interval -3.07 mm Hg to 4.71 mm Hg)).

Dapagliflozin	0.82 (-3.07,4.71)	1.21 (-2.73,5.15)	1.52 (-1.52,4.56)	1.59 (-2.23,5.41)	1.83 (-1.91,5.57)	1.92 (-1.46,5.31)	3.52 (-3.18,10.22)
-0.82 (-4.71,3.07)	Exenatide	0.38 (-3.12,3.88)	0.70 (-1.73,3.13)	0.77 (-2.60,4.14)	1.01 (-2.26,4.28)	1.10 (-1.75,3.96)	2.70 (-3.75,9.14)
-1.21 (-5.15,2.73)	-0.38 (-3.88,3.12)	Semaglutide	0.31 (-2.20,2.82)	0.38 (-0.64,1.41)	0.63 (-2.53,3.78)	0.72 (-1.37,2.81)	2.31 (-4.16,8.79)
-1.52 (-4.56,1.52)	-0.70 (-3.13,1.73)	-0.31 (-2.82,2.20)	Active control	0.07 (-2.25,2.39)	0.31 (-1.87,2.50)	0.40 (-1.08,1.89)	2.00 (-3.97,7.97)
-1.59 (-5.41,2.23)	-0.77 (-4.14,2.60)	-0.38 (-1.41,0.64)	-0.07 (-2.39,2.25)	Placebo	0.24 (-2.76,3.24)	0.33 (-1.50,2.17)	1.93 (-4.47,8.33)
-1.83 (-5.57,1.91)	-1.01 (-4.28,2.26)	-0.63 (-3.78,2.53)	-0.31 (-2.50,1.87)	-0.24 (-3.24,2.76)	Liraglutide	0.09 (-2.47,2.65)	1.69 (-4.67,8.05)
-1.92 (-5.31,1.46)	-1.10 (-3.96,1.75)	-0.72 (-2.81,1.37)	-0.40 (-1.89,1.08)	-0.33 (-2.17,1.50)	-0.09 (-2.65,2.47)	Ipragliflozin	1.60 (-4.56,7.75)
-3.52 (-10.22,3.18)	-2.70 (-9.14,3.75)	-2.31 (-8.79,4.16)	-2.00 (-7.97,3.97)	-1.93 (-8.33,4.47)	-1.69 (-8.05,4.67)	-1.60 (-7.75,4.56)	Empagliflozin

Table 14 Blood lipids [total cholesterol(TC)

The columns present the row drug class compared to the column drug class. The rows present the row drug class compared to the column drug class. The effect estimates are expressed as mean difference and 95% confidence interval. For example, the mean difference in TC for exenatide therapy compared to liraglutide therapy is -0.09 mmol/L (95% confidence interval -0.45 mmol/L to 0.27 mmol/L). The mean difference in TC for liraglutide therapy compared to exenatide therapy is the inverse (0.09 mmol/L (95% confidence interval -0.27 mmol/L to 0.45 mmol/L)).

Exenatide	0.09 (-0.27,0.45)	0.10 (-0.49,0.69)	0.16 (-0.12,0.44)	0.32 (-0.48,1.12)	0.33 (-0.07,0.74)	0.38 (-0.03,0.80)	0.46 (-0.02,0.94)	0.63 (0.16,1.09)
-0.09 (-0.45,0.27)	Liraglutide	0.01 (-0.54,0.56)	0.07 (-0.16,0.30)	0.23 (-0.55,1.01)	0.24 (-0.06,0.55)	0.29 (-0.07,0.65)	0.37 (-0.03,0.77)	0.54 (0.09,0.98)
-0.10 (-0.69,0.49)	-0.01 (-0.56,0.54)	Empagliflozin	0.06 (-0.46,0.58)	0.22 (-0.69,1.13)	0.23 (-0.30,0.76)	0.28 (-0.30,0.86)	0.36 (-0.23,0.94)	0.53 (-0.12,1.17)
-0.16 (-0.44,0.12)	-0.07 (-0.30,0.16)	-0.06 (-0.58,0.46)	Active control	0.16 (-0.59,0.91)	0.17 (-0.12,0.47)	0.22 (-0.09,0.53)	0.30 (-0.09,0.69)	0.47 (0.09,0.85)
-0.32 (-1.12,0.48)	-0.23 (-1.01,0.55)	-0.22 (-1.13,0.69)	-0.16 (-0.91,0.59)	Tofogliflozin	0.01 (-0.79,0.81)	0.06 (-0.75,0.87)	0.14 (-0.70,0.98)	0.31 (-0.53,1.14)
-0.33 (-0.74,0.07)	-0.24 (-0.55,0.06)	-0.23 (-0.76,0.30)	-0.17 (-0.47,0.12)	-0.01 (-0.81,0.79)	Placebo	0.05 (-0.28,0.38)	0.13 (-0.13,0.39)	0.30 (-0.19,0.78)
-0.38 (-0.80,0.03)	-0.29 (-0.65,0.07)	-0.28 (-0.86,0.30)	-0.22 (-0.53,0.09)	-0.06 (-0.87,0.75)	-0.05 (-0.38,0.28)	Ipragliflozin	0.08 (-0.34,0.50)	0.24 (-0.25,0.74)
-0.46 (-0.94,0.02)	-0.37 (-0.77,0.03)	-0.36 (-0.94,0.23)	-0.30 (-0.69,0.09)	-0.14 (-0.98,0.70)	-0.13 (-0.39,0.13)	-0.08 (-0.50,0.34)	Semaglutide	0.17 (-0.39,0.72)
-0.63 (-1.09,-0.16)	-0.54 (-0.98,-0.09)	-0.53 (-1.17,0.12)	-0.47 (-0.85,-0.09)	-0.31 (-1.14,0.53)	-0.30 (-0.78,0.19)	-0.24 (-0.74,0.25)	-0.17 (-0.72,0.39)	Dapagliflozin

Table 15 Triglycerides (TG)

The columns present the row drug class compared to the column drug class. The rows present the row drug class compared to the column drug class. The effect estimates are expressed as mean difference and 95% confidence interval. For example, the mean difference in TG for ipragliflozin therapy compared to semaglutide therapy is -0.01 mmol/L (95% confidence interval -0.27 mmol/L to 0.26 mmol/L). The mean difference in TG for semaglutide therapy compared to ipragliflozin therapy is the inverse (0.01 mmol/L (95% confidence interval -0.26 mmol/L to 0.27 mmol/L)).

Ipragliflozin	0.01 (-0.26,0.27)	0.04 (-0.26,0.33)	0.04 (-0.22,0.31)	0.05 (-0.22,0.32)	-0.10 (-2.51,2.32)	0.17 (-0.37,0.70)	0.20 (-0.02,0.43)	0.26 (0.04,0.48)	0.77 (0.19,1.34)
-0.01 (-0.27,0.26)	Semaglutide	0.03 (-0.28,0.33)	0.03 (-0.23,0.30)	0.04 (-0.23,0.31)	-0.11 (-2.52,2.31)	0.16 (-0.37,0.69)	0.19 (-0.05,0.44)	0.25 (0.10,0.40)	0.76 (0.17,1.34)
-0.04 (-0.33,0.26)	-0.03 (-0.33,0.28)	Exenatide	0.01 (-0.24,0.26)	0.01 (-0.24,0.27)	-0.13 (-2.55,2.28)	0.13 (-0.40,0.66)	0.17 (-0.02,0.35)	0.22 (-0.05,0.49)	0.73 (0.17,1.29)
-0.04 (-0.31,0.22)	-0.03 (-0.30,0.23)	-0.01 (-0.26,0.24)	Dapagliflozin	0.01 (-0.23,0.24)	-0.14 (-2.55,2.27)	0.12 (-0.39,0.64)	0.16 (-0.01,0.33)	0.22 (-0.01,0.44)	0.72 (0.17,1.28)
-0.05 (-0.32,0.22)	-0.04 (-0.31,0.23)	-0.01 (-0.27,0.24)	-0.01 (-0.24,0.23)	Liraglutide	-0.15 (-2.56,2.27)	0.12 (-0.40,0.63)	0.15 (-0.02,0.33)	0.21 (-0.02,0.44)	0.72 (0.16,1.27)
0.10 (-2.32,2.51)	0.11 (-2.31,2.52)	0.13 (-2.28,2.55)	0.14 (-2.27,2.55)	0.15 (-2.27,2.56)	Dulaglutide	0.26 (-2.19,2.72)	0.30 (-2.11,2.71)	0.36 (-2.06,2.77)	0.86 (-1.60,3.33)
-0.17 (-0.70,0.37)	-0.16 (-0.69,0.37)	-0.13 (-0.66,0.40)	-0.12 (-0.64,0.39)	-0.12 (-0.63,0.40)	-0.26 (-2.72,2.19)	Empagliflozin	0.04 (-0.46,0.53)	0.09 (-0.41,0.60)	0.60 (-0.12,1.33)
-0.20 (-0.43,0.02)	-0.19 (-0.44,0.05)	-0.17 (-0.35,0.02)	-0.16 (-0.33,0.01)	-0.15 (-0.33,0.02)	-0.30 (-2.71,2.11)	-0.04 (-0.53,0.46)	Active control	0.06 (-0.14,0.25)	0.56 (0.03,1.09)
**-0.26 (-0.48,-0.04)**	**-0.25 (-0.40,-0.10)**	-0.22 (-0.49,0.05)	-0.22 (-0.44,0.01)	-0.21 (-0.44,0.02)	-0.36 (-2.77,2.06)	-0.09 (-0.60,0.41)	-0.06 (-0.25,0.14)	Placebo	0.51 (-0.06,1.07)
-0.77 (-1.34,-0.19)	-0.76 (-1.34,-0.17)	-0.73 (-1.29,-0.17)	-0.72 (-1.28,-0.17)	-0.72 (-1.27,-0.16)	-0.86 (-3.33,1.60)	-0.60 (-1.33,0.12)	-0.56 (-1.09,-0.03)	-0.51 (-1.07,0.06)	Tofogliflozin

Table 16 High density lipoprotein-cholesterol (HDL-C)

The columns present the row drug class compared to the column drug class. The rows present the row drug class compared to the column drug class. The effect estimates are expressed as mean difference and 95% confidence interval. For example, the mean difference in HDL-C for dapagliflozin therapy compared to liraglutide therapy is 0.05 mmol/L (95% confidence interval -0.02 mmol/L to 0.13 mmol/L). The mean difference in HDL-C for liraglutide therapy compared to dapagliflozin therapy is the inverse (-0.05 mmol/L (95% confidence interval -0.13 mmol/L to 0.02 mmol/L)).

Dapagliflozin	-0.05 (-0.13,0.02)	-0.06 (-0.11,-0.01)	-0.06 (-0.15,0.03)	-0.06 (-0.15,0.03)	-0.09 (-0.17,-0.01)	-0.16 (-0.72,0.40)	-0.10 (-0.22,0.01)	-0.14 (-0.21,-0.06)
0.05 (-0.02,0.13)	Liraglutide	-0.00 (-0.06,0.05)	-0.01 (-0.09,0.07)	-0.01 (-0.10,0.08)	-0.03 (-0.11,0.04)	-0.10 (-0.67,0.46)	-0.05 (-0.16,0.06)	-0.08 (-0.15,-0.02)
0.06 (0.01,0.11)	0.00 (-0.05,0.06)	Active control	-0.00 (-0.08,0.07)	-0.01 (-0.08,0.07)	-0.03 (-0.10,0.04)	-0.10 (-0.66,0.46)	-0.04 (-0.15,0.06)	-0.08 (-0.14,-0.02)
0.06 (-0.03,0.15)	0.01 (-0.07,0.09)	0.00 (-0.07,0.08)	Ipragliflozin	-0.00 (-0.11,0.10)	-0.03 (-0.10,0.04)	-0.10 (-0.66,0.47)	-0.04 (-0.16,0.07)	-0.08 (-0.14,-0.01)
0.06 (-0.03,0.15)	0.01 (-0.08,0.10)	0.01 (-0.07,0.08)	0.00 (-0.10,0.11)	Exenatide	-0.02 (-0.13,0.08)	-0.09 (-0.66,0.47)	-0.04 (-0.16,0.09)	-0.07 (-0.17,0.02)
0.09 (0.01,0.17)	0.03 (-0.04,0.11)	0.03 (-0.04,0.10)	0.03 (-0.04,0.10)	0.02 (-0.08,0.13)	Semaglutide	-0.07 (-0.63,0.49)	-0.01 (-0.13,0.10)	-0.05 (-0.09,-0.01)
0.16 (-0.40,0.72)	0.10 (-0.46,0.67)	0.10 (-0.46,0.66)	0.10 (-0.47,0.66)	0.09 (-0.47,0.66)	0.07 (-0.49,0.63)	Dulaglutide	0.06 (-0.51,0.62)	0.02 (-0.54,0.58)
0.10 (-0.01,0.22)	0.05 (-0.06,0.16)	0.04 (-0.06,0.15)	0.04 (-0.07,0.16)	0.04 (-0.09,0.16)	0.01 (-0.10,0.13)	-0.06 (-0.62,0.51)	Empagliflozin	-0.03 (-0.14,0.07)
**0.14 (0.06,0.21)**	**0.08 (0.02,0.15)**	**0.08 (0.02,0.14)**	**0.08 (0.01,0.14)**	0.07 (-0.02,0.17)	**0.05 (0.01,0.09)**	-0.02 (-0.58,0.54)	0.03 (-0.07,0.14)	Placebo

Table 17 Low density lipoprotein-cholesterol (LDL-C)

The columns present the row drug class compared to the column drug class. The rows present the row drug class compared to the column drug class. The effect estimates are expressed as mean difference and 95% confidence interval. For example, the mean difference in LDL-C for exenatide therapy compared to active control therapy is -0.18 mmol/L (95% confidence interval -0.54 mmol/L to 0.18 mmol/L). The mean difference in LDL-C for active control therapy compared to exenatide therapy is the inverse (0.18 mmol/L (95% confidence interval -0.18 mmol/L to 0.54 mmol/L)).

Exenatide	0.18 (-0.18,0.54)	0.18 (-0.38,0.74)	0.22 (-0.20,0.64)	0.25 (-0.18,0.67)	0.38 (-1.84,2.60)	0.28 (-0.18,0.73)	0.34 (-0.18,0.86)	0.44 (-0.12,1.00)
-0.18 (-0.54,0.18)	Active control	0.00 (-0.42,0.43)	0.04 (-0.17,0.26)	0.07 (-0.16,0.30)	0.20 (-1.99,2.39)	0.10 (-0.19,0.38)	0.16 (-0.21,0.54)	0.26 (-0.17,0.69)
-0.18 (-0.74,0.38)	-0.00 (-0.43,0.42)	Empagliflozin	0.04 (-0.43,0.51)	0.06 (-0.42,0.54)	0.20 (-2.04,2.43)	0.09 (-0.37,0.55)	0.16 (-0.40,0.71)	0.25 (-0.31,0.82)
-0.22 (-0.64,0.20)	-0.04 (-0.26,0.17)	-0.04 (-0.51,0.43)	Liraglutide	0.03 (-0.28,0.33)	0.16 (-2.05,2.36)	0.05 (-0.25,0.35)	0.12 (-0.30,0.53)	0.22 (-0.22,0.66)
-0.25 (-0.67,0.18)	-0.07 (-0.30,0.16)	-0.06 (-0.54,0.42)	-0.03 (-0.33,0.28)	Dapagliflozin	0.13 (-2.07,2.34)	0.03 (-0.31,0.37)	0.09 (-0.34,0.52)	0.19 (-0.28,0.66)
-0.38 (-2.60,1.84)	-0.20 (-2.39,1.99)	-0.20 (-2.43,2.04)	-0.16 (-2.36,2.05)	-0.13 (-2.34,2.07)	Dulaglutide	-0.10 (-2.31,2.11)	-0.04 (-2.26,2.18)	0.06 (-2.17,2.29)
-0.28 (-0.73,0.18)	-0.10 (-0.38,0.19)	-0.09 (-0.55,0.37)	-0.05 (-0.35,0.25)	-0.03 (-0.37,0.31)	0.10 (-2.11,2.31)	Placebo	0.06 (-0.33,0.46)	0.16 (-0.16,0.48)
-0.34 (-0.86,0.18)	-0.16 (-0.54,0.21)	-0.16 (-0.71,0.40)	-0.12 (-0.53,0.30)	-0.09 (-0.52,0.34)	0.04 (-2.18,2.26)	-0.06 (-0.46,0.33)	Ipragliflozin	0.10 (-0.41,0.61)
-0.44 (-1.00,0.12)	-0.26 (-0.69,0.17)	-0.25 (-0.82,0.31)	-0.22 (-0.66,0.22)	-0.19 (-0.66,0.28)	-0.06 (-2.29,2.17)	-0.16 (-0.48,0.16)	-0.10 (-0.61,0.41)	Semaglutide

Table 18 Serum adiponectin

The columns present the row drug class compared to the column drug class. The rows present the row drug class compared to the column drug class. The effect estimates are expressed as mean difference and 95% confidence interval. For example, the mean difference in adiponectin for liraglutide therapy compared to placebo therapy is 3.03 μg/ml (95% confidence interval -0.43 μg/ml to 6.49 μg/ml). The mean difference in adiponectin for placebo therapy compared to liraglutide therapy is the inverse (-3.03 μg/ml (95% confidence interval -6.49 μg/ml to 0.43 μg/ml)).

Liraglutide	-3.03 (-6.49,0.43)	-4.25 (-7.94,-0.56)	-4.62 (-9.26,0.03)	-4.64 (-8.42,-0.86)	-10.21 (-16.24,-4.18)
3.03 (-0.43,6.49)	Placebo	-1.22 (-4.78,2.34)	-1.59 (-6.13,2.96)	-1.61 (-5.02,1.81)	-7.18 (-13.13,-1.23)
4.25 (0.56,7.94)	1.22 (-2.34,4.78)	Active control	-0.37 (-3.20,2.46)	-0.39 (-2.02,1.25)	-5.96 (-10.73,-1.19)
4.62 (-0.03,9.26)	1.59 (-2.96,6.13)	0.37 (-2.46,3.20)	Exenatide	-0.02 (-3.29,3.25)	-5.59 (-11.14,-0.05)
4.64 (0.86,8.42)	1.61 (-1.81,5.02)	0.39 (-1.25,2.02)	0.02 (-3.25,3.29)	Dapagliflozin	-5.57 (-10.61,-0.53)
10.21 (4.18,16.24)	7.18 (1.23,13.13)	5.96 (1.19,10.73)	5.59 (0.05,11.14)	5.57 (0.53,10.61)	Ipragliflozin

Table 19 Fasting blood glucose (FBG)

The columns present the row drug class compared to the column drug class. The rows present the row drug class compared to the column drug class. The effect estimates are expressed as mean difference and 95% confidence interval. For example, the mean difference in FBG for semaglutide therapy compared to liraglutide therapy is -0.57 mmol/L (95% confidence interval -2.29 mmol/L to 1.15 mmol/L). The mean difference in FBG for liraglutide therapy compared to semaglutide therapy is the inverse (0.57 mmol/L (95% confidence interval -1.15 mmol/L to 2.29 mmol/L)).

Semaglutide	0.57 (-1.15,2.29)	0.59 (-1.11,2.30)	0.72 (-1.06,2.51)	-0.98 (-15.49,13.54)	0.02 (-8.94,8.99)	0.99 (-0.77,2.74)	1.02 (-0.72,2.76)	1.02 (-0.68,2.72)	1.35 (-0.32,3.01)	1.40 (-0.34,3.14)	2.02 (-0.31,4.34)
-0.57 (-2.29,1.15)	Liraglutide	0.02 (-0.40,0.44)	0.15 (-0.33,0.64)	-1.55 (-15.97,12.87)	-0.55 (-9.36,8.26)	0.41 (-0.15,0.98)	0.45 (-0.15,1.04)	0.45 (0.11,0.79)	0.77 (0.35,1.19)	0.83 (0.19,1.47)	1.44 (-0.18,3.06)
-0.59 (-2.30,1.11)	-0.02 (-0.44,0.40)	Dapagliflozin	0.13 (-0.51,0.77)	-1.57 (-15.99,12.85)	-0.57 (-9.38,8.24)	0.39 (-0.16,0.95)	0.43 (-0.14,0.99)	0.43 (0.11,0.76)	0.75 (0.39,1.12)	0.81 (0.20,1.42)	1.42 (-0.19,3.04)
-0.72 (-2.51,1.06)	-0.15 (-0.64,0.33)	-0.13 (-0.77,0.51)	Diet and exercise	-1.70 (-16.13,12.73)	-0.70 (-9.52,8.12)	0.26 (-0.48,1.00)	0.29 (-0.47,1.06)	0.30 (-0.29,0.89)	0.62 (-0.02,1.26)	0.68 (-0.13,1.48)	1.29 (-0.40,2.98)
0.98 (-13.54,15.49)	1.55 (-12.87,15.97)	1.57 (-12.85,15.99)	1.70 (-12.73,16.13)	Luseogliflozin	1.00 (-15.89,17.89)	1.96 (-12.46,16.39)	1.99 (-12.43,16.42)	2.00 (-12.42,16.42)	2.32 (-12.10,16.74)	2.38 (-12.05,16.81)	2.99 (-11.51,17.50)
-0.02 (-8.99,8.94)	0.55 (-8.26,9.36)	0.57 (-8.24,9.38)	0.70 (-8.12,9.52)	-1.00 (-17.89,15.89)	Dulaglutide	0.96 (-7.85,9.78)	0.99 (-7.82,9.81)	1.00 (-7.80,9.80)	1.32 (-7.49,10.13)	1.38 (-7.44,10.20)	1.99 (-6.95,10.94)
-0.99 (-2.74,0.77)	-0.41 (-0.98,0.15)	-0.39 (-0.95,0.16)	-0.26 (-1.00,0.48)	-1.96 (-16.39,12.46)	-0.96 (-9.78,7.85)	Exenatide	0.03 (-0.67,0.73)	0.04 (-0.41,0.48)	0.36 (-0.20,0.91)	0.42 (-0.30,1.14)	1.03 (-0.61,2.68)
-1.02 (-2.76,0.72)	-0.45 (-1.04,0.15)	-0.43 (-0.99,0.14)	-0.29 (-1.06,0.47)	-1.99 (-16.42,12.43)	-0.99 (-9.81,7.82)	-0.03 (-0.73,0.67)	Ipragliflozin	0.01 (-0.54,0.55)	0.33 (-0.18,0.84)	0.38 (-0.33,1.10)	1.00 (-0.68,2.67)
-1.02 (-2.72,0.68)	-0.45 (-0.79,-0.11)	-0.43 (-0.76,-0.11)	-0.30 (-0.89,0.29)	-2.00 (-16.42,12.42)	-1.00 (-9.80,7.80)	-0.04 (-0.48,0.41)	-0.01 (-0.55,0.54)	Active control	0.32 (-0.02,0.66)	0.38 (-0.19,0.95)	0.99 (-0.59,2.58)
-1.35 (-3.01,0.32)	**-0.77 (-1.19,-0.35)**	**-0.75 (-1.12,-0.39)**	-0.62 (-1.26,0.02)	-2.32 (-16.74,12.10)	-1.32 (-10.13,7.49)	-0.36 (-0.91,0.20)	-0.33 (-0.84,0.18)	-0.32 (-0.66,0.02)	Placebo	0.06 (-0.44,0.55)	0.67 (-0.95,2.29)
-1.40 (-3.14,0.34)	-0.83 (-1.47,-0.19)	-0.81 (-1.42,-0.20)	-0.68 (-1.48,0.13)	-2.38 (-16.81,12.05)	-1.38 (-10.20,7.44)	-0.42 (-1.14,0.30)	-0.38 (-1.10,0.33)	-0.38 (-0.95,0.19)	-0.06 (-0.55,0.44)	Empagliflozin	0.61 (-1.07,2.30)
-2.02 (-4.34,0.31)	-1.44 (-3.06,0.18)	-1.42 (-3.04,0.19)	-1.29 (-2.98,0.40)	-2.99 (-17.50,11.51)	-1.99 (-10.94,6.95)	-1.03 (-2.68,0.61)	-1.00 (-2.67,0.68)	-0.99 (-2.58,0.59)	-0.67 (-2.29,0.95)	-0.61 (-2.30,1.07)	Tofogliflozin

Table 20 Postprandial blood glucose (PBG)

The columns present the row drug class compared to the column drug class. The rows present the row drug class compared to the column drug class. The effect estimates are expressed as mean difference and 95% confidence interval. For example, the mean difference in PBG for dapagliflozin therapy compared to liraglutide therapy is -0.44 mmol/L (95% confidence interval -2.61 mmol/L to 1.72 mmol/L). The mean difference in PBG for liraglutide therapy compared to dapagliflozin therapy is the inverse (0.44 mmol/L (95% confidence interval -1.72 mmol/L to 2.61 mmol/L)).

Dapagliflozin	0.44 (-1.72,2.61)	1.99 (-0.29,4.28)	2.14 (0.61,3.67)	2.14 (0.19,4.09)
-0.44 (-2.61,1.72)	Liraglutide	1.55 (0.04,3.06)	1.70 (-0.34,3.74)	1.70 (0.76,2.63)
-1.99 (-4.28,0.29)	-1.55 (-3.06,-0.04)	Exenatide	0.15 (-2.02,2.32)	0.15 (-1.04,1.33)
**-2.14 (-3.67,-0.61)**	-1.70 (-3.74,0.34)	-0.15 (-2.32,2.02)	Placebo	-0.00 (-1.82,1.81)
-2.14 (-4.09,-0.19)	-1.70 (-2.63,-0.76)	-0.15 (-1.33,1.04)	0.00 (-1.81,1.82)	Active control

Table 21 Glycosylated hemoglobin (HbA1c)

The columns present the row drug class compared to the column drug class. The rows present the row drug class compared to the column drug class. The effect estimates are expressed as mean difference and 95% confidence interval. For example, the mean difference in HbA1C for luseogliflozin therapy compared to semaglutide therapy is -0.12 % (95% confidence interval -0.93 % to 0.69 %). The mean difference in HbA1C for semaglutide therapy compared to luseogliflozin therapy is the inverse (0.12 % (95% confidence interval -0.69 % to 0.93 %)).

Luseogliflozin	0.12 (-0.69,0.93)	0.34 (-0.40,1.08)	0.40 (-0.65,1.45)	0.55 (-0.19,1.30)	0.64 (-0.13,1.42)	0.69 (-0.12,1.50)	0.70 (-0.01,1.41)	0.93 (0.07,1.79)	0.99 (0.09,1.89)	1.05 (0.30,1.80)
-0.12 (-0.93,0.69)	Semaglutide	0.21 (-0.21,0.64)	0.28 (-0.60,1.15)	0.43 (-0.00,0.86)	0.52 (0.01,1.03)	0.57 (0.06,1.08)	0.58 (0.18,0.98)	0.81 (0.21,1.40)	0.87 (0.18,1.55)	0.93 (0.63,1.23)
-0.34 (-1.08,0.40)	-0.21 (-0.64,0.21)	Dapagliflozin	0.06 (-0.74,0.87)	0.22 (-0.10,0.53)	0.31 (-0.08,0.69)	0.35 (-0.09,0.80)	0.36 (0.14,0.58)	0.59 (0.06,1.12)	0.65 (0.06,1.25)	0.72 (0.42,1.01)
-0.40 (-1.45,0.65)	-0.28 (-1.15,0.60)	-0.06 (-0.87,0.74)	Dulaglutide	0.15 (-0.66,0.96)	0.24 (-0.60,1.08)	0.29 (-0.58,1.17)	0.30 (-0.48,1.08)	0.53 (-0.39,1.45)	0.59 (-0.36,1.55)	0.65 (-0.17,1.47)
-0.55 (-1.30,0.19)	-0.43 (-0.86,0.00)	-0.22 (-0.53,0.10)	-0.15 (-0.96,0.66)	Liraglutide	0.09 (-0.30,0.49)	0.14 (-0.31,0.59)	0.15 (-0.09,0.38)	0.38 (-0.16,0.91)	0.44 (-0.17,1.04)	0.50 (0.19,0.81)
-0.64 (-1.42,0.13)	-0.52 (-1.03,-0.01)	-0.31 (-0.69,0.08)	-0.24 (-1.08,0.60)	-0.09 (-0.49,0.30)	Exenatide	0.05 (-0.46,0.56)	0.06 (-0.26,0.37)	0.29 (-0.30,0.87)	0.35 (-0.29,0.99)	0.41 (-0.00,0.82)
-0.69 (-1.50,0.12)	-0.57 (-1.08,-0.06)	-0.35 (-0.80,0.09)	-0.29 (-1.17,0.58)	-0.14 (-0.59,0.31)	-0.05 (-0.56,0.46)	Ipragliflozin	0.01 (-0.39,0.41)	0.24 (-0.38,0.86)	0.30 (-0.39,0.98)	0.36 (-0.04,0.77)
-0.70 (-1.41,0.01)	-0.58 (-0.98,-0.18)	-0.36 (-0.58,-0.14)	-0.30 (-1.08,0.48)	-0.15 (-0.38,0.09)	-0.06 (-0.37,0.26)	-0.01 (-0.41,0.39)	Active control	0.23 (-0.26,0.72)	0.29 (-0.26,0.85)	0.35 (0.09,0.61)
-0.93 (-1.79,-0.07)	-0.81 (-1.40,-0.21)	-0.59 (-1.12,-0.06)	-0.53 (-1.45,0.39)	-0.38 (-0.91,0.16)	-0.29 (-0.87,0.30)	-0.24 (-0.86,0.38)	-0.23 (-0.72,0.26)	Empagliflozin	0.06 (-0.68,0.80)	0.12 (-0.39,0.64)
-0.99 (-1.89,-0.09)	-0.87 (-1.55,-0.18)	-0.65 (-1.25,-0.06)	-0.59 (-1.55,0.36)	-0.44 (-1.04,0.17)	-0.35 (-0.99,0.29)	-0.30 (-0.98,0.39)	-0.29 (-0.85,0.26)	-0.06 (-0.80,0.68)	Tofogliflozin	0.06 (-0.55,0.67)
**-1.05 (-1.80,-0.30)**	**-0.93 (-1.23,-0.63)**	**-0.72 (-1.01,-0.42)**	-0.65 (-1.47,0.17)	**-0.50 (-0.81,-0.19)**	-0.41 (-0.82,0.00)	-0.36 (-0.77,0.04)	**-0.35 (-0.61,-0.09)**	-0.12 (-0.64,0.39)	-0.06 (-0.67,0.55)	Placebo

Table 22 Glucose and homeostasis model assessment (HOMA-IR)

The columns present the row drug class compared to the column drug class. The rows present the row drug class compared to the column drug class. The effect estimates are expressed as mean difference and 95% confidence interval. For example, the mean difference in HOMA-IR for diet and exercise therapy compared to liraglutide therapy is -0.69 (95% confidence interval -2.11 to 0.73). The mean difference in HOMA-IR for liraglutide therapy compared to diet and exercise therapy is the inverse (0.69 (95% confidence interval -0.73 to 2.11)).

Diet and exercise	0.69 (-0.73,2.11)	1.12 (-0.54,2.78)	1.49 (-0.30,3.28)	1.63 (-0.26,3.52)	1.62 (0.04,3.21)	1.68 (-0.20,3.56)	1.97 (0.09,3.85)	1.97 (0.33,3.62)
-0.69 (-2.11,0.73)	Liraglutide	0.43 (-0.44,1.29)	0.80 (-0.29,1.89)	0.94 (-0.31,2.20)	0.93 (0.22,1.65)	0.99 (-0.24,2.22)	1.28 (0.05,2.51)	1.28 (0.44,2.12)
-1.12 (-2.78,0.54)	-0.43 (-1.29,0.44)	Dapagliflozin	0.37 (-0.62,1.36)	0.52 (-0.65,1.68)	0.51 (-0.04,1.05)	0.56 (-0.56,1.69)	0.85 (-0.26,1.97)	0.85 (0.18,1.53)
-1.49 (-3.28,0.30)	-0.80 (-1.89,0.29)	-0.37 (-1.36,0.62)	Ipragliflozin	0.14 (-1.22,1.51)	0.13 (-0.76,1.03)	0.19 (-1.05,1.44)	0.48 (-0.77,1.74)	0.48 (-0.38,1.35)
-1.63 (-3.52,0.26)	-0.94 (-2.20,0.31)	-0.52 (-1.68,0.65)	-0.14 (-1.51,1.22)	Exenatide	-0.01 (-1.04,1.02)	0.05 (-1.45,1.54)	0.34 (-1.14,1.81)	0.34 (-0.86,1.53)
-1.62 (-3.21,-0.04)	-0.93 (-1.65,-0.22)	-0.51 (-1.05,0.04)	-0.13 (-1.03,0.76)	0.01 (-1.02,1.04)	Active control	0.06 (-1.02,1.14)	0.35 (-0.70,1.40)	0.35 (-0.25,0.95)
-1.68 (-3.56,0.20)	-0.99 (-2.22,0.24)	-0.56 (-1.69,0.56)	-0.19 (-1.44,1.05)	-0.05 (-1.54,1.45)	-0.06 (-1.14,1.02)	Semaglutide	0.29 (-1.01,1.59)	0.29 (-0.61,1.19)
-1.97 (-3.85,-0.09)	-1.28 (-2.51,-0.05)	-0.85 (-1.97,0.26)	-0.48 (-1.74,0.77)	-0.34 (-1.81,1.14)	-0.35 (-1.40,0.70)	-0.29 (-1.59,1.01)	Empagliflozin	0.00 (-0.94,0.94)
**-1.97 (-3.62,-0.33)**	**-1.28 (-2.12,-0.44)**	**-0.85 (-1.53,-0.18)**	-0.48 (-1.35,0.38)	-0.34 (-1.53,0.86)	-0.35 (-0.95,0.25)	-0.29 (-1.19,0.61)	-0.00 (-0.94,0.94)	Placebo

Appendix 10 Funnel plots for each outcome

Figure 1 Funnel plot for alanine aminotransferase (ALT)

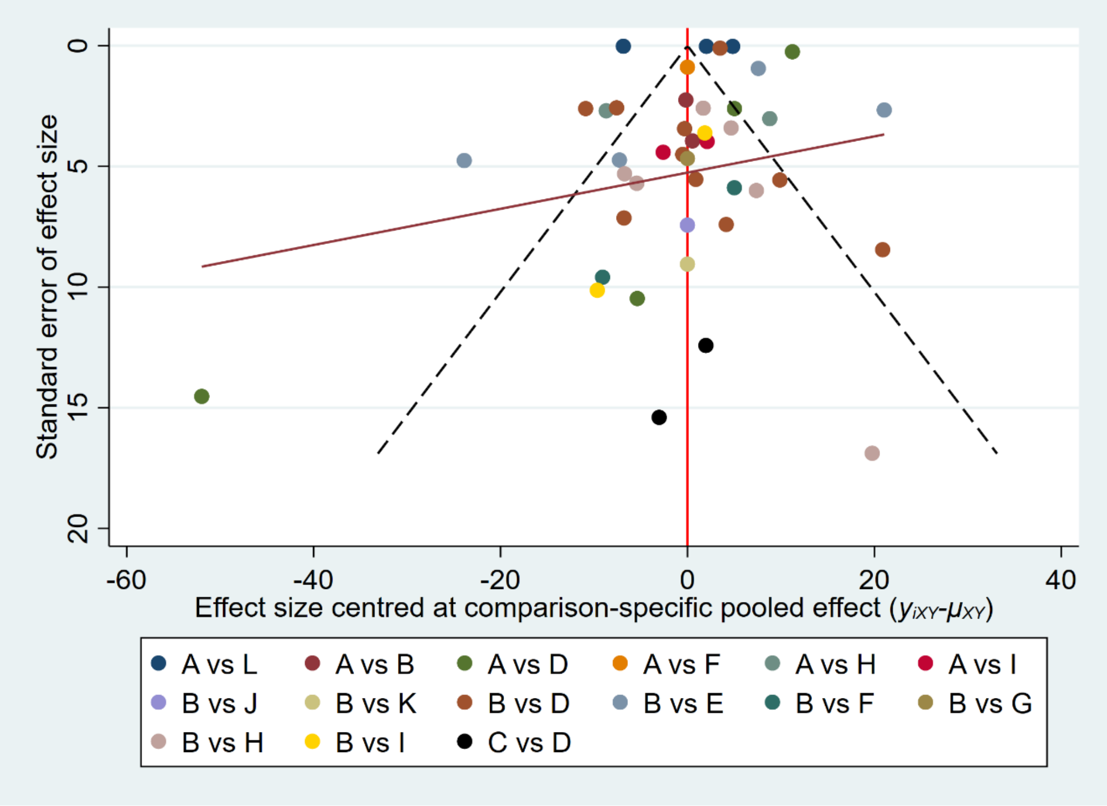

Figure 2 Funnel plot for aspartate aminotransferase (AST)

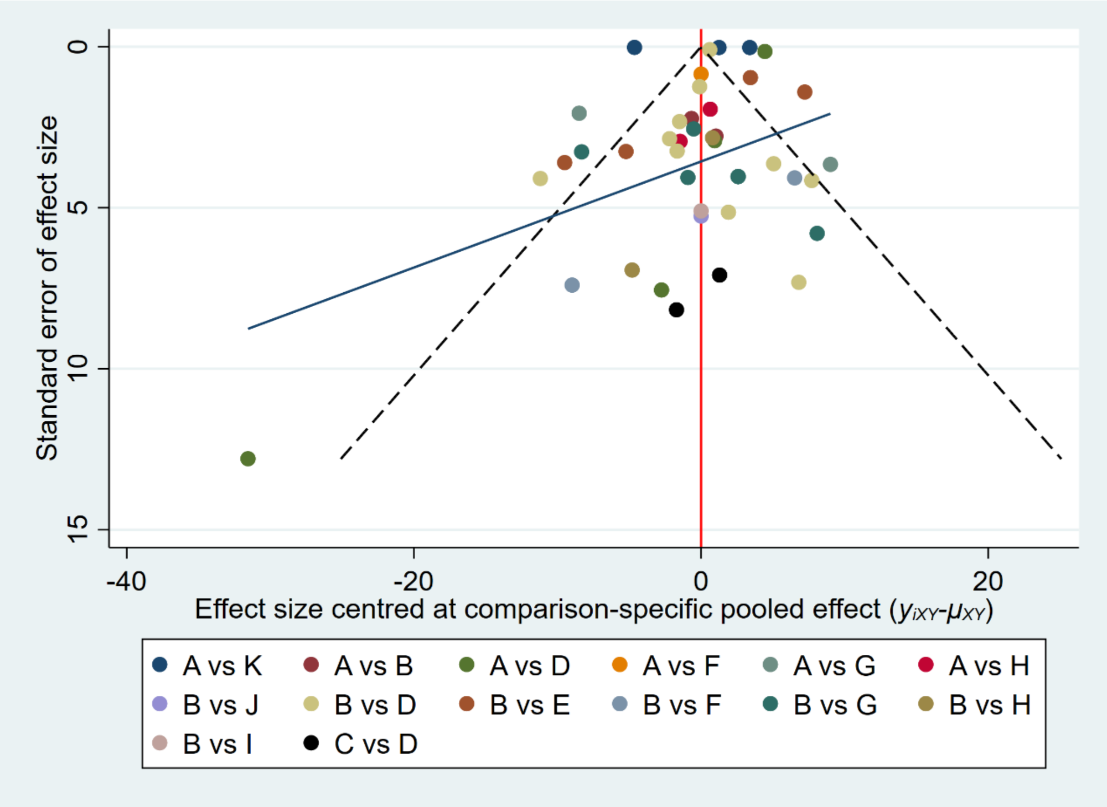

Figure 3 Funnel plot for γ-glutamyl transferase (GGT)

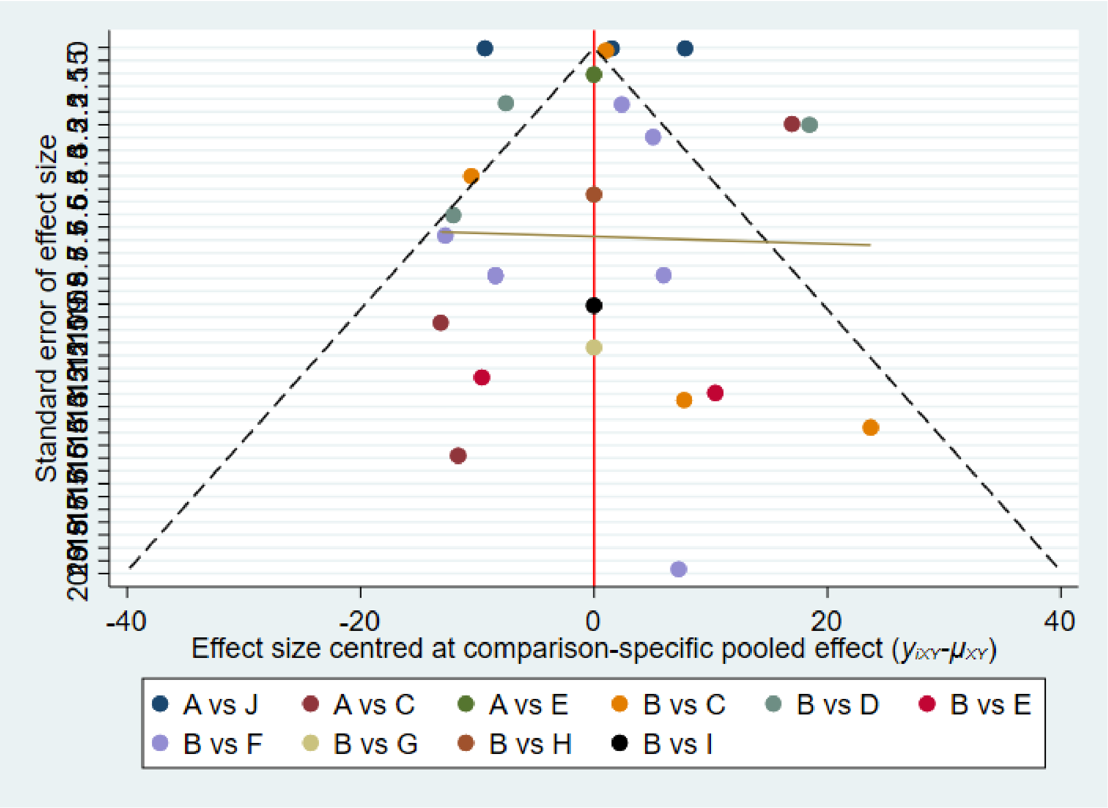

Figure 4 Funnel plot for subcutaneous adipose tissue (SAT)

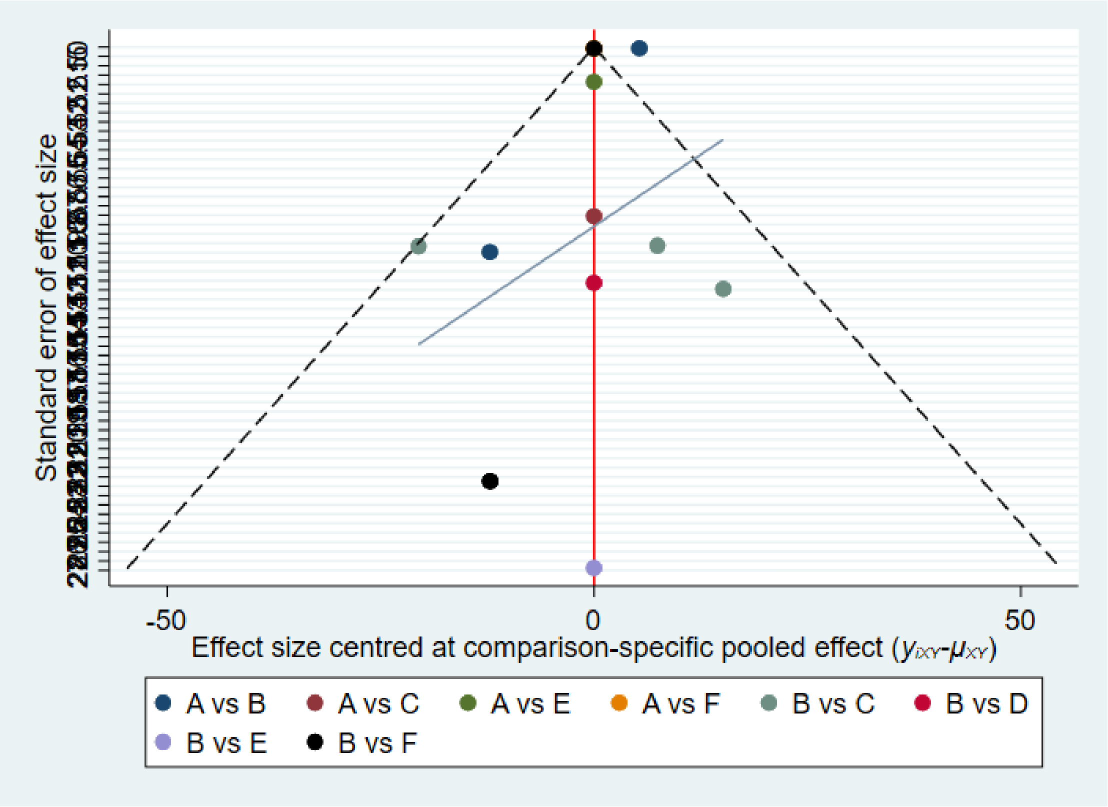

Figure 5 Funnel plot for visceral adipose tissue (VAT)

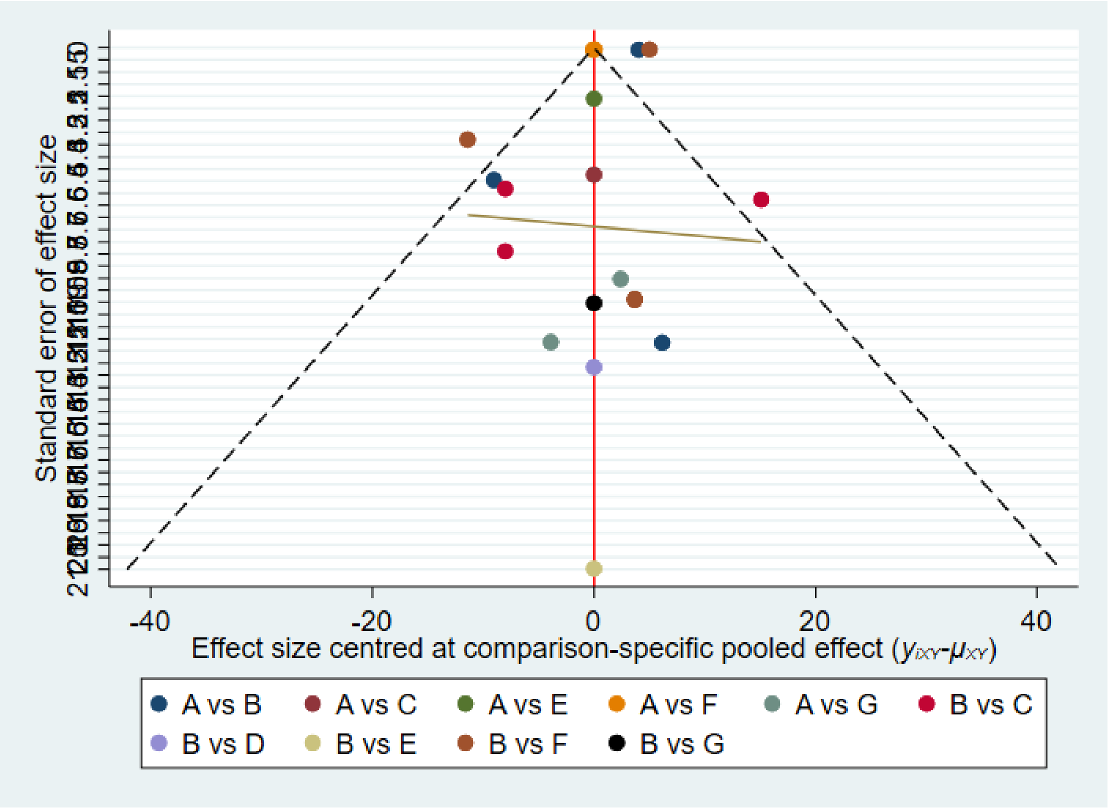

Figure 6 Funnel plot for liver fat fraction (LFF)

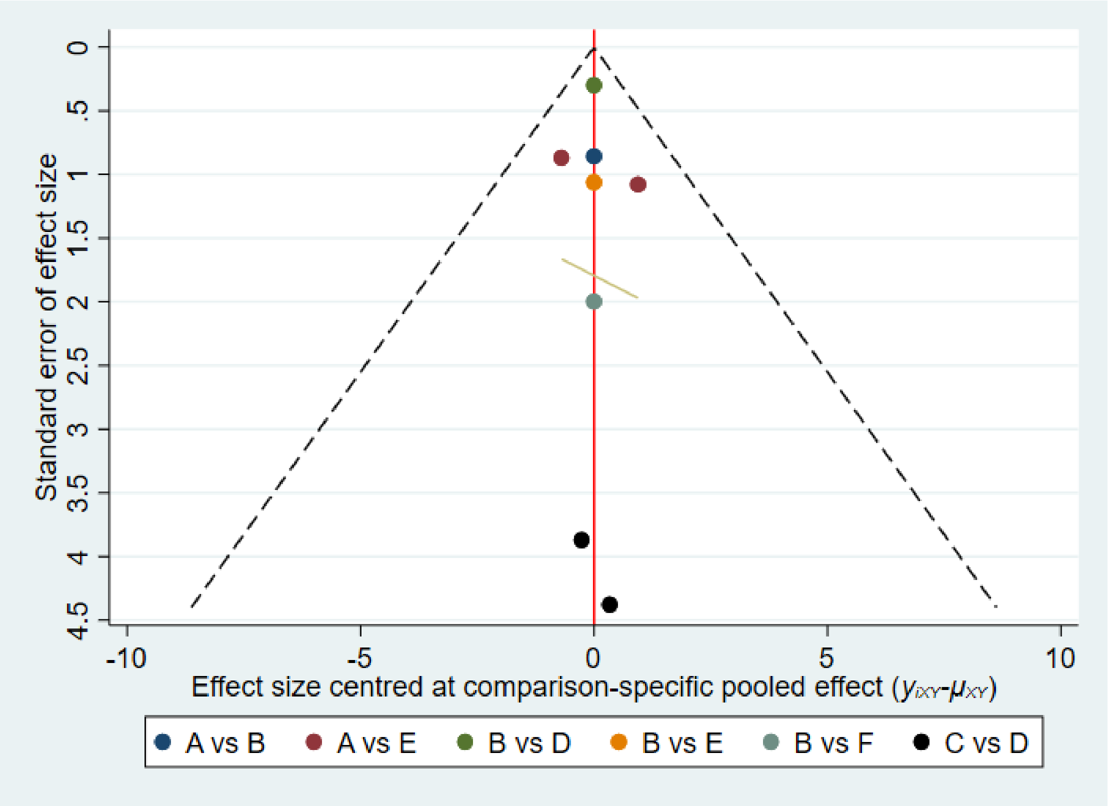

Figure 7 Funnel plot for controlled attenuation parameter (CAP)

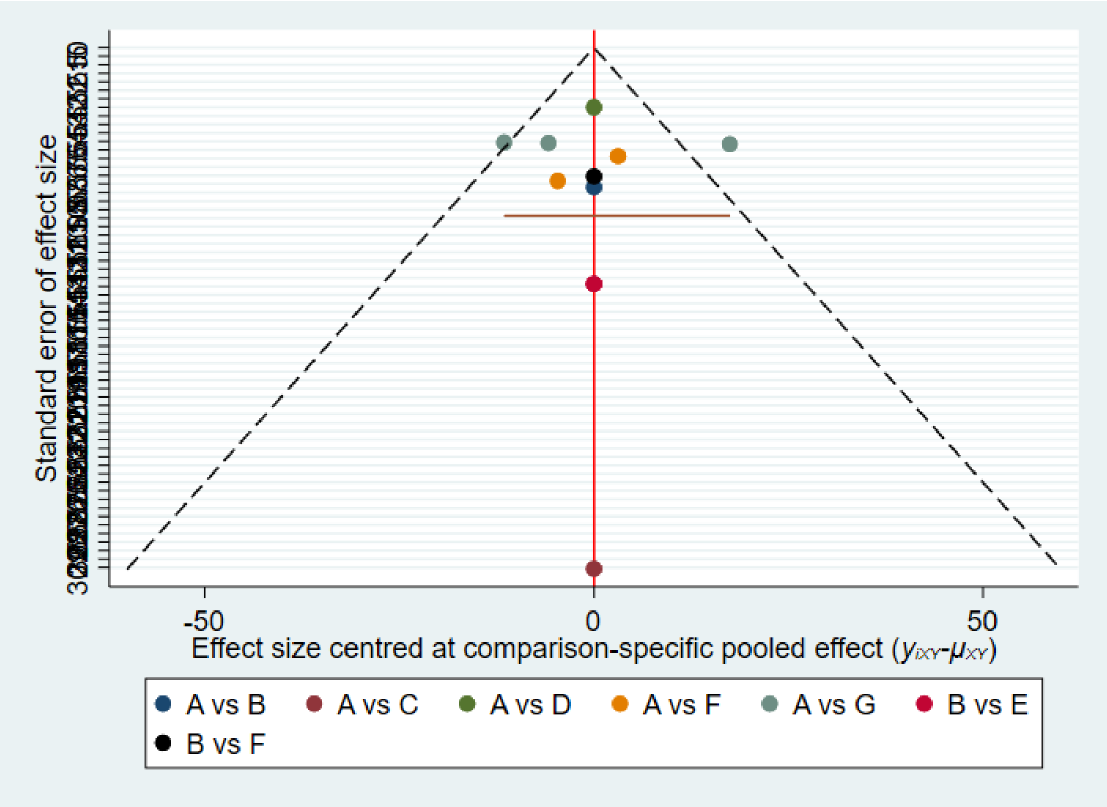

Figure 8 Funnel plot for liver stiffness measurement (LSM)

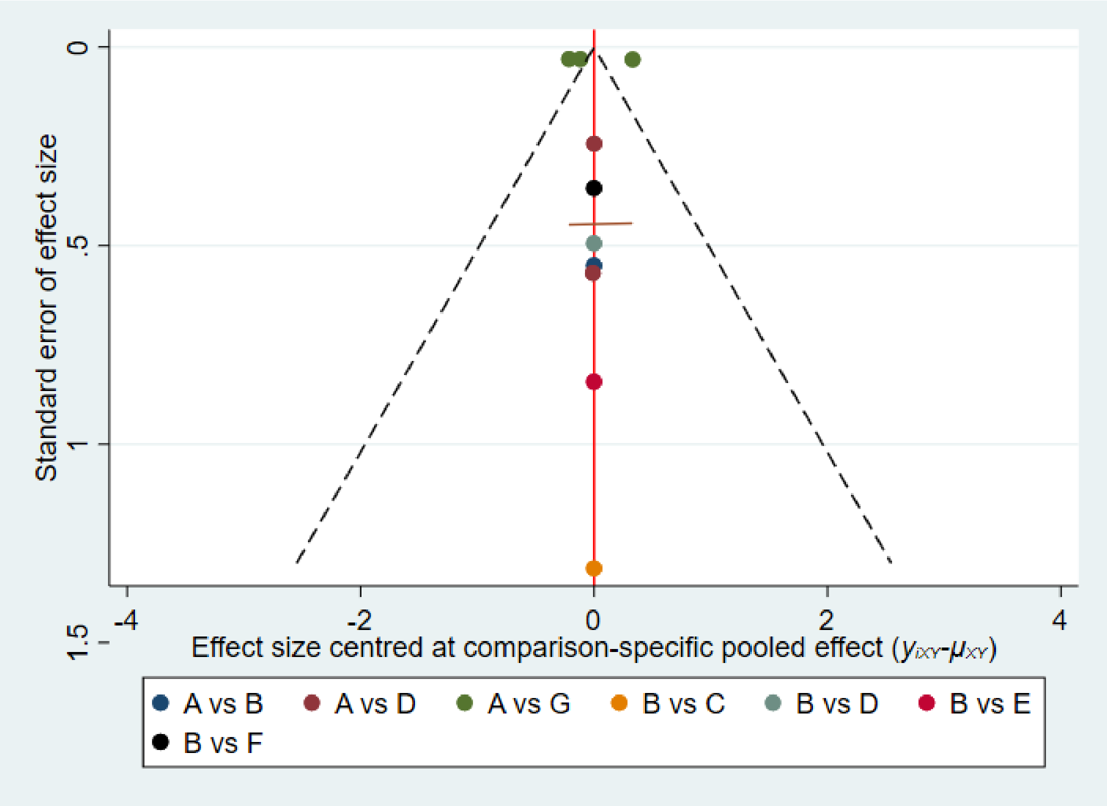

Figure 9 Funnel plot for body weight (BW)

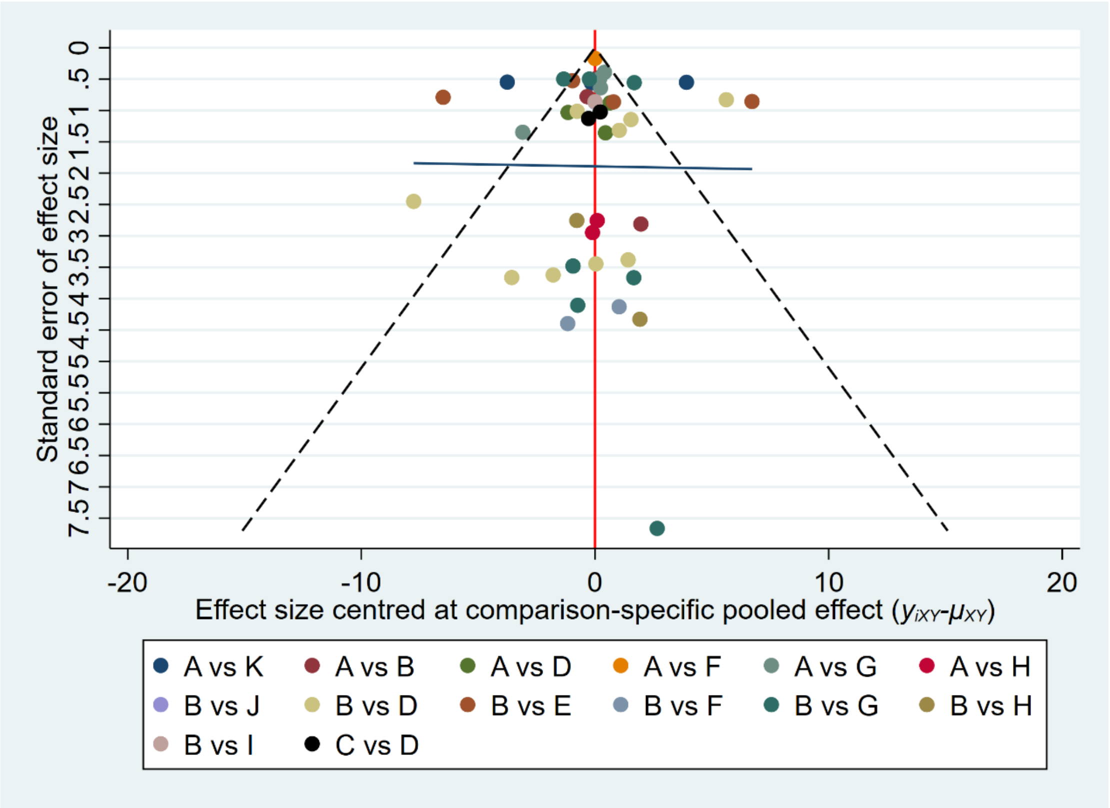

Figure 10 Funnel plot for body mass index (BMI)

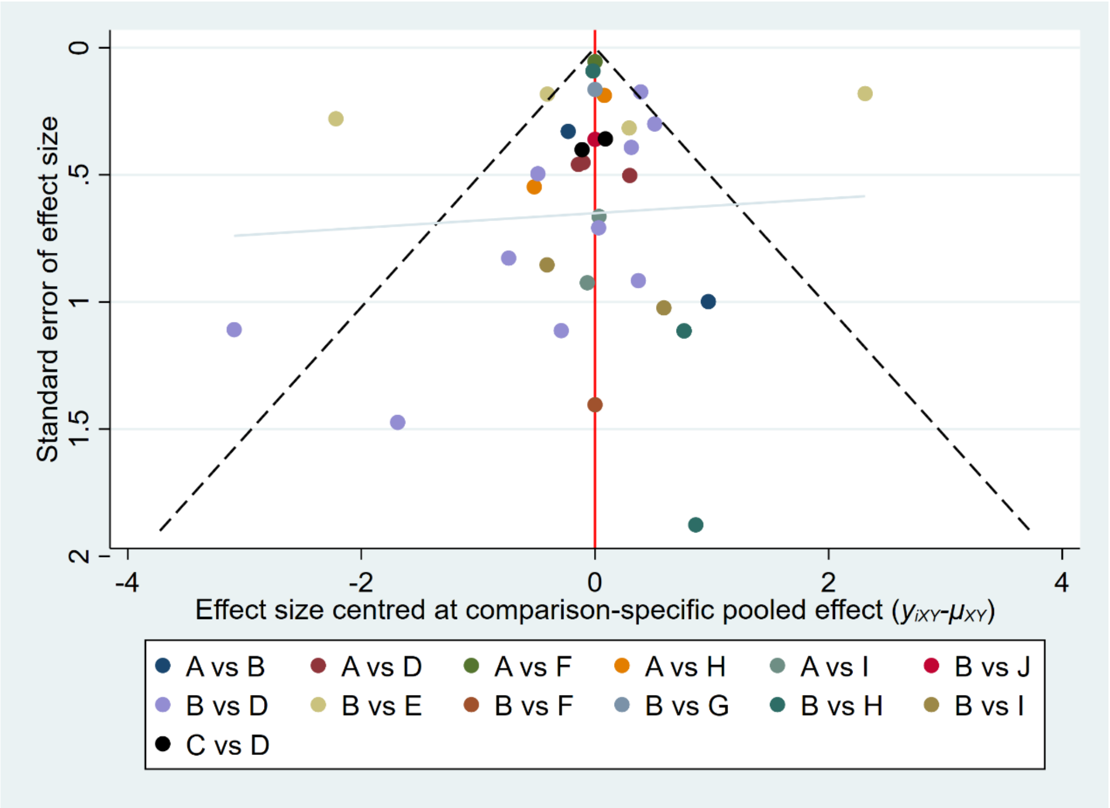

Figure 11 Funnel plot for waist circumference (WC)

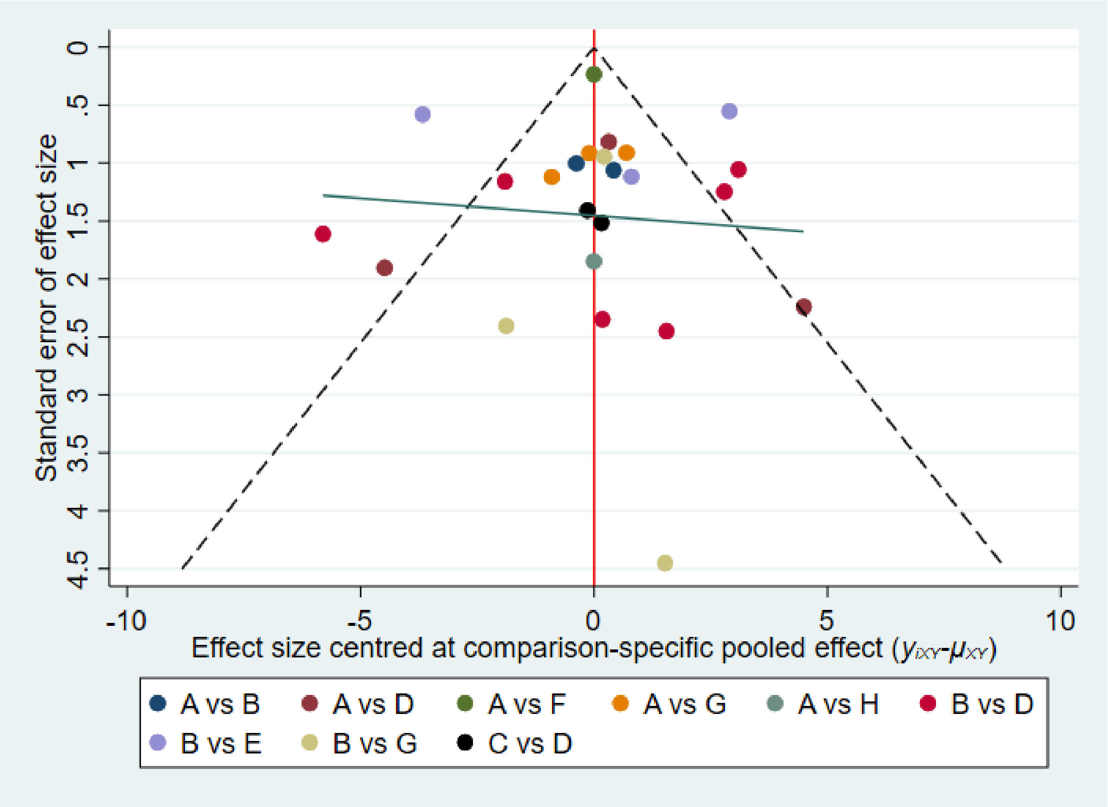

Figure 12 Funnel plot for systobic blood pressure (SBP)

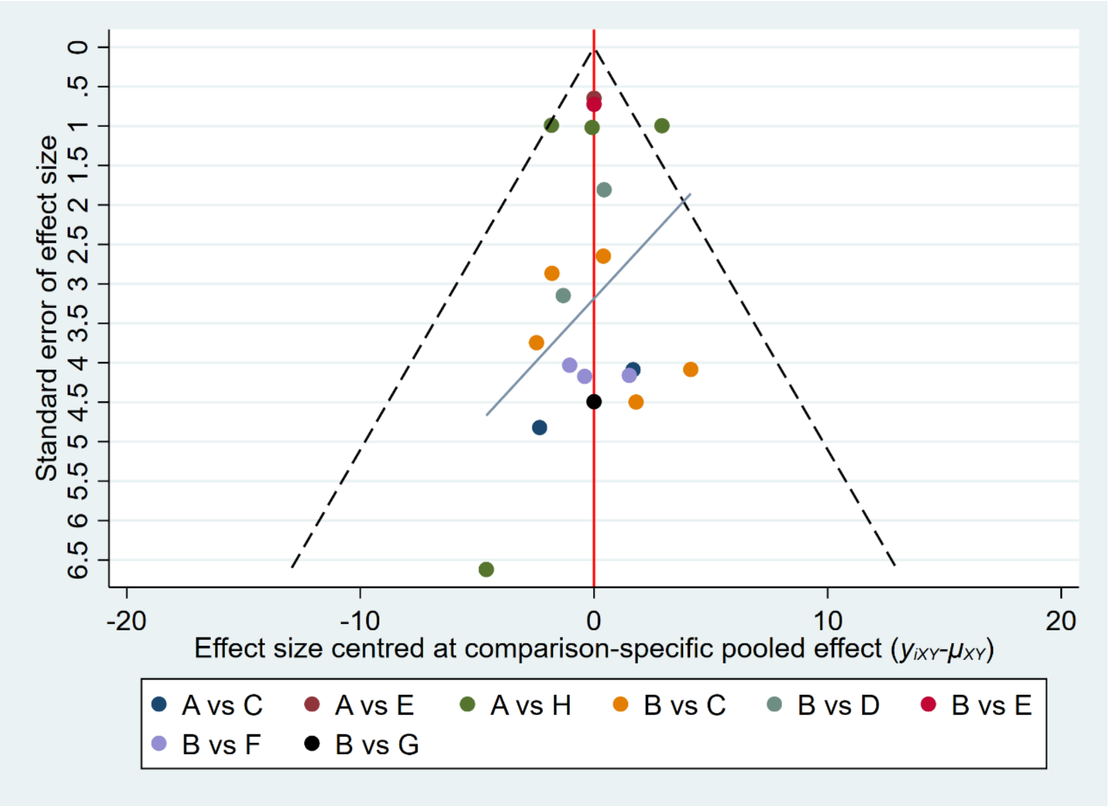

Figure 13 Funnel plot for diastolic blood pressure (DBP)

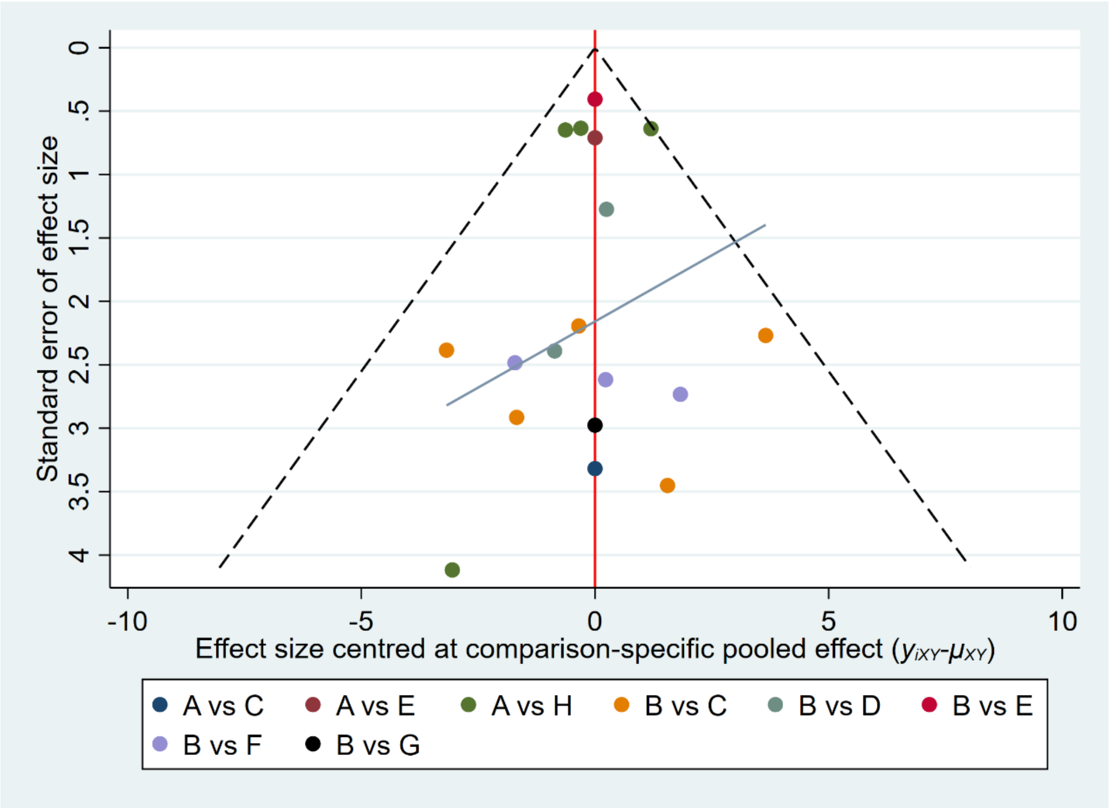

Figure 14 Funnel plot for blood lipids [total cholesterol(TC)

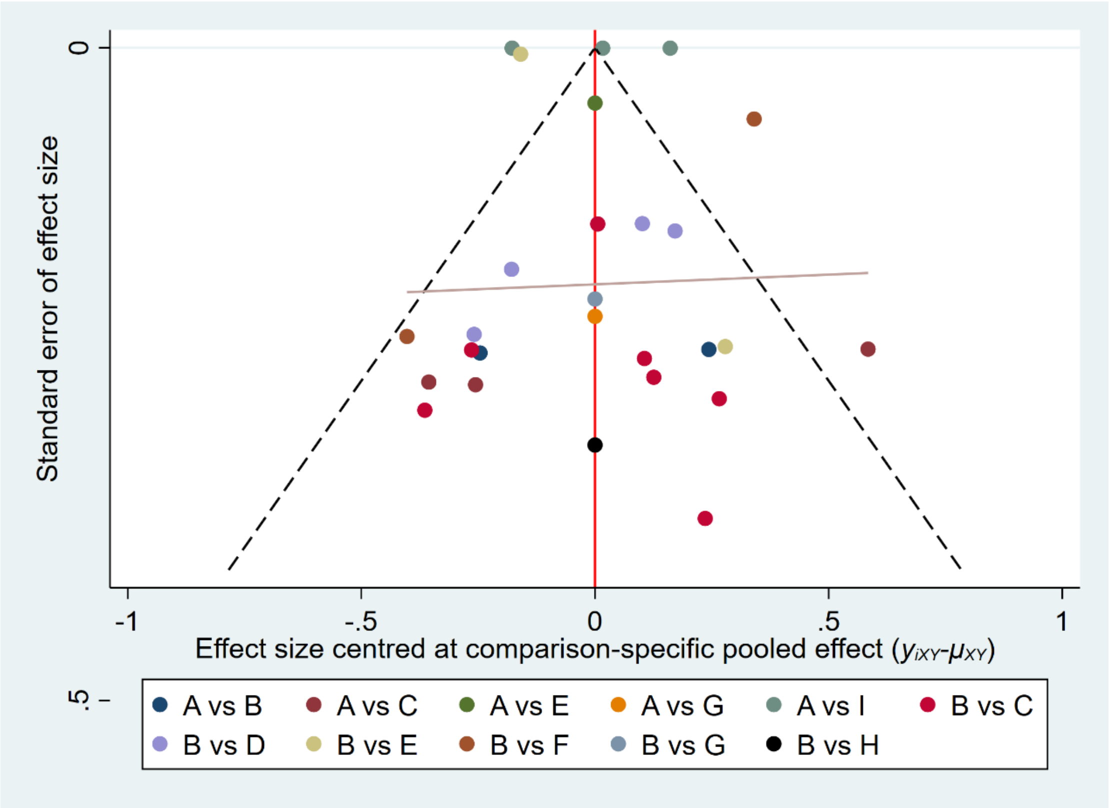

Figure 15 Funnel plot for triglycerides (TG)

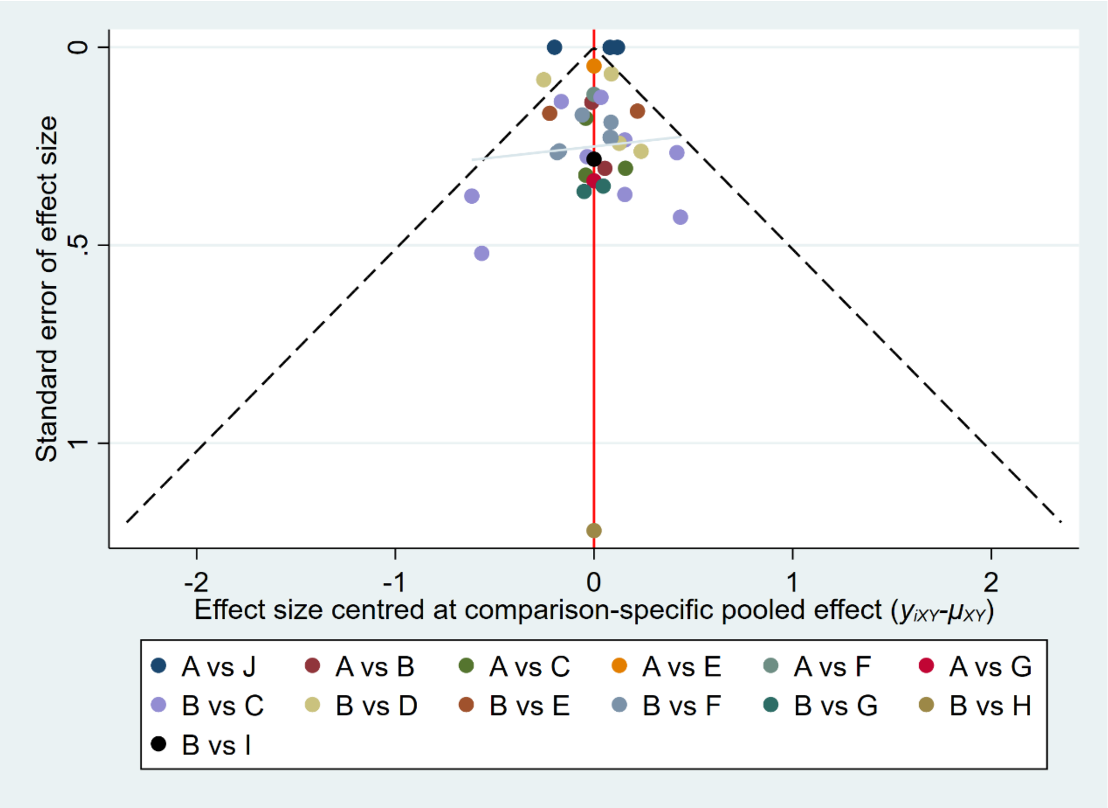

Figure 16 Funnel plot for high density lipoprotein-cholesterol (HDL-C)

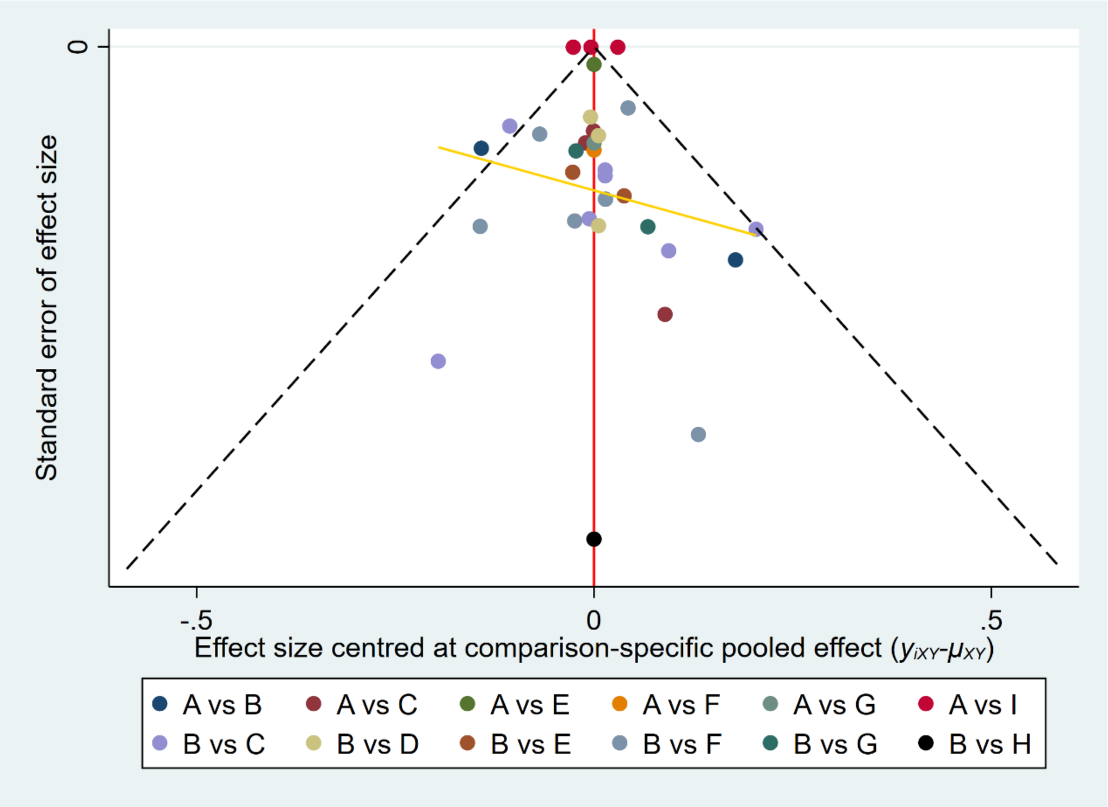

Figure 17 Funnel plot for low density lipoprotein-cholesterol (LDL-C)

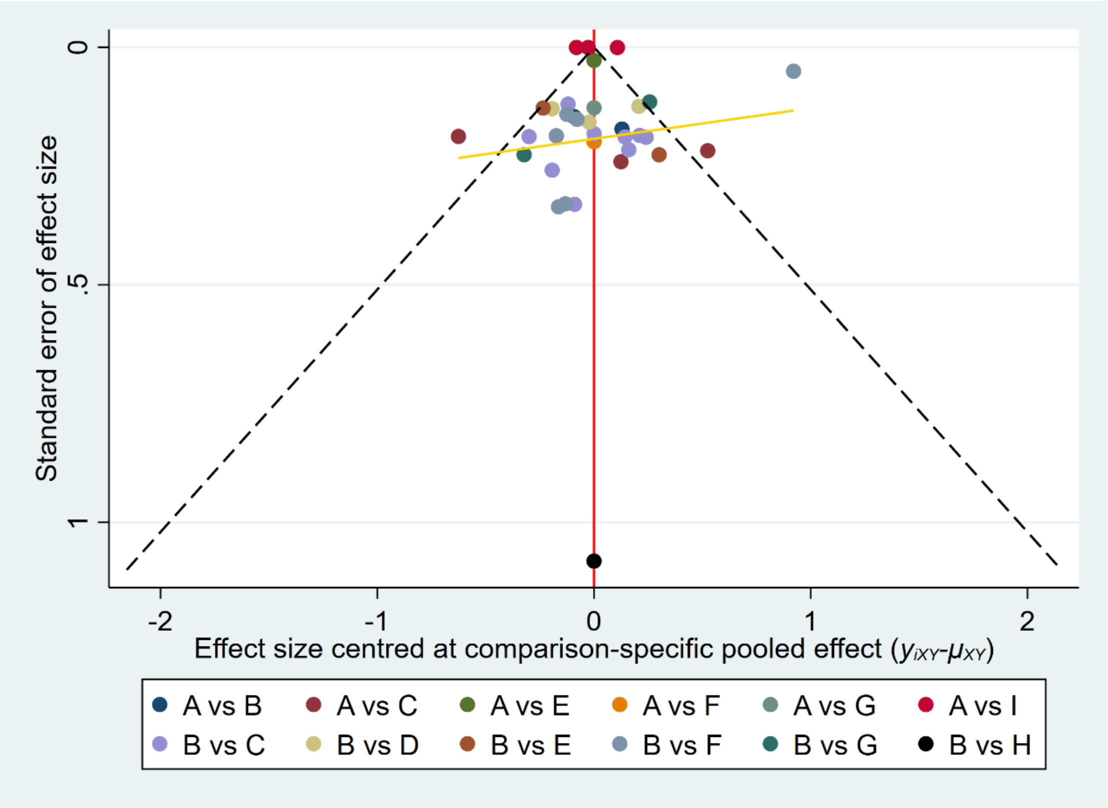

Figure 18 Funnel plot for serum adiponectin

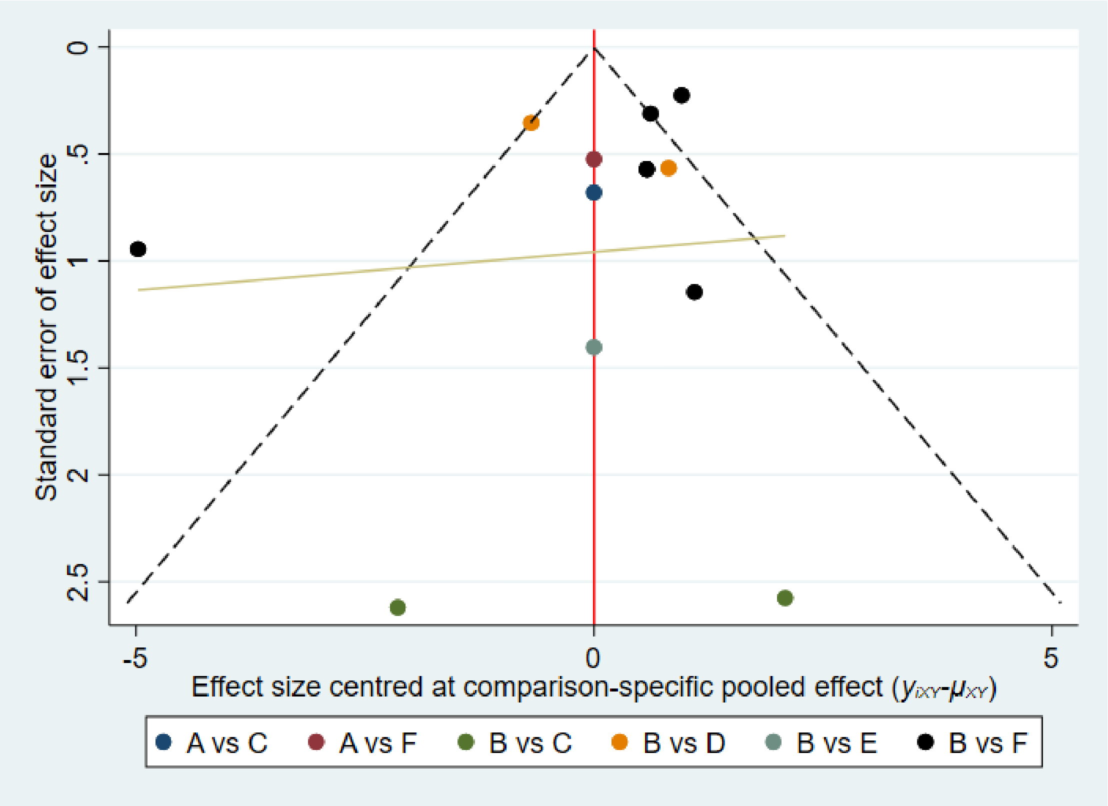

Figure 19 Funnel plot for fasting blood glucose (FBG)

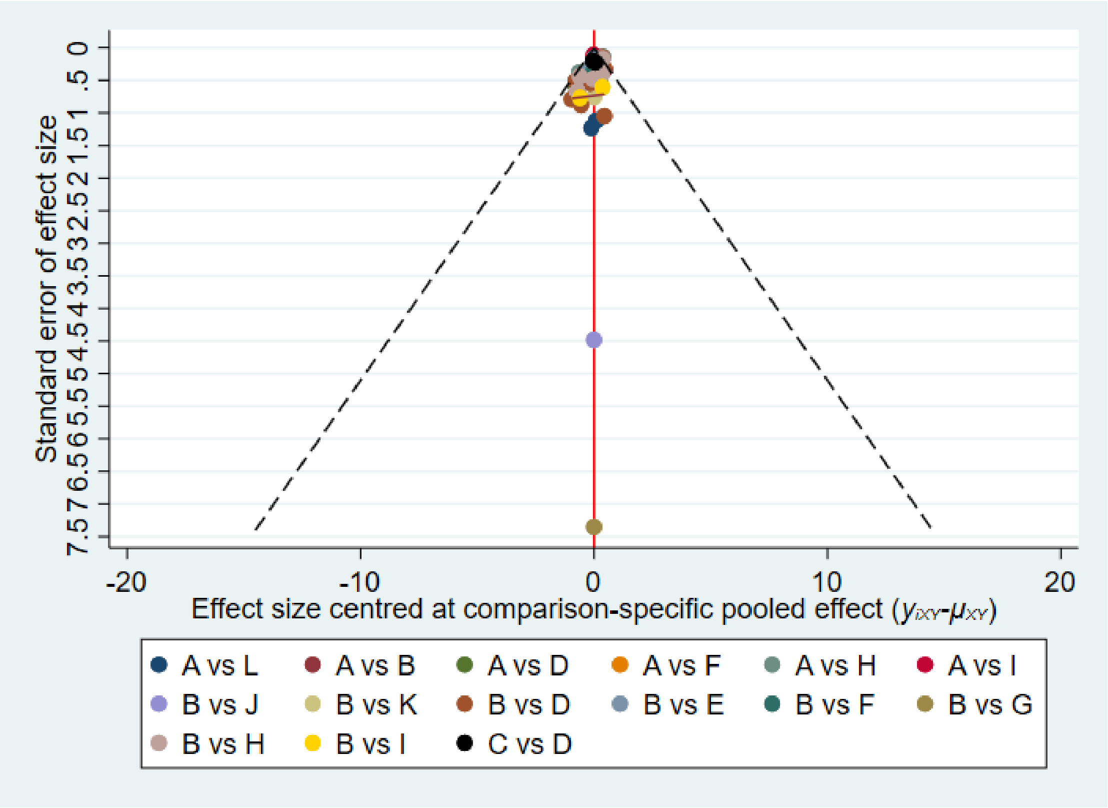

Figure 20 Funnel plot for postprandial blood glucose (PBG)

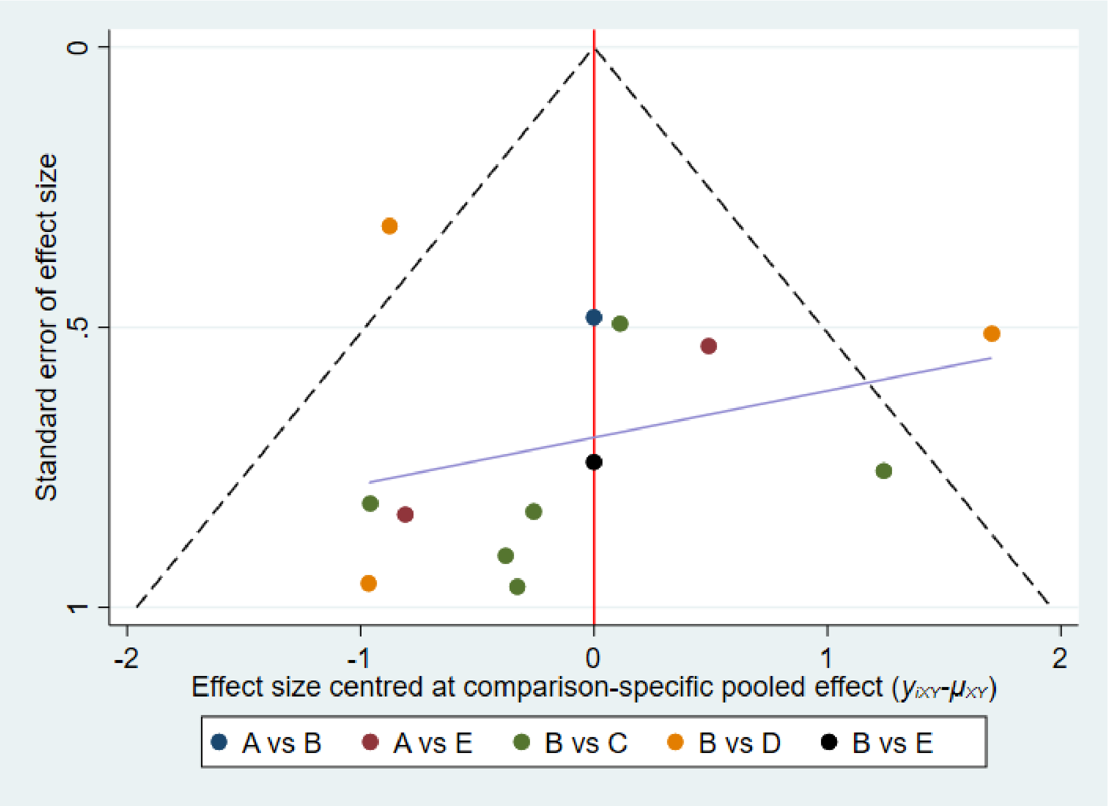

Figure 21 Funnel plot for glycosylated hemoglobin (HbA1c)

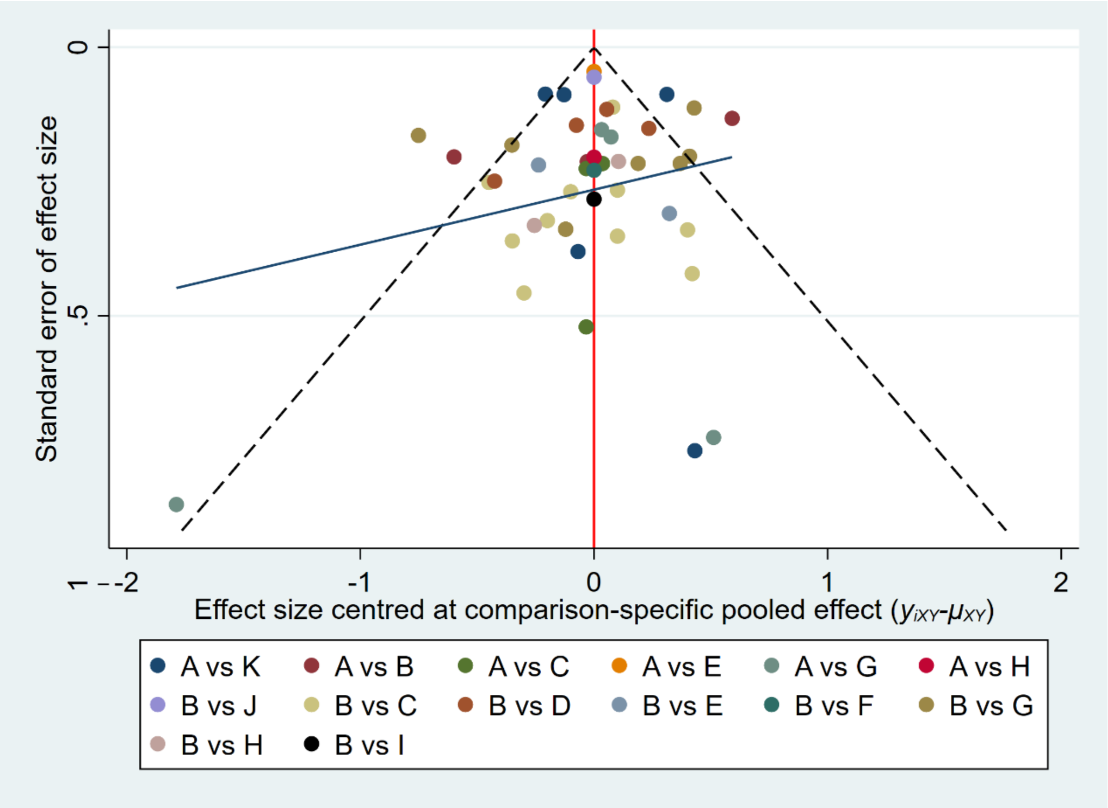

Figure 22 Funnel plot for glucose and homeostasis model assessment (HOMA-IR)

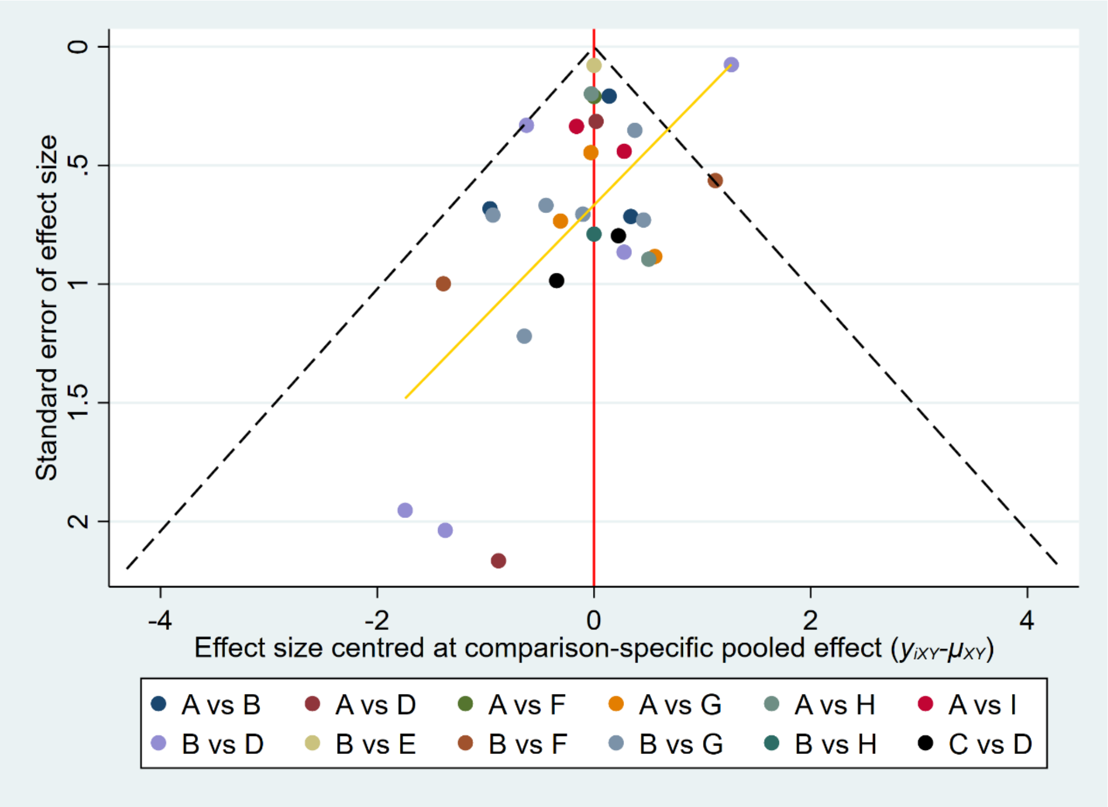

Appendix 11 GRADE summary of findings for SGLT-2 inhibitors and GLP-1 receptor agonists compared to placebo or each other

Table 1 Alanine aminotransferase (ALT)

**Comparison**	**Mean difference (95% CI)**	**Certainty in treatment effects （GRADE）**	**Plain text summary**
Liraglutide	-8.30 U/L (-16.16, -0.43) ⊕⊕⊕⊕	High	Liraglutide therapy could reduce ALT levels.
Exenatide	-9.65 U/L (-21.65,2.35) ⊕⊕⊖⊖	Low due to serious risk of bias and indirectness	Exenatide therapy may have no effect on ALT levels.
Dulaglutide	-16.31 U/L (-40.22,7.60) ⊕⊕⊕⊖	Moderate due to serious indirectness	Dulaglutide therapy probably has no effect on ALT levels.
Semaglutide	-14.70 U/L (-24.79,-4.61) ⊕⊕⊕⊕	High	Semaglutide therapy could reduce ALT levels.
Dapagliflozin	-9.94 U/L (-18.42,-1.46) ⊕⊕⊕⊕	High	Dapagliflozin therapy could reduce ALT levels.
Empagliflozin	-5.37 U/L (-17.17,6.43) ⊕⊕⊕⊕	High	Empagliflozin therapy has no effect on ALT levels.
Ipragliflozin	-8.09 U/L (-20.72,4.54) ⊕⊕⊖⊖	Low due to serious risk of bias and inconsistency	Ipragliflozin therapy may have no effect on ALT levels.
Tofogliflozin	6.69 U/L (-19.27,32.65)	Moderate due to	Tofogliflozin therapy probably

	⊕⊕⊕⊖	serious indirectness	has no effect on ALT levels.
Luseogliflozin	0.69 U/L (-20.37,21.75) ⊕⊕⊖⊖	Low due to serious risk of bias and inconsistency	Luseogliflozin therapy may have no effect on ALT levels.

CI= confidence interval.

Table 2 Aspartate aminotransferase (AST)

**Comparison**	**Mean difference (95% CI)**	**Certainty in treatment effects**	**Plain text summary**
Liraglutide	-4.60 U/L (-9.48,0.29) ⊕⊕⊕⊕	High	Liraglutide therapy has no effect on AST levels.
Exenatide	-8.50 U/L (-15.70,-1.29) ⊕⊕⊖⊖	Low due to serious risk of bias and indirectness	Exenatide therapy may reduce AST levels.
Dulaglutide	-13.34 U/L (-28.22,1.53) ⊕⊕⊕⊖	Moderate due to serious indirectness	Dulaglutide therapy probably has no effect on AST levels.
Semaglutide	-9.32 U/L (-15.12,-3.52) ⊕⊕⊕⊕	High	Semaglutide therapy could reduce AST levels.
Dapagliflozin	-6.70 U/L (-12.03,-1.37) ⊕⊕⊕⊖	Moderate due to serious inconsistency	Dapagliflozin therapy probably reduces AST levels.
Empagliflozin	-5.08 U/L (-12.07,1.92) ⊕⊕⊕⊕	High	Empagliflozin therapy has no effect on AST levels.
Ipragliflozin	-7.08 U/L (-	Low due to serious	Ipragliflozin therapy may have no

	14.76,0.60) ⊕⊕⊖⊖	risk of bias and inconsistency	effect on AST levels.
Tofogliflozin	8.06 U/L (-7.03,23.14) ⊕⊕⊕⊖	Moderate due to serious indirectness	Tofogliflozin therapy probably has no effect on AST levels.

CI= confidence interval.

Table 3 γ-glutamyl transferase (GGT)

Comparison	Mean difference (95% CI)	Certainty in treatment effects	Plain text summary
Liraglutide	-7.29 U/L (-19.88,5.30) ⊕⊕⊕⊕	High	Liraglutide therapy has no effect on GGT levels.
Exenatide	-7.29 U/L (-26.43,11.85) ⊕⊕⊖⊖	Low due to serious risk of bias and indirectness	Exenatide therapy may have no effect on GGT levels.
Dulaglutide	-18.70 U/L (-45.13,7.73) ⊕⊕⊕⊖	Moderate due to serious indirectness	Dulaglutide therapy probably has no effect on GGT levels.
Semaglutide	-16.56 U/L (-27.30,-5.82) ⊕⊕⊕⊕	High	Semaglutide therapy could reduce GGT levels.
Dapagliflozin	-13.82 U/L (-31.20,3.57) ⊕⊕⊖⊖	Low due to serious risk of bias and indirectness	Dapagliflozin therapy may have no effect on GGT levels.
Empagliflozin	-16.60 U/L (-49.74,16.53) ⊕⊕⊕⊖	Moderate due to serious indirectness	Empagliflozin therapy probably has no effect on GGT levels.

Ipragliflozin	-10.37 U/L (-26.04,5.31) ⊕⊕⊕⊖	Moderate due to serious risk of bias	Ipragliflozin therapy probably has no effect on GGT levels.
Tofogliflozin	23.80 U/L (-7.21,54.80) ⊕⊕⊕⊖	Moderate due to serious indirectness	Tofogliflozin therapy probably has no effect on GGT levels.

CI= confidence interval.

Table 4 Subcutaneous adipose tissue (SAT)

**Comparison**	**Mean difference (95% CI)**	**Certainty in treatment effects**	**Plain text summary**
Liraglutide	-30.27 cm^2 (-41.54,-19.01) ⊕⊕⊕⊕	High	Liraglutide therapy could reduce subcutaneous adipose tissue.
Exenatide	-30.93 cm^2 (-55.67,-6.20) ⊕⊖⊖⊖	Very low due to very serious risk of bias and serious indirectness	Whether exenatide therapy has an effect on subcutaneous adipose tissue is uncertain.
Dapagliflozin	-0.26 cm^2 (-0.36,-0.17) ⊕⊕⊕⊖	Moderate due to serious risk of bias	Dapagliflozin therapy probably reduces subcutaneous adipose tissue.
Ipragliflozin	-7.96 cm^2 (-11.60,-4.33) ⊕⊕⊕⊖	Moderate due to serious risk of bias	Ipragliflozin therapy probably reduces subcutaneous adipose tissue.

CI= confidence interval.

Table 5 Visceral adipose tissue (VAT)

**Comparison**	**Mean difference**	**Certainty in**	**Plain text summary**

	(95% CI)	treatment effects	
Liraglutide	-30.12 cm^2 (-45.36,-14.89) ⊕⊕⊕⊖	Moderate due to serious inconsistency	Liraglutide therapy probably reduces visceral adipose tissue.
Exenatide	-40.26 cm^2 (-74.32,-6.21) ⊕⊖⊖⊖	Very low due to very serious risk of bias and serious indirectness	Whether exenatide therapy has an effect on visceral adipose tissue is uncertain.
Dapagliflozin	-6.96 cm^2 (-18.37,4.46) ⊕⊕⊖⊖	Low due to serious risk of bias and inconsistency	Dapagliflozin therapy may have no effect on visceral adipose tissue.
Empagliflozin	-11.45 cm^2 (-30.12,7.22) ⊕⊕⊕⊕	High	Empagliflozin therapy has no effect on visceral adipose tissue.
Ipragliflozin	-25.13 cm^2 (-44.49,-5.76) ⊕⊕⊖⊖	Low due to serious risk of bias and inconsistency	Ipragliflozin therapy may reduce visceral adipose tissue.

CI= confidence interval.

Table 6 Liver fat fraction (LFF)

**Comparison**	**Mean difference (95% CI)**	**Certainty in treatment effects**	**Plain text summary**
Liraglutide	-5.16 % (-7.51,-2.81) ⊕⊕⊖⊖	Low due to serious risk of bias and indirectness	Liraglutide therapy may reduce liver fat fraction.
Dapagliflozin	-0.95 % (-2.52,0.62) ⊕⊕⊕⊖	Moderate due to serious inconsistency	Dapagliflozin therapy probably has no effect on liver fat fraction.

Tofogliflozin	1.08 % (-3.45,5.61) ⊕⊕⊕⊖	Moderate due to serious indirectness	Tofogliflozin therapy probably has no effect on liver fat fraction.

CI= confidence interval.

Table 7 Controlled attenuation parameter (CAP)

**Comparison**	**Mean difference (95% CI)**	**Certainty in treatment effects**	**Plain text summary**
Liraglutide	-16.70 db/m (-80.22,46.82) ⊕⊕⊕⊕	High	Liraglutide therapy has no effect on CAP levels.
Semaglutide	-15.57 db/m (-29.29,-1.85) ⊕⊕⊕⊕	High	Semaglutide therapy could reduce CAP levels.
Dapagliflozin	-38.86 db/m (-73.39,-4.33) ⊕⊕⊖⊖	Low due to serious risk of bias and indirectness	Dapagliflozin therapy may reduce CAP levels.
Empagliflozin	-9.04 db/m (-26.88,8.81) ⊕⊕⊕⊕	High	Empagliflozin therapy has no effect on CAP levels.
Ipragliflozin	-19.70 db/m (-41.86,2.46) ⊕⊕⊖⊖	Low due to very serious risk of bias	Ipragliflozin therapy may have no effect on CAP levels.

CI= confidence interval.

Table 8 Liver stiffness measurement (LSM)

**Comparison**	**Mean difference (95% CI)**	**Certainty in treatment effects**	**Plain text summary**
Dulaglutide	-1.05 kPa (-	Moderate due to	Dulaglutide therapy probably has

	3.07,0.97) ⊕⊕⊕⊖	serious indirectness	no effect on LSM levels.
Semaglutide	-3.08 kPa (-3.39,-2.77) ⊕⊕⊕⊕	High	Semaglutide therapy could reduce LSM levels.
Dapagliflozin	-1.67 kPa (-4.49,1.16) ⊕⊕⊕⊖	Moderate due to serious indirectness	Dapagliflozin therapy probably has no effect on LSM levels.
Empagliflozin	-0.49 kPa (-1.11,0.12) ⊕⊕⊕⊖	Moderate due to serious inconsistency	Empagliflozin therapy probably has no effect on LSM levels.
Tofogliflozin	0.46 kPa (-0.89,1.82) ⊕⊕⊕⊖	Moderate due to serious indirectness	Tofogliflozin therapy probably has no effect on LSM levels.

CI= confidence interval.

Table 9 Body weight (BW)

**Comparison**	**Mean difference (95% CI)**	**Certainty in treatment effects**	**Plain text summary**
Liraglutide	-3.75 kg (-6.34,-1.15) ⊕⊕⊕⊖	Moderate due to serious inconsistency	Liraglutide therapy probably reduces body weight.
Exenatide	-4.40 kg (-8.12,-0.69) ⊕⊕⊖⊖	Low due to serious risk of bias and indirectness	Exenatide therapy may reduce body weight.
Dulaglutide	-2.14 kg (-8.45,4.16) ⊕⊕⊕⊖	Moderate due to serious indirectness	Dulaglutide therapy probably has no effect on body weight.

Semaglutide	-8.14 kg (-11.45,-4.84) ⨁⨁⨁⨁	High	Semaglutide therapy could reduce body weight.
Dapagliflozin	-3.48 kg (-5.88,-1.08) ⨁⨁⨁⨁	High	Dapagliflozin therapy could reduce body weight.
Empagliflozin	-2.60 kg (-7.31,2.12) ⨁⨁⨁⨁	High	Empagliflozin therapy has no effect on body weight.
Ipragliflozin	-3.04 kg (-7.54,1.47) ⨁⨁⨁⊖	Moderate due to serious risk of bias	Ipragliflozin therapy probably has no effect on body weight.
Tofogliflozin	1.60 kg (-4.71,7.90) ⨁⨁⨁⊖	Moderate due to serious indirectness	Tofogliflozin therapy probably has no effect on body weight.

CI= confidence interval.

Table 10 Body mass index (BMI)

**Comparison**	**Mean difference (95% CI)**	**Certainty in treatment effects**	**Plain text summary**
Liraglutide	-1.49 kg/m² (-2.39,-0.59) ⨁⨁⨁⨁	High	Liraglutide therapy could reduce BMI.
Exenatide	-1.86 kg/m² (-3.12,-0.59) ⨁⨁⊖⊖	Low due to serious risk of bias and indirectness	Exenatide therapy may reduce BMI.
Dulaglutide	-1.00 kg/m² (-3.10,1.09)	Moderate due to serious indirectness	Dulaglutide therapy probably has no effect on BMI.

	⊕⊕⊕⊖		
Dapagliflozin	-1.13 kg/m² (-2.14,-0.11) ⊕⊕⊕⊖	Moderate due to serious risk of bias	Dapagliflozin therapy probably reduces BMI.
Empagliflozin	-1.07 kg/m² (-2.45,0.31) ⊕⊕⊕⊕	High	Empagliflozin therapy has no effect on BMI.
Ipragliflozin	-1.07 kg/m² (-2.64,0.50) ⊕⊕⊕⊖	Moderate due to serious risk of bias	Ipragliflozin therapy probably has no effect on BMI.
Luseogliflozin	-0.83 kg/m² (-2.83,1.16) ⊕⊖⊖⊖	Very low due to very serious risk of bias and serious indirectness	Whether luseogliflozin therapy has an effect on BMI is uncertain.

CI= confidence interval.

Table 11 Waist circumference (WC)

Comparison	Mean difference (95% CI)	Certainty in treatment effects	Plain text summary
Liraglutide	-4.53 cm (-7.37,-1.69) ⊕⊕⊕⊖	Moderate due to serious risk of bias	Liraglutide therapy probably reduces waist circumference.
Exenatide	-6.12 cm (-10.26,-1.97) ⊕⊕⊖⊖	Low due to serious risk of bias and indirectness	Exenatide therapy may reduce waist circumference.
Dapagliflozin	-2.78 cm (-5.61,0.06) ⊕⊕⊕⊕	High	Dapagliflozin therapy has no effect on waist circumference.

Empagliflozin	-1.30 cm (-7.54,4.94) ⊕⊕⊕⊕	High	Empagliflozin therapy has no effect on waist circumference.
Ipragliflozin	-3.70 cm (-8.80,1.40) ⊕⊕⊖⊖	Low due to very serious risk of bias	Ipragliflozin therapy may have no effect on waist circumference.

CI= confidence interval.

Table 12 Systobic blood pressure (SBP)

Comparison	Mean difference (95% CI)	Certainty in treatment effects	Plain text summary
Liraglutide	-3.27 mmHg (-7.45,0.91) ⊕⊕⊕⊕	High	Liraglutide therapy has no effect on systobic blood pressure.
Exenatide	-3.11 mmHg (-8.46,2.25) ⊕⊖⊖⊖	Very low due to very serious risk of bias and serious indirectness	Whether exenatide therapy has an effect on systobic blood pressure is uncertain.
Semaglutide	-2.24 mmHg (-4.20,-0.27) ⊕⊕⊕⊕	High	Semaglutide therapy could reduce systobic blood pressure.
Dapagliflozin	-3.61 mmHg (-9.84,2.61) ⊕⊕⊕⊖	Moderate due to serious indirectness	Dapagliflozin therapy probably has no effect on systobic blood pressure.
Empagliflozin	5.09 mmHg (-4.90,15.09) ⊕⊕⊕⊖	Moderate due to serious indirectness	Empagliflozin therapy probably has no effect on systobic blood pressure.
Ipragliflozin	-3.90 mmHg (-	Low due to very	Ipragliflozin therapy may reduce

	6.79,-1.01) ⊕⊕⊖⊖	serious risk of bias	systobic blood pressure.

CI= confidence interval.

Table 13 Diastolic blood pressure (DBP)

Comparison	Mean difference (95% CI)	Certainty in treatment effects	Plain text summary
Liraglutide	0.24 mmHg (-2.76,3.24) ⊕⊕⊕⊕	High	Liraglutide therapy has no effect on diastolic blood pressure.
Exenatide	-0.77 mmHg (-4.14,2.60) ⊕⊖⊖⊖	Very low due to very serious risk of bias and serious indirectness	Whether exenatide therapy has an effect on diastolic blood pressure is uncertain.
Semaglutide	-0.38 mmHg (-1.41,0.64) ⊕⊕⊕⊕	High	Semaglutide therapy has no effect on diastolic blood pressure.
Dapagliflozin	-1.59 mmHg (-5.41,2.23) ⊕⊕⊕⊖	Moderate due to serious indirectness	Dapagliflozin therapy probably has no effect on diastolic blood pressure.
Empagliflozin	1.93 mmHg (-4.47,8.33) ⊕⊕⊕⊖	Moderate due to serious indirectness	Empagliflozin therapy probably has no effect on diastolic blood pressure.
Ipragliflozin	0.33 mmHg (-1.50,2.17) ⊕⊕⊖⊖	Low due to very serious risk of bias	Ipragliflozin therapy may have no effect on diastolic blood pressure.

CI= confidence interval.

Table 14 Blood lipids total cholesterol (TC)

Comparison	Mean difference (95% CI)	Certainty in treatment effects	Plain text summary
Liraglutide	-0.24 mmol/L (-0.55,0.06) ⊕⊕⊕⊖	Moderate due to serious inconsistency	Liraglutide therapy probably has no effect on TC levels.
Exenatide	-0.33 mmol/L (-0.74,0.07) ⊕⊕⊖⊖	Low due to serious risk of bias and indirectness	Exenatide therapy may have no effect on TC levels.
Semaglutide	0.13 mmol/L (-0.13,0.39) ⊕⊕⊕⊕	High	Semaglutide therapy has no effect on TC levels.
Dapagliflozin	0.30 mmol/L (-0.19,0.78) ⊕⊖⊖⊖	Very low due to very serious risk of bias and serious indirectness	Whether dapagliflozin therapy has an effect on TC levels is uncertain.
Empagliflozin	-0.23 mmol/L (-0.76,0.30) ⊕⊕⊕⊕	High	Empagliflozin therapy has no effect on TC levels.
Ipragliflozin	0.05 mmol/L (-0.28,0.38) ⊕⊕⊖⊖	Low due to serious risk of bias and inconsistency	Ipragliflozin therapy may have no effect on TC levels.
Tofogliflozin	-0.01 mmol/L (-0.81,0.79) ⊕⊕⊕⊖	Moderate due to serious indirectness	Tofogliflozin therapy probably has no effect on TC levels.

CI= confidence interval.

Table 15 Triglycerides (TG)

Comparison	Mean difference (95% CI)	Certainty in treatment effects	Plain text summary

Liraglutide	-0.21 mmol/L (-0.44,0.02) ⊕⊕⊕⊖	Moderate due to serious inconsistency	Liraglutide therapy probably has no effect on TG levels.
Exenatide	-0.22 mmol/L (-0.44,0.05) ⊕⊕⊖⊖	Low due to serious risk of bias and indirectness	Exenatide therapy may have no effect on TG levels.
Dulaglutide	-0.36 mmol/L (-2.77,2.06) ⊕⊕⊕⊖	Moderate due to serious indirectness	Dulaglutide therapy probably has no effect on TG levels.
Semaglutide	-0.25 mmol/L (-0.40,-0.10) ⊕⊕⊕⊕	High	Semaglutide therapy could reduce TG levels.
Dapagliflozin	-0.22 mmol/L (-0.46,0.01) ⊕⊕⊖⊖	Low due to serious risk of bias and inconsistency	Dapagliflozin therapy may have no effect on TG levels.
Empagliflozin	-0.09 mmol/L (-0.60,0.41) ⊕⊕⊕⊕	High	Empagliflozin therapy has no effect on TG levels.
Ipragliflozin	-0.26 mmol/L (-0.48,-0.04) ⊕⊕⊕⊖	Moderate due to serious risk of bias	Ipragliflozin therapy probably reduces TG levels.
Tofogliflozin	0.52 mmol/L (-0.12,1.16) ⊕⊕⊕⊖	Moderate due to serious indirectness	Tofogliflozin therapy probably has no effect on TG levels.

CI= confidence interval.

Table 16 High density lipoprotein-cholesterol (HDL-C)

Comparison	Mean difference (95% CI)	Certainty in treatment effects	Plain text summary

Liraglutide	0.08 mmol/L (0.02,0.15) ⊕⊕⊕⊖	Moderate due to serious inconsistency	Liraglutide therapy probably increase HDL-C levels.
Exenatide	0.07 mmol/L (-0.02,0.17) ⊕⊕⊕⊖	Moderate due to serious indirectness	Exenatide therapy probably has no effect on HDL-C levels.
Dulaglutide	-0.02 mmol/L (-0.58,0.54) ⊕⊕⊕⊖	Moderate due to serious indirectness	Dulaglutide therapy probably has no effect on HDL-C levels.
Semaglutide	0.05 mmol/L (0.01,0.09) ⊕⊕⊕⊕	High	Semaglutide therapy could increase HDL-C levels.
Dapagliflozin	0.14 mmol/L (0.06,0.21) ⊕⊕⊖⊖	Low due to serious risk of bias and inconsistency	Dapagliflozin therapy may increase HDL-C levels.
Empagliflozin	0.03 mmol/L (-0.07,0.14) ⊕⊕⊕⊕	High	Empagliflozin therapy has no effect on HDL-C levels.
Ipragliflozin	0.08 mmol/L (0.01,0.14) ⊕⊕⊕⊖	Moderate due to serious risk of bias	Ipragliflozin therapy probably increases HDL-C levels.

CI= confidence interval.

Table 17 Low density lipoprotein-cholesterol (LDL-C)

**Comparison**	**Mean difference (95% CI)**	**Certainty in treatment effects**	**Plain text summary**
Liraglutide	-0.05 mmol/L (-0.35,0.25)	Moderate due to	Liraglutide therapy probably has

	⊕⊕⊕⊖	serious inconsistency	no effect on LDL-C levels.
Exenatide	-0.28 mmol/L (-0.73,0.18) ⊕⊕⊕⊖	Moderate due to serious indirectness	Exenatide therapy probably has no effect on LDL-C levels.
Dulaglutide	0.10 mmol/L (-2.11,2.31) ⊕⊕⊕⊖	Moderate due to serious indirectness	Dulaglutide therapy probably has no effect on LDL-C levels.
Semaglutide	0.16 mmol/L (-0.16,0.48) ⊕⊕⊕⊕	High	Semaglutide therapy has no effect on LDL-C levels.
Dapagliflozin	-0.03 mmol/L (-0.37,0.31) ⊕⊕⊕⊕	High	Dapagliflozin therapy has no effect on LDL-C levels.
Empagliflozin	-0.09 mmol/L (-0.55,0.37) ⊕⊕⊕⊕	High	Empagliflozin therapy has no effect on LDL-C levels.
Ipragliflozin	0.06 mmol/L (-0.33,0.46) ⊕⊕⊕⊖	Moderate due to serious inconsistency	Ipragliflozin therapy probably has no effect on LDL-C levels.

CI= confidence interval.

Table 18 Serum adiponectin

**Comparison**	**Mean difference (95% CI)**	**Certainty in treatment effects**	**Plain text summary**
Liraglutide	3.03μg/ml (-0.43,6.49) ⊕⊕⊕⊕	High	Liraglutide therapy has no effect on serum adiponectin.

Exenatide	-1.59µg/ml (-6.13,2.96) ⊕⊕⊕⊖	Moderate due to serious indirectness	Exenatide therapy probably has no effect on serum adiponectin.
Dapagliflozin	-1.61µg/ml (-5.02,1.81) ⊕⊕⊕⊖	Moderate due to serious inconsistency	Dapagliflozin therapy probably has no effect on serum adiponectin.
Ipragliflozin	-7.18µg/ml (-13.13,-1.23) ⊕⊖⊖⊖	Very low due to very serious risk of bias and serious indirectness	Whether ipragliflozin therapy has an effect on serum adiponectin is uncertain.

CI= confidence interval.

Table 19 Fasting blood glucose (FBG)

**Comparison**	**Mean difference (95% CI)**	**Certainty in treatment effects**	**Plain text summary**
Liraglutide	-0.77 mmol/L (-1.19,-0.35) ⊕⊕⊕⊕	High	Liraglutide therapy could reduce FBG levels.
Exenatide	-0.36 mmol/L (-0.91,0.20) ⊕⊕⊖⊖	Low due to serious risk of bias and indirectness	Exenatide therapy may have no effect on FBG levels.
Dulaglutide	-1.32 mmol/L (-10.13,7.49) ⊕⊕⊕⊖	Moderate due to serious indirectness	Dulaglutide therapy probably has no effect on FBG levels.
Semaglutide	-1.35 mmol/L (-3.01,0.32) ⊕⊕⊕⊕	High	Semaglutide therapy has no effect on FBG levels.
Dapagliflozin	-0.75 mmol/L (-	High	Dapagliflozin therapy could

	1.12,-0.39)<br>⊕⊕⊕⊕		reduce FBG levels.
Empagliflozin	0.06 mmol/L (-0.44,0.55)<br>⊕⊕⊕⊖	Moderate due to serious inconsistency	Empagliflozin therapy probably has no effect on FBG levels.
Ipragliflozin	-0.33 mmol/L (-0.84,0.18)<br>⊕⊕⊕⊖	Moderate due to serious risk of bias	Ipragliflozin therapy probably has no effect on FBG levels.
Tofogliflozin	0.67 mmol/L (-0.95,2.29)<br>⊕⊕⊕⊖	Moderate due to serious indirectness	Tofogliflozin therapy probably has no effect on FBG levels.
Luseogliflozin	-2.32 mmol/L (-16.74,12.10)<br>⊕⊖⊖⊖	Very low due to very serious risk of bias and serious indirectness	Whether luseogliflozin therapy has an effect on FBG levels is uncertain.

CI= confidence interval.

Table 20 Postprandial blood glucose (PBG)

**Comparison**	**Mean difference (95% CI)**	**Certainty in treatment effects**	**Plain text summary**
Liraglutide	-1.70 mmol/L (-3.74,0.34)<br>⊕⊕⊕⊖	Moderate due to serious indirectness	Liraglutide therapy probably has no effect on PBG levels.
Exenatide	-0.15 mmol/L (-2.32,2.02)<br>⊕⊕⊖⊖	Low due to serious risk of bias and indirectness	Exenatide therapy may have no effect on PBG levels.
Dapagliflozin	-2.14 mmol/L (-3.67,-0.61)	High	Dapagliflozin therapy could reduce PBG levels.

	⊕⊕⊕⊕		

CI= confidence interval.

Table 21 Glycosylated hemoglobin (HbA1c)

**Comparison**	**Mean difference (95% CI)**	**Certainty in treatment effects**	**Plain text summary**
Liraglutide	-0.50% (-0.81,-0.19) ⊕⊕⊕⊕	High	Liraglutide therapy could reduce HbA1c levels.
Exenatide	-0.41% (-0.82,0.00) ⊕⊕⊖⊖	Low due to serious risk of bias and indirectness	Exenatide therapy may reduce HbA1c levels.
Dulaglutide	-0.65% (-1.47,0.17) ⊕⊕⊕⊖	Moderate due to serious indirectness	Dulaglutide therapy probably has no effect on HbA1c levels.
Semaglutide	-0.93% (-1.23,-0.63) ⊕⊕⊕⊕	High	Semaglutide therapy could reduce HbA1c levels.
Dapagliflozin	-0.72% (-1.01,-0.42) ⊕⊕⊕⊕	High	Dapagliflozin therapy could reduce HbA1c levels.
Empagliflozin	-0.12% (-0.64,0.39) ⊕⊕⊕⊖	Moderate due to serious inconsistency	Empagliflozin therapy probably has no effect on HbA1c levels.
Ipragliflozin	-0.36% (-0.77,0.04) ⊕⊕⊕⊖	Moderate due to serious risk of bias	Ipragliflozin therapy probably has no effect on HbA1c levels.

Tofogliflozin	-0.06% (-0.67,0.55) ⊕⊕⊕⊖	Moderate due to serious indirectness	Tofogliflozin therapy probably has no effect on HbA1c levels.
Luseogliflozin	-1.05% (-1.80,-0.30) ⊕⊖⊖⊖	Very low due to very serious risk of bias and serious indirectness	Whether luseogliflozin therapy has an effect on HbA1c levels is uncertain.

CI= confidence interval.

Table 22 Glucose and homeostasis model assessment (HOMA-IR)

Comparison	Mean difference (95% CI)	Certainty in treatment effects	Plain text summary
Liraglutide	-1.57 (-2.18,-0.96) ⊕⊕⊕⊕	High	Liraglutide therapy could reduce HOMA-IR.
Exenatide	-0.34 (-1.53,0.86) ⊕⊕⊕⊖	Moderate due to serious indirectness	Exenatide therapy probably has no effect on HOMA-IR.
Semaglutide	-0.29 (-1.19,0.61) ⊕⊕⊕⊕	High	Semaglutide therapy has no effect on HOMA-IR.
Dapagliflozin	-0.84 (-1.53,-0.15) ⊕⊕⊕⊕	High	Dapagliflozin therapy could reduce HOMA-IR.
Empagliflozin	-0.11 (-0.49,0.27) ⊕⊕⊕⊖	Moderate due to serious inconsistency	Empagliflozin therapy probably has no effect on HOMA-IR.
Ipragliflozin	-0.60 (-1.01,-0.19) ⊕⊕⊕⊖	Moderate due to serious risk of bias	Ipragliflozin therapy probably reduces HOMA-IR.

CI= confidence interval.
